# Antibacterial phenolic compounds from the flowering plants of Asia and the Pacific: coming to the light

**DOI:** 10.1080/13880209.2024.2407530

**Published:** 2024-10-11

**Authors:** Mazdida Sulaiman, Layane Ebehairy, Veeranoot Nissapatorn, Mohammed Rahmatullah, Jhonnel Villegas, Helina Jean Dupa, Ricksterlie C. Verzosa, Karma G. Dolma, Muhamad Shabaz, Scholastica Lanting, Nor Azizun Rusdi, Nor Hayati Abdullah, Mohammed Khaled Bin Break, Teng Jin Khoo, Wei Wang, Christophe Wiart

**Affiliations:** a Department of Chemistry, Faculty of Science, Universiti Malaya, Kuala Lumpur, Malaysia; b School of Allied Health Sciences, Walailak University, Nakhon Si Thammarat, Thailand; c Department of Biotechnology, University of Development Alternative, Dhaka, Bangladesh; d Faculty of Education and Teacher Training, Davao Oriental State University, Mati, Philippines; e Faculty of Agriculture and Life Science, Davao Oriental State University, Mati, Philippines; f Department of Microbiology, Sikkim Manipal University, Gangtok, India; g Institute for Tropical Biology and Conservation, Universiti Malaysia Sabah, Kota Kinabalu, Malaysia; h Natural Product Division, Forest Research Institute of Malaysia, Kepong, Malaysia; i Department of Pharmaceutical Chemistry, College of Pharmacy, University of Ha’il, Ha’il, Saudi Arabia; j School of Pharmacy, University of Nottingham Malaysia, Semenyih, Malaysia; k School of Pharmacy, Hunan University of Chinese Medicine, Changsha, China

**Keywords:** Angiosperms, antibiotics, Asia-Pacific, inflammation, superbugs

## Abstract

**Context:**

The emergence of pan-resistant bacteria requires the development of new antibiotics and antibiotic potentiators.

**Objective:**

This review identifies antibacterial phenolic compounds that have been identified in Asian and Pacific Angiosperms from 1945 to 2023 and analyzes their strengths and spectra of activity, distributions, molecular masses, solubilities, modes of action, structures-activities, as well as their synergistic effects with antibiotics, toxicities, and clinical potential.

**Methods:**

All data in this review was compiled from Google Scholar, PubMed, Science Direct, Web of Science, and library search; other sources were excluded. We used the following combination of keywords: ‘Phenolic compound’, ‘Plants’, and ‘Antibacterial’. This produced 736 results. Each result was examined and articles that did not contain information relevant to the topic or coming from non-peer-reviewed journals were excluded. Each of the remaining 467 selected articles was read critically for the information that it contained.

**Results:**

Out of ∼350 antibacterial phenolic compounds identified, 44 were very strongly active, mainly targeting the cytoplasmic membrane of Gram-positive bacteria, and with a molecular mass between 200 and 400 g/mol. 2-Methoxy-7-methyljuglone, [6]-gingerol, anacardic acid, baicalin, vitexin, and malabaricone A and B have the potential to be developed as antibacterial leads.

**Conclusions:**

Angiosperms from Asia and the Pacific provide a rich source of natural products with the potential to be developed as leads for treating bacterial infections.

## Introduction

The resistance of bacteria to antibiotics has increased to the point that treating nosocomial infections in intensive care units has become difficult, and in some cases even impossible. The list of bacterial strains resistant to antibiotics continues to grow. The intrinsic mechanisms of resistance in Gram-negative bacteria to antibiotics include, at least in part, an outer lipopolysaccharides coat carrying a net negative charge which halts (partially) the entry of negatively charged molecules (Denyer and Maillard 2002; van den Berg 2010) as well as porins preventing the penetration of lipophilic molecules (Bauer et al. 1988; van den Berg 2010). Bacteria keep on exchanging genes (among other things *via* plasmid transfer) coding for efflux pumps, such as NorA (Sun et al. 2020), TetK in *Staphylococcus aureus* (Macêdo et al. 2022), and AcrAB in *Escherichia coli* (Kuete et al. 2010). Other resistance mechanisms acquired through gene exchange include enzymes that inactivate antibiotics (β-lactamases) (Eumkeb et al. 2010; Siriwong et al. 2015) and structurally altered bacterial targets (Oyedemi et al. 2016).

The mode of action of antibiotics, which target a specific bacterial macromolecule or enzyme, sooner or later leads to the inevitable development of resistance. Some superbugs have accumulated genes of resistance to almost all known antibiotics (Willyard 2017). Examples are *Mycobacterium tuberculosis* (clinical isolate CIBIN 99) (Uc-Cachón et al. 2014), methicillin-resistant *S. aureus* (MRSA) SCCmec III (Asghar 2014) and USA300 strains (Carrel et al. 2015), *Stenotrophomonas maltophilia* (Gordon and Wareham 2010), and vancomycin-resistant enterococcus (VRE) (Tan, Hua, et al. 2020). *Acinetobacter baumannii*, which began as a hospital commensal bacterium, has transformed over the last decades into a bacterium resistant to almost all antibiotics (Osterburg et al. 2009). In 2016, the World Health Organization (WHO) listed carbapenem-resistant *A. baumannii* as first on the list of bacteria posing a threat to human health (Willyard 2017). In 2019, Nichols described the case of a 48-year-old man succumbing to a pan-resistant *A. baumannii* following a lung transplant (Nichols 2019). The number of bacterial strains on the verge of becoming resistant to all antibiotics continues to increase inexorably, and among them is *E. coli* O157:H7 posing the risk of incurable food poisoning (Haile et al. 2022).

Identifying antibacterial molecules with chemical structures completely different from those of antibiotics currently in use and capable of evading or inhibiting bacterial resistance is an urgent necessity (Chusri et al. 2009). There are numerous sources of antibacterial compounds in the living world, particularly in flowering plants. Flowering plants also called Angiosperms are organized into 11 major taxa or clades distributed into three groups: (i) basal Angiosperms (protomagnoliids, magnoliids, monocots, and eudicots), (ii) core Angiosperms (core eudicots, rosids, fabids, and malvids), and (iii) upper Angiosperms (asterids, lamiids, and campanulids) (Byng et al. 2009). Within each clade, plants are grouped into different orders, families, genera, and species producing secondary metabolites, one of those roles is often to fight against bacterial infections. These antibacterial natural products are either present in plants before bacterial infection (phytoanticipins) or synthesized during bacterial infection (phytoalexins) (Van Etten et al. 1994). Compared to antibiotics, they do not have a single bacterial target (Yuan et al. 2021). Their antibacterial activity is *in vitro* qualitatively evaluated using paper discs or agar wells and quantified by calculating the minimum inhibitory concentration (MIC) and minimum bactericidal concentration (MBC). When the MBC/MIC ratio is ≤4, the compounds are bactericidal whereas a MBC/MIC ≥ 8 indicates a bacteriostatic effect (Huang et al. 2021). Phytoanticipins and phytoalexins can act synergistically with antibiotics, in which case the fractional inhibitory concentration index (FICI) is <0.5 (Miklasińska-Majdanik et al. 2018). These antibacterial agents belong to three major phytochemical groups: alkaloids, terpenes, and phenolic compounds.

Phenolic compounds are structurally defined by a benzene ring substituted by at least one hydroxyl group. They are organized into two categories: non-flavonoids and flavonoids, and they occupy a major role in plant defense against bacteria (Weinstein and Albersheim 1983). Several observations made since the 1940s indicate that phenolic compounds can escape acquired bacterial resistance. As early as 1945, Fogg and Lodge observed that *Enterobacter aerogenes* could not develop resistance to phenolic compounds (Fogg and Lodge 1945). More recently, Chen et al. (2018) reported the almost impossibility of *S. aureus* to develop resistance against a phenolic compound identified from an Asian orchid used in traditional Chinese medicine. At the same time, there is growing evidence that phenolic compounds can weaken the resistance of bacteria to antibiotics and enhance the activity of antibiotics.

The hypothesis of using phenolic compounds as a source of antibacterial molecules to treat bacterial infections has been recently raised (Ecevit et al. 2022; Sun and Shahrajabian 2023). In this context, this comprehensive and scholarly evidence-based review aims to cover, organize, and correlate the data accumulated from 1945 to 2023 regarding the antibacterial phenolic compounds identified from flowering plants from Asia and the Pacific. This review covers the distribution, strength, influence of molecular mass and solubility, structure-activity, mechanisms of action, synergistic activity with antibiotics, toxicity, and clinical potential. This review provides a taxonomical, phytochemical, biomolecular, and physicochemical rationale to facilitate the discovery of leads for treating bacterial infections. All data in this review was compiled from Google Scholar, PubMed, Science Direct, Web of Science, and library search, other sources were excluded. We used the following combination of keywords: ‘Phenolic compound’, ‘Plants’, and ‘Antibacterial’. Each result was examined and articles that did not contain information relevant to the topic or coming from non-peer-reviewed journals were excluded. The remaining selected articles were read critically for the information that they contained.

## Non-flavonoids

### Hydroxycinnamic acid derivatives

**Figure 1.**
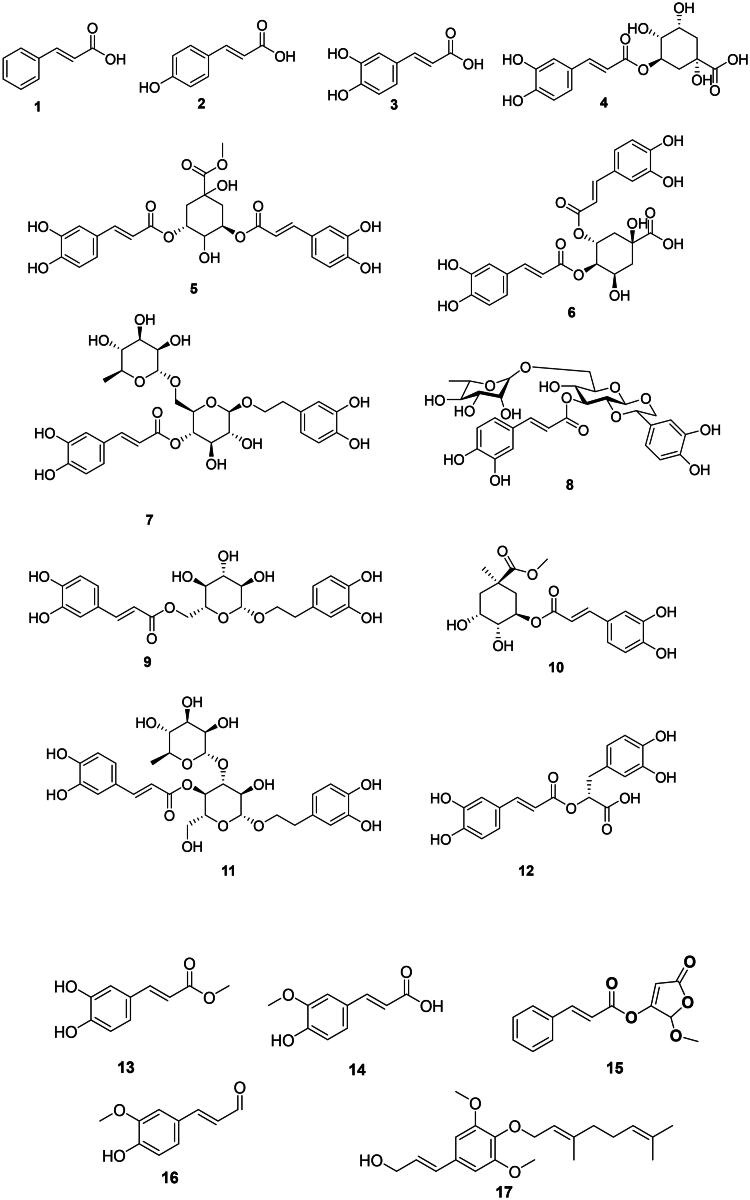
Hydroxycinnamic acid derivatives.

They are derived from l-phenyl alanine and are found primarily in the monocots and upper Angiosperms (Figure 1). They have weak but broad-spectrum antibacterial activity. Examples are cinnamic acid (**1**) from *Cinnamomum zeylanicum* Bl. (Lauraceae, magnoliids) and *p*-coumaric acid (**2**) (Guzman 2014). Caffeic acid (**3**) from *Plantago major* L. (Plantaginaceae, lamiids) was active against *Pseudomonas aeruginosa* (MIC: 31.3 μg/mL) (Perumal et al. 2015; Kępa et al. 2018).

In upper Angiosperms, caffeic acid forms esters with quinic acid, such as chlorogenic acid (**4**) (*Klebsiella pneumoniae*) (Cai et al. 2019), 3,5-di-*O*-caffeoylquinic acid (**5**), and 4,5-di-*O*-caffeoylquinic (**6**) from *Lonicera japonica* Thunb. (Caprifoliaceae, campanulids) which is a plant used in traditional Chinese medicine (Xiong et al. 2013). Forsythiaside (**7**) from *Forsythia suspensa* (Thunb.) Vahl (Oleaceae, lamiids) inhibited the growth of *E. coli*, *P. aeruginosa*, and *S. aureus* with MIC values of 38.3, 38.3, and 76.6 µg/mL, respectively (Qu et al. 2008). From this plant, lianqiaoxinoside B (**8**) was active against *Bacillus dysenteriae* (MIC: 36.7 µg/mL) (Kuang et al. 2011). Other examples include calceolarioside B (**9**) from *Sargentodoxa cuneata* (Oliv.) Rehder and E.H. Wilson (Lardizabalaceae, eudicots) with *S. aureus* (MIC: 64 µg/mL), methyl 3-*O-*caffeoylquinate (**10**) with of *S. aureus* (MIC: 32 µg/mL) (Zeng et al. 2015), and verbascoside (**11**) from *Stachytarpheta indica* (L.) Vahl (Verbenaceae, lamiids) with *Enterococcus faecalis*, *Shigella sonnei* (MIC: 31.2 µg/mL) (Nguyen et al. 2018), and *Staphylococcus* sp. (MIC: 9.7 µg/mL) (Agampodi et al. 2022).

The coupling of two caffeic acid units forms rosmarinic acid (**12**) in *Rosmarinus officinalis* L. (Lamiaceae, lamiids). Rosmarinic acid (**12**) has weak but broad-spectrum bactericidal activity (Abedini et al. 2013). The esterification of caffeic acid forms methyl caffeate (**13**) in *Solanum torvum* Sw. (Solanaceae, lamiids) active against rifampicin-resistant *M. tuberculosis* (MIC: 8 μg/mL) (Balachandran et al. 2012). Methoxylation of caffeic acid in position 3 gives ferulic acid (**14**) (Guzman 2014) weakly bactericidal for *S. aureus* and *E. coli* (Cai et al. 2019).

Other examples are 2-methoxy-2-butenolide-3-cinnamate (**15**) from *Polygonum glabrum* Willd. (Polygonaceae, malvids) (*M. tuberculosis*, MIC: 1.4 µg/mL) (Said et al. 2015), coniferylaldehyde (**16**) from *Ficus benghalensis* L. (Moraceae, fabids) bactericidal for *Streptococcus mutans* (MIC/MBC: 62.5/62.5 µg/mL) (Meerungrueang and Panichayupakaranant 2014), and nelumol A (**17**) from *Toddalia asiatica* (L.) Lam. (Rutaceae, malvids) (*M. tuberculosis*, MIC: 50 µg/mL) (Phatchana and Yenjai 2014).

### Phenylpropanoids

**Figure 2.**
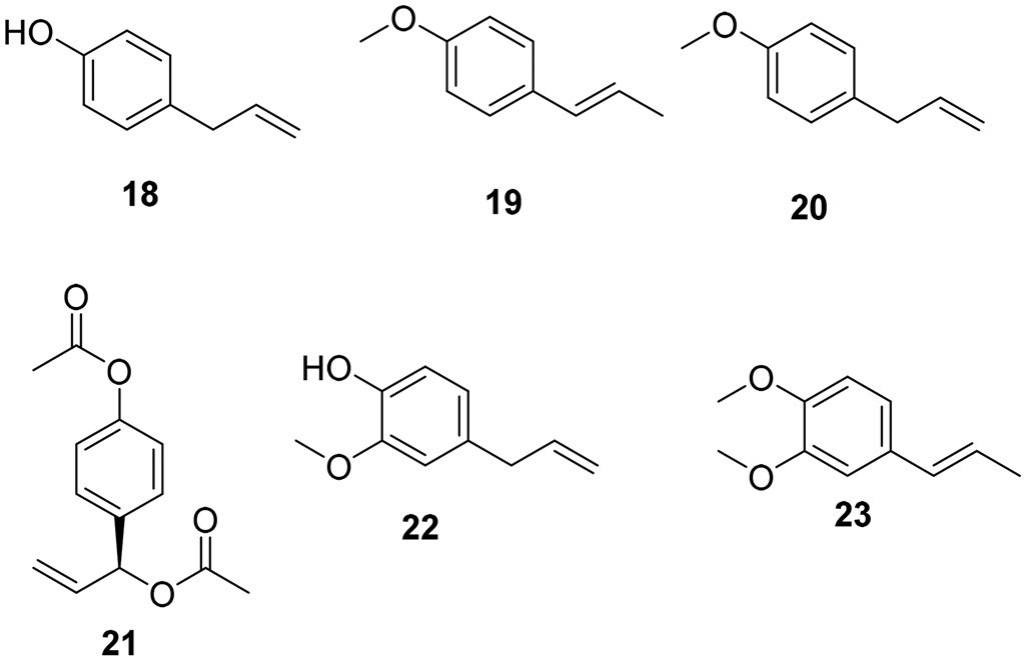
Phenylpropanoids.

*p*-Coumaric acid (**2**) is the precursor of antibacterial phenylpropanoids (Yu and Jez 2008) such as chavicol (**18**), anethole (**19**), and estragole (methyl chavicol) (**20**) (Atkinson 2016) (Figure 2). Anethole (**19**) was weakly bactericidal against *A. baumannii* (Newberne et al. 1999) and *Bacillus cereus* (MIC/MBC: 50/100 µg/mL) (Phanthong et al. 2013). 1′-Acetoxychavicol acetate (**21**) from *Alpinia galanga* (L.) Willd. (Zingiberaceae, monocots) inhibited the growth of *M. tuberculosis* with a MIC value as low as 0.7 µg/mL (Warit et al. 2017). Ferulic acid (**14**) is the precursor of eugenol (**22**) in *Eugenia aromatica* (L.) Baill. (Myrtaceae, malvids) active against *B. cereus* (MIC: 15.6 µg/mL), *E. coli* (MIC: 31.2 µg/mL) (Mohammed and Al-Bayati 2009), and *Vibrio parahaemolyticus* (Ashrafudoulla et al. 2020). Methylisoeugenol (**23**) from *Daucus carota* L (Apiaceae, campanulids) was effective against *Campylobacter jejuni* (Rossi et al. 2007).

### Coumarins

These chromene-2-ones originate from the *ortho*-hydroxylation and cyclization of cinnamic acid (**1**) (Shimizu 2014) (Figure 3).

Figure 3. Coumarins.

**Figure:**
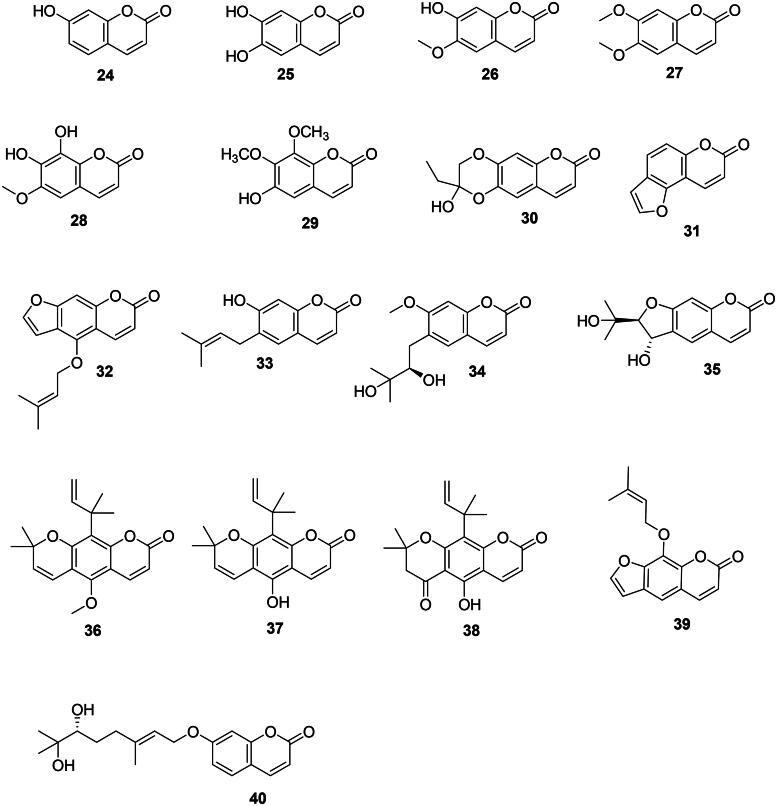

**Figure:**
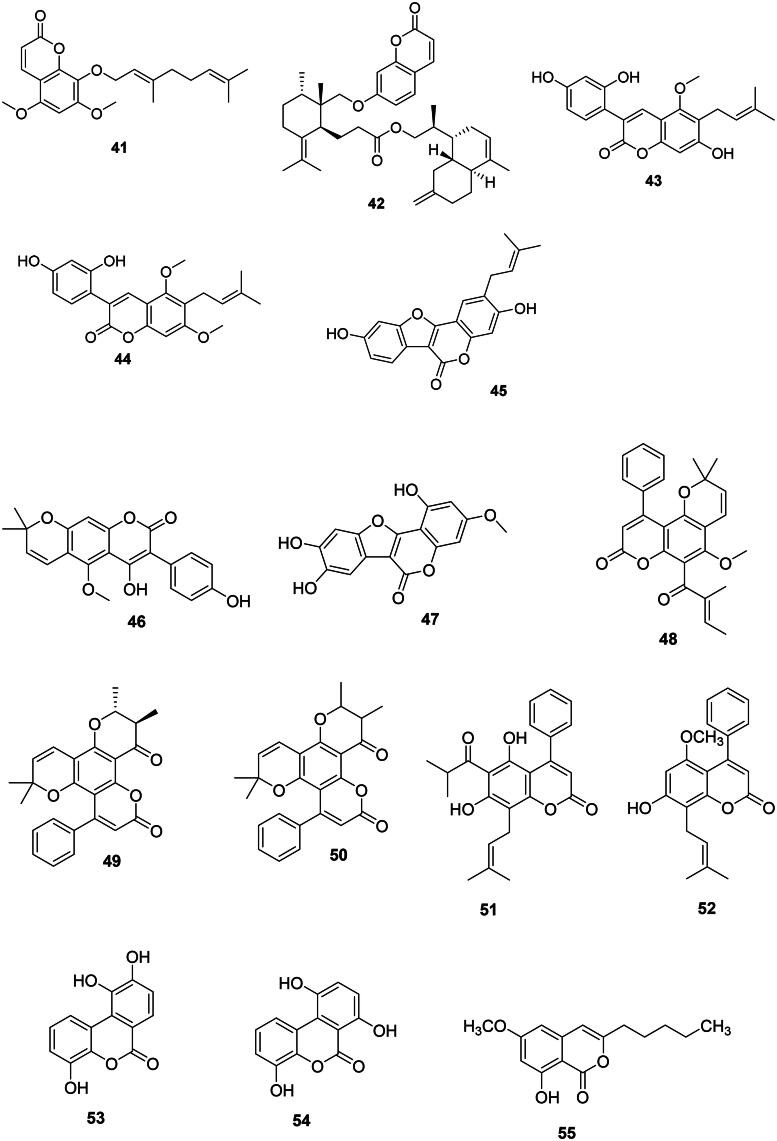

Figure 4. Stilbenes.

**Figure:**
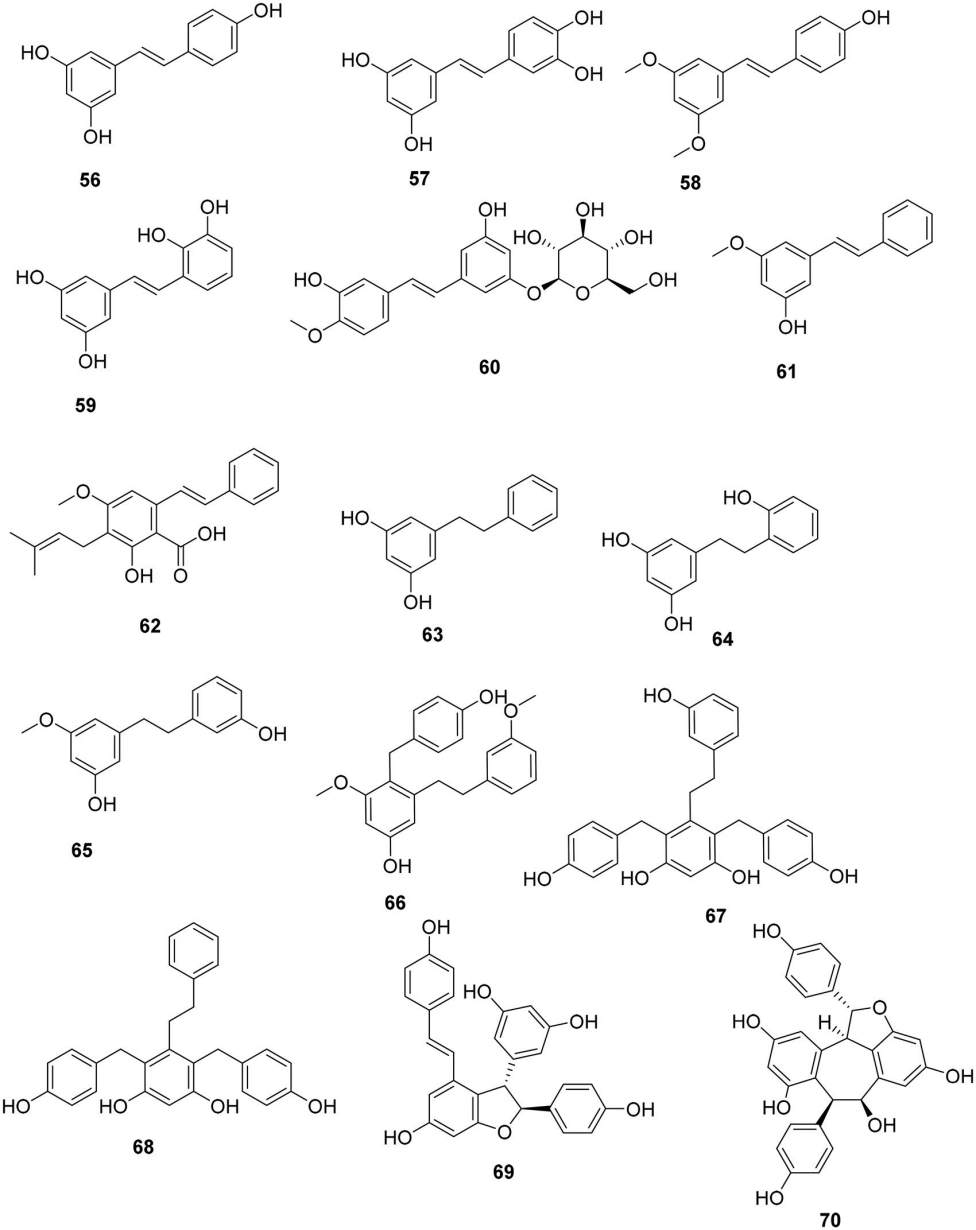

**Figure:**
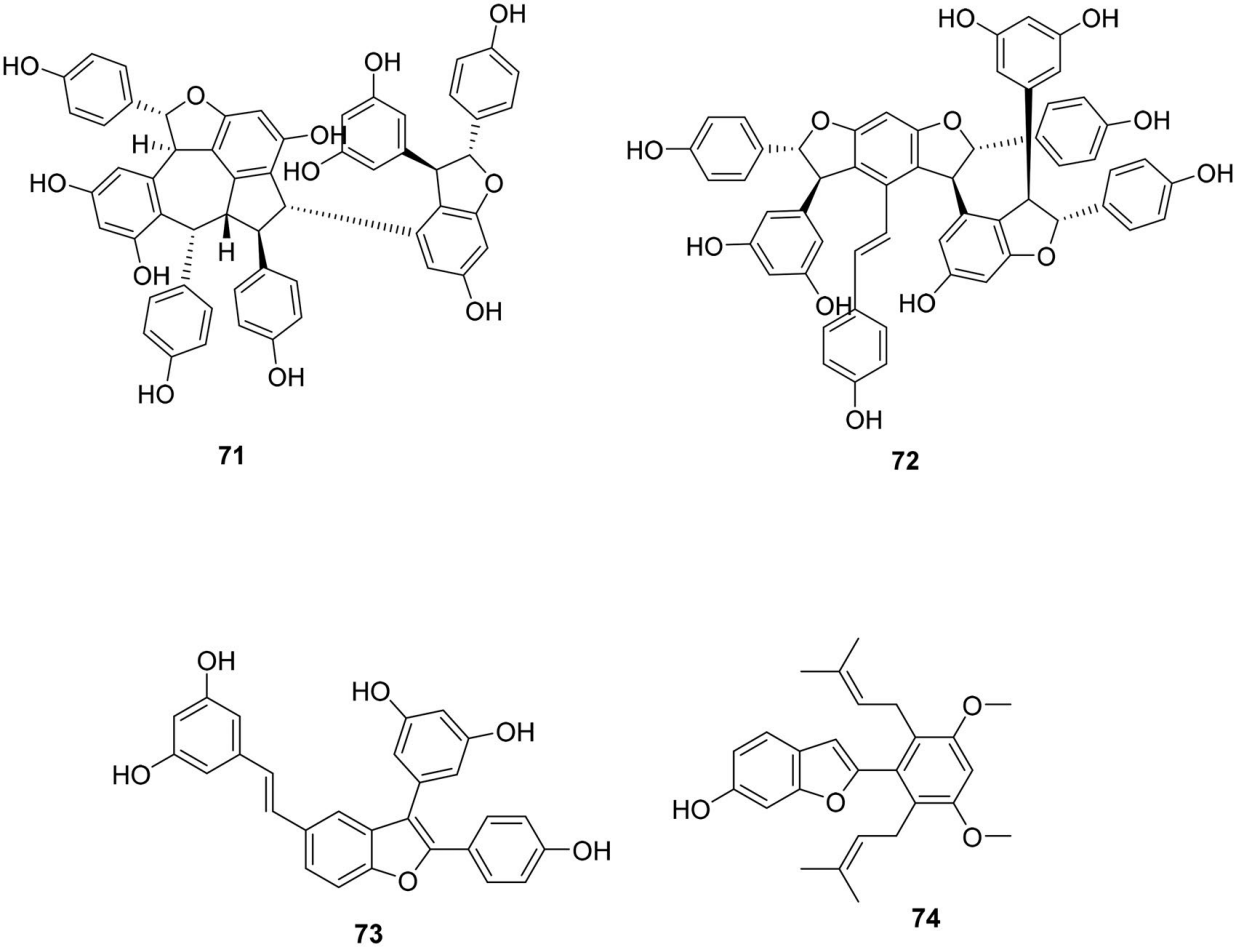

#### Simple coumarins

One of the simplest coumarins with one hydroxyl group at carbon 7 is umbelliferone (**24**) in *Acacia nilotica* (L.) Willd. ex Delile (Fabaceae, fabids) (Singh et al. 2010) bactericidal for *S. mutans* (MIC/MBC: 15.6/15.6 µg/mL) (Meerungrueang and Panichayupakaranant 2014). Addition of a hydroxyl group at carbon 8 of umbelliferone (**24**) forms esculetin (**25**) in *Viola prionantha* Bunge (Violaceae, fabids) active against *B. cereus* (MIC: 50 µg/mL) (Xie et al. 2004). Methoxylation of umbelliferone (**24**) in position 8 produces scopoletin (**26**) in *Pelargonium sidoides* (Geraniaceae, malvids) effective against *Mycobacterium smegmatis* (MIC: 7.8 µg/mL) (Mativandlela et al. 2007) as well as *S. aureus*, *Enterococcus faecium*, and *S. maltophilia* (Buathong et al. 2019). Scopoletin (**26**) and scoparone (**27**) from *Canarium pentatinervium* Miq. (Burseraceae, malvids) were bactericidal for *S. aureus* with MIC/MBC values of 25/50 and 50/100 µg/mL, respectively (Mogana et al. 2020; Mfonku et al. 2021). Other instances are fraxetin (**28**) from *Fraxinus rhynchophylla* Hance (Oleaceae, lamiids) (*S. aureus*) (Wang et al. 2014), 7,8-dimethoxy-6-hydroxy-coumarin (**29**) from *Haloxylon salicornicum* (Moq.) Bunge ex Boiss. (Amaranthaceae, malvids) (*M. tuberculosis*, MIC: 100 µg/mL) (Bibi et al. 2010), and euryacoumarin A (**30**) from *Eurya chinensis* R. Br. (Pentaphylacaceae, asterids) (Song et al. 2017).

#### Furanocoumarins

Examples are bakuchicin (**31**) from *Psoralea corylifolia* L. (Fabaceae) (Khatune et al. 2004) and isoimperatorin (**32**) from *Prangos hulusii* Şenol, Yıldırım & Seçmen (Apiaceae) (MRSA, MIC: 16 µg/mL) (Tan et al. 2017). Isoimperatorin (**32**) is antimycobacterial (Guo et al. 2014).

#### C-Prenylated coumarins

Antibacterial coumarins with an isoprene group at carbon 6 are found in the Rutaceae family. This is the case of desmethylsuberosine (**33**) in *Feronia lucida* Teijsm. & Binn. ex Scheff. (Rahman and Gray 2002). Another example is ulopterol (**34**) from *T. asiatica* with *Staphylococcus epidermidis* (MIC: 15.6 µg/mL) and *E. coli* (MIC: 62.5 µg/mL) (Raj et al. 2012). The ayurvedic medicinal plant *Aegle marmelos* (L.) Corrêa produces xanthoarnol (**35**) active against *E. faecalis* (MIC: 18.7 µg/mL) (Chakthong et al. 2012). Examples of antimycobacterial coumarins with two isoprene groups are dentatin (**36**), nor-dentatin (**37**), and clausenidine (**38**) in *Clausena excavata* Burm.f. (Sunthitikawinsakul et al. 2003).

#### O-Prenylated coumarins

They are common in the Rutaceae and Apiaceae families. Imperatonin (**39**) and marmin (**40**) from *A. marmelos* inhibited the growth of *M. tuberculosis* with IC_50_ values of 12.4 and 4.3 µg/mL, respectively (Chinchansure et al. 2015). Similarly, 8-geranyloxy-5,7-dimethyloxycoumarin (**41**) from *T. asiatica* was antimycobacterial (Phatchana and Yenjai 2014). In the Apiaceae family, *Ferula pseudalliacea* Rech.f. produces sanandajin (**42**) (*S. aureus*) (Dastan et al. 2016).

#### 3-Phenyl coumarins

These coumarins are found in the Fabaceae family. We can cite glycycoumarin (**43**) from *Glycyrrhiza glabra* L. with *S. mutans* (MIC: 12.5 µg/mL) (Demizu et al. 1988) and *Haemophilus influenzae* (MIC: 25 µg/mL), as well as glycyrin (**44**) against *H. influenzae* and *Moraxella catarrhalis* (MIC: 25 µg/mL) (Tanaka et al. 2001). Other examples are psoralidine (**45**) from *P. corylifolia* (Khatune et al. 2004) and indicanin B (**46**) from *Erythrina indica* Lam. the latter active against *S. aureus* (MIC: 9.7 µg/mL) and *M. smegmatis* (MIC: 18.5 µg/mL) (Waffo et al. 2000). Wedelolactone (**47**) from *Eclipta alba* (L.) Hassk. (Asteraceae, campanulids) inhibited the growth of *S. aureus*, *Salmonella typhimurium*, and *S. epidermidis* with MIC values of 20, 25, and 15 µg/mL, respectively (Dalal et al. 2010).

#### 4-Phenyl prenylated coumarins

These coumarins are prevalent in the fabids. Examples are calophyllolide (**48**), inophyllum C (**49**), and inophyllum E (**50**) from *Calophyllum inophyllum* L. (Calophyllaceae) (Yimdjo et al. 2004) as well as mesuol (**51**) (MDR-*S. aureus*, MIC: 2 µg/mL) from *Mesua ferrea* L. (Calophyllaceae) (Verotta et al. 2004). Cajanuslactone (**52**) from *Cajanus cajan* (L.) Huth (Fabaceae) was bactericidal for *S. aureus* (MIC/MBC: 31/125 µg/mL) (Kong et al. 2010).

#### Benzocoumarins

*Dendrobium nobile* Lindl. (Orchidaceae, monocots), is an orchid used in traditional Chinese medicine that produces dendrocoumarin (**53**) which inhibited *S. aureus*, *E. coli*, *Micrococcus tetragenus*, *Kocuria rhizophila*, and *B. cereus* with MIC values of 2.5, 0.6, 5, 5 and 2.5 µg/mL, respectively (Zhou et al. 2018). From this orchid, itolide A (**54**) was active against *S. aureus*, *E. coli*, *M. tetragenus*, *K. rhizophila*, and *B. cereus* with MIC values of 2.5, 1.2, 5, 10, and 1.2 µg/mL, respectively (Zhou et al. 2018).

#### Isocoumarins

8-Hydroxy-6-methoxy-3-pentylisocoumarin (**55**) from *Xylosma longifolia* Clos (Salicaceae, fabids) (*M. tuberculosis*: 40.5 µg/mL) (Truong et al. 2011).

### Stilbenes

The condensation of one hydroxycinnamic acid unit with three malonyl-CoA units and decarboxylation gives rise to stilbenes (Abe 2020; Valletta et al. 2021). These are generally weakly antibacterial, but their spectrum of activity is broad (Mattio et al. 2020) (Figure 4). Resveratrol (**56**) from *Cassia grandis* L.f. (Fabaceae) was bactericidal against *S. aureus* (MIC: 125/125 µg/mL), *E. coli* (MIC: 50/125 µg/mL) (Kusumaningtyas et al. 2020), *S. mutans* (50/50 µg/mL), and *S. sanguis* (50/100 µg/mL) (Yim et al. 2010). Resveratrol (**56**) was bacteriostatic for a panel of Gram-positive bacteria (Paulo et al. 2010). Hydroxylation of resveratrol (**56**) at carbon 3′ forms piceatannol (**57**), which was bactericidal for *S. aureus* (MIC/MBC: 125/125 µg/mL) and *E. coli* (MIC/MBC: 100/125 µg/mL) (Kusumaningtyas et al. 2020). Methoxylation of resveratrol (**56**) at positions 3 and 5 gives rise to pterostilbene (**58**), which was bactericidal for *B. cereus* (MIC: 25 µg/mL) (Shih et al. 2021). Smiglastilbene (**59**) from *Smilax glabra* Roxb. (Smilacaceae, monocots) was weakly active against Gram-positive bacteria (Xu et al. 2013). Rhaponticin (**60**) from *Rheum rhaponticum* L. (Polygonaceae, malvids) was weakly bactericidal against *M. tuberculosis* (MIC/MBC: 128/256 µg/mL) (Smolarz et al. 2013). The methoxylation of stilbenes increases their antibacterial strength. This is observable with 3-hydroxy-5-methoxystilbene (**61**) from *P. glabrum* (*M. tuberculosis*, MIC: 3.3 µg/mL) (Said et al. 2015). An increase in activity is also observed when stilbenes are prenylated as in the case of cajanin stilbene acid (**62**) from *C. cajan* with *S. epidermidis* (MIC/MBC: 13/100 µg/mL), *S. aureus* (MIC/MBC: 25/105 µg/mL), and *Bacillus subtilis* (MIC/MBC: 25/250 µg/mL) (Kong et al. 2010). Cajanin stilbene acid (**62**) was active against VRE with a MIC as low as 1 µg/mL. Interestingly, intravenous administration of cajanin stilbene at a dose of 5 mg/kg per day for 7 days resulted in a 90% survival rate in rodents infected with VRE (Tan, Hua, et al. 2020).

#### Dihydrostilbenes

Reduction of the Δ^7,7′^ double bond of stilbenes forms antibacterial dihydrostilbenes in monocots. We can cite for instance dihydropinosylvine (**63**) from *Dioscorea batatas* Decne. (Dioscoreaceae, Monocots) (Takasugi et al. 1987) and the phytoalexin desmethylbatatasin IV (64) (*P. aeruginosa*, MIC: 10 µg/mL) (Fagboun et al. 1987; Adesanya et al. 1989). *Bletilla striata* (Thunb.) Rchb. F. (Orchidaceae) which is used in traditional Chinese medicine, produces batatasin III (**65**) as well as an unusual type of dihydrostilbenes with ethylbenzene groups, namely bulbocol (**66**), shanciguol (**67**), and shancigusine B (68) active against *S. aureus* with MIC values of 9, 7, and 3 µg/mL, respectively (Jiang et al. 2019).

#### Oligostilbenes

Plants of the family Vitaceae (Rosids) and Dipterocarpaceae (Malvids) use resveratrol (**56**) to construct oligostilbenes active against Gram-positive bacteria. ɛ-Viniferin (**69**) from *Vitis amurensis* Rup. (Vitaceae) was bacteriostatic for MRSA (MIC: 50 µg/mL) (Basri et al. 2014) and bactericidal for *S. mutans* and *S. sanguis* with MIC/MBC values of 25/50 and 50/50 µg/mL, respectively (Yim et al. 2010). Other examples include balanocarpol (**70**), vaticanol B (**71**) (Sahidin et al. 2017), and flexuosol A (**72**) from *Dryobalanops lanceolata* Burck (Dipterocarpaceae) (Wibowo et al. 2011). We can also cite dehydro-δ-viniferine (**73**) from *Dryobalanops rappa* Becc (Wibowo et al. 2022) bacteriostatic for *S. aureus* (MIC/MBC: 2/16 µg/mL) (Mattio et al. 2019).

#### Miscellaneous

The prenylated stilbene derivative lakoochin A (**74**) from *Artocarpus lakoocha* Wall. ex Roxb. (Moraceae) inhibited the growth of *M. tuberculosis* (MIC: 12.5 µg/mL) (Puntumchai et al. 2004).

### Diarylheptanoids

**Figure 5.**
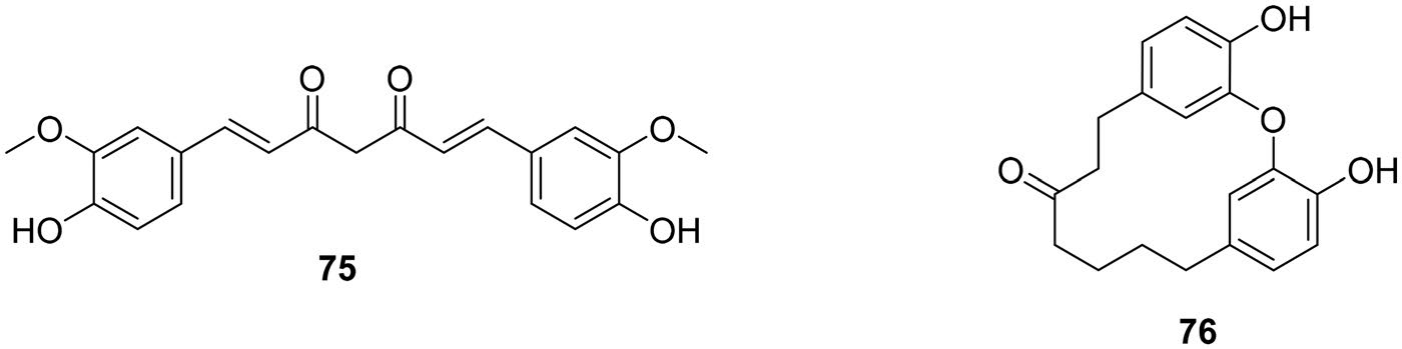
Diarylheptanoids.

The addition of two units of *p*-coumaric acid (**2**) or ferulic acid (**14**) with one malonyl-CoA unit forms antibacterial diarylheptanoids (Abe 2020) such as curcumin (**75**) in *Curcuma longa* L. (Zingiberaceae) (Gunes et al. 2016). The cyclic diaryheptanoid engelhardione (**76**) from *Engelhardia roxburghiana* Wall. (Juglandaceae, fabids) inhibited the growth of *M. tuberculosis* with the MIC value of 2 µg/mL (Lin et al. 2005) (Figure 5).

### Lignans

**Figure 6.**
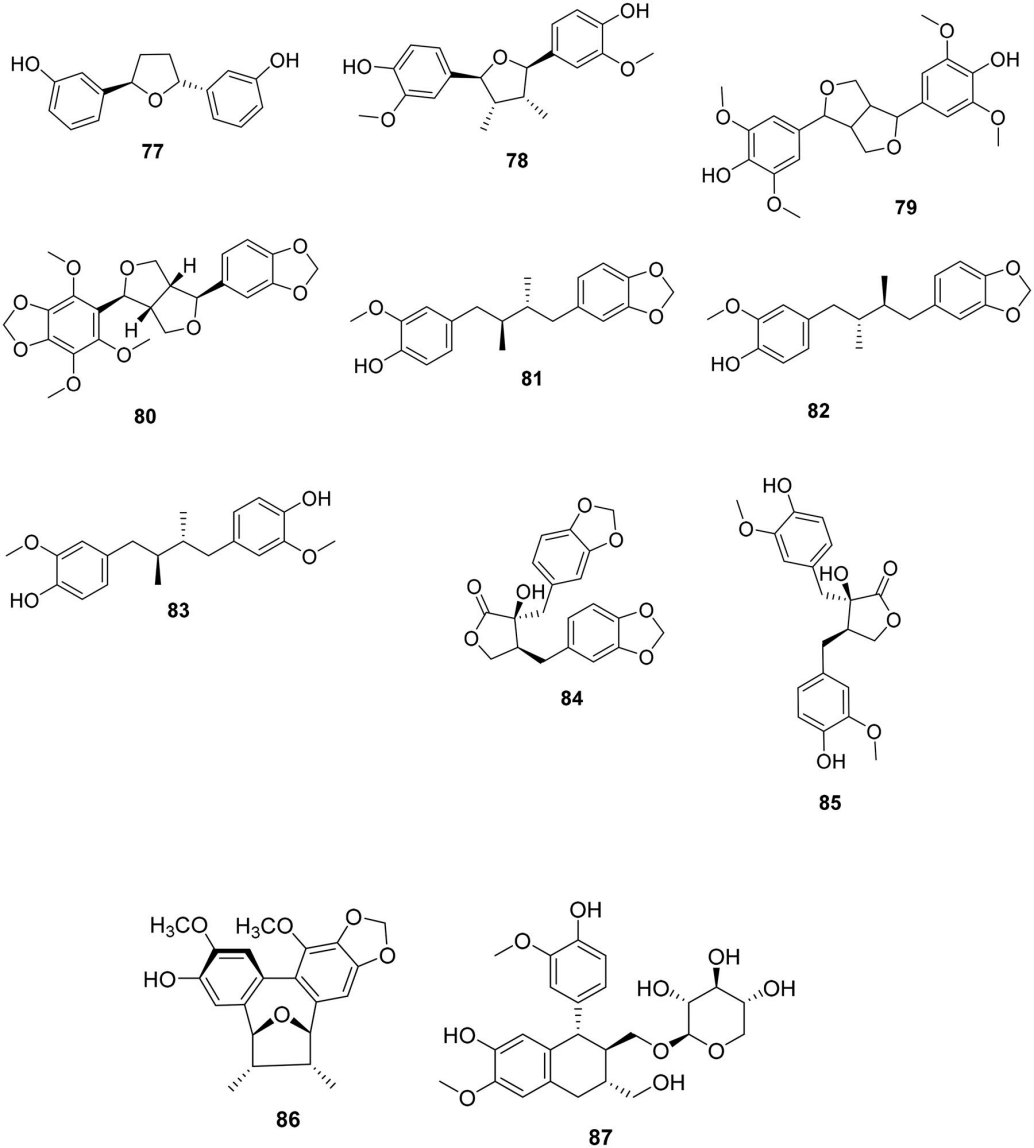
Lignans.

These phenolic compounds come from the coupling of two phenylpropanoid units between carbons 8 and 8′ (Lewis and Davin 1999; Satake et al. 2015) (Figure 6).

#### Tetrahydrofurans

Ammaniol (**77**) from *Ammannia multiflora* Roxb. (Lythraceae, malvid) inhibited the growth of *M. tuberculosis* (MIC: 25 µg/mL) (Upadhyay et al. 2012). Nectandrine B (**78**) from *Myristica fragrans* Houtt. (Myristicaceae, magnoliids) was active against *Pseudomonas syringae* (IC_50_: 63 µg/mL) (Cho et al. 2007).

#### Furanofurans

In upper Angiosperms, examples are syringaresinol (**79**) from *Canthium horridum* Bl. (Rubiaceae, lamiids) (Yang et al. 2010) and ecbolin A (**80**) in *Ecbolium viride* (Forssk.) Alston (Acanthaceae, lamiids) (*S. aureus*, MIC: 7.8 µg/mL) (Cecilia et al. 2012).

#### Dibenzylbutanes

Macelignan (**81**) from *M. fragrans* was bactericidal against *S. mutans* (MIC/MBC: 3.9/7.8 µg/mL) (Chung et al. 2006). From this plant, *erythro*-austrobailignan-6 (**82**) and *meso*-dihydroguaiaretic acid (**83**) inhibited the growth of *Agrobacterium tumefaciens* with IC_50_ values of 17 and 23 µg/mL, respectively (Cho et al. 2007). *erythro*-Austrobailignan-6 (**82**) was active against MRSA and MDR-*M. tuberculosis* with the MIC values of 50 µg/mL, respectively (Reyes-Melo et al. 2017).

#### Dibenzylbutyrolactones

Examples are meridinol (**84**) in *Lasia spinosa* (L.) Thwaites (Araceae, monocots) (Hasan et al. 2011) as well as (−)-nortrachelogenin (**85**) from *Patrinia scabisifolia* Link (Caprifoliaceae, campanulids), the latter being active against *E. coli* O157:H7 (Lee, Ji, et al. 2016).

#### Dibenzocyclooctadienes

Manglisin B (**86**) from *Manglietiastrum sinicum* Y.W. Law (Magnoliaceae, magnoliids) (Ding et al. 2014; Qiang et al. 2022).

#### Aryltetralins

Schizandriside (**87**) from *Acer truncatum* Bunge (Sapindaceae, malvids) developed an inhibition zone against *S. aureus* (2 µg/disc) (Dong et al. 2006; Shen et al. 2022).

### Neolignans

**Figure 7.**
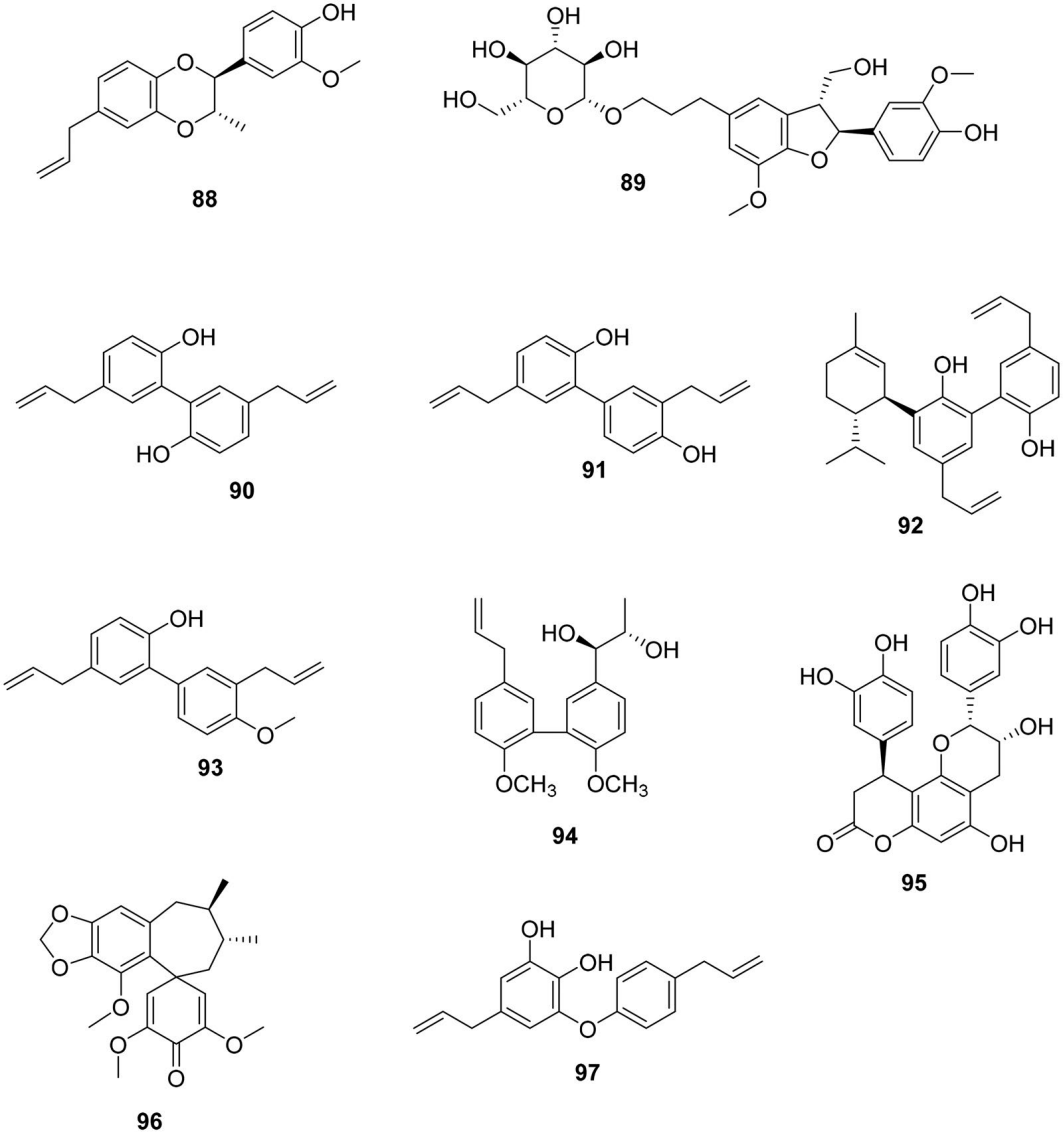
Neolignans.

These lignans come from the coupling of two phenylpropanoid units between carbons other than 8 and 8′ (Teponno et al. 2016) (Figure 7).

#### Benzodioxanes

Melaleucin A (**88**) from *Melaleuca bracteata* F. Muell. (Myrtaceae, malvids) inhibited the growth of MRSA (MIC: 8 µg/mL) (Li et al. 2017).

#### Dihydrobenzofuran lignans

The 8-5′ coupling of two phenylpropanoid units (Wang, Wang, et al. 2022) yields dihydrobenzofuran lignans which have broad-spectrum antibacterial effects. An example is glochidioboside (**89**) in the genus *Glochidion* J.R. Forst. & G. Forst. (Phyllanthaceae, fabids) which inhibited the growth of *E. coli* O157:H7 (Lee, Woo, et al. 2015).

#### Biphenyl lignans

The coupling of two phenylpropanoid units between carbons 3 and 3′ forms biphenyl lignans mostly found in the Magnoliaceae family. Examples are magnolol (**90**) and honokiol (**91**) in *Magnolia officinalis* Rehder & E.H. Wilson active against Gram-positive bacteria (Ho et al. 2001). Honokiol (**91**) was bactericidal against *S. aureus*, *B. subtilis*, *Propionibacterium acnes*, and *Propionibacterium granulosum* with MIC/MBC values of 13.1/26.6, 8.2/16.7, 4.1/16.7, and 8.2/16.7 µg/mL, respectively (Kim et al. 2010) as well as MRSA (MIC: 12.5 µg/mL) (Syu et al. 2004). From *M. officinalis,* piperitylmagnolol (**92**) gave MIC values of 12.5, 6.2, 6.2, and 6.2 µg/mL with *S. aureus*, MRSA, *E. faecalis*, and VRE, respectively, and was bactericidal against VRE (Syu et al. 2004). Other examples are 3,5′-diallyl-2′-hydroxy-4-methoxybiphenyl (**9**3) from *Magnolia grandiflora* L. (Clark et al. 1981). In the family Moraceae, an example is (7′*R*,8′*S*)-4,4′-dimethoxy-strebluslignanol (**94**) from *Streblus asper* Lour. (Moraceae) (Nie et al. 2016).

#### Miscellaneous lignans

These antibacterial lignans are abundant in basal Angiosperms and include cinchonain Ib (**95**) from *S. glabra* (Xu et al. 2013), manglisin A (**96**) from *M. sinicum* (Ding et al. 2014), and the biphenyl ether lignan obovatol (**97**) from *Magnolia obovata* Aiton (Magnoliaceae) (Ito et al. 1982).

### Hydroxybenzoic acid derivatives

**Figure 8.**
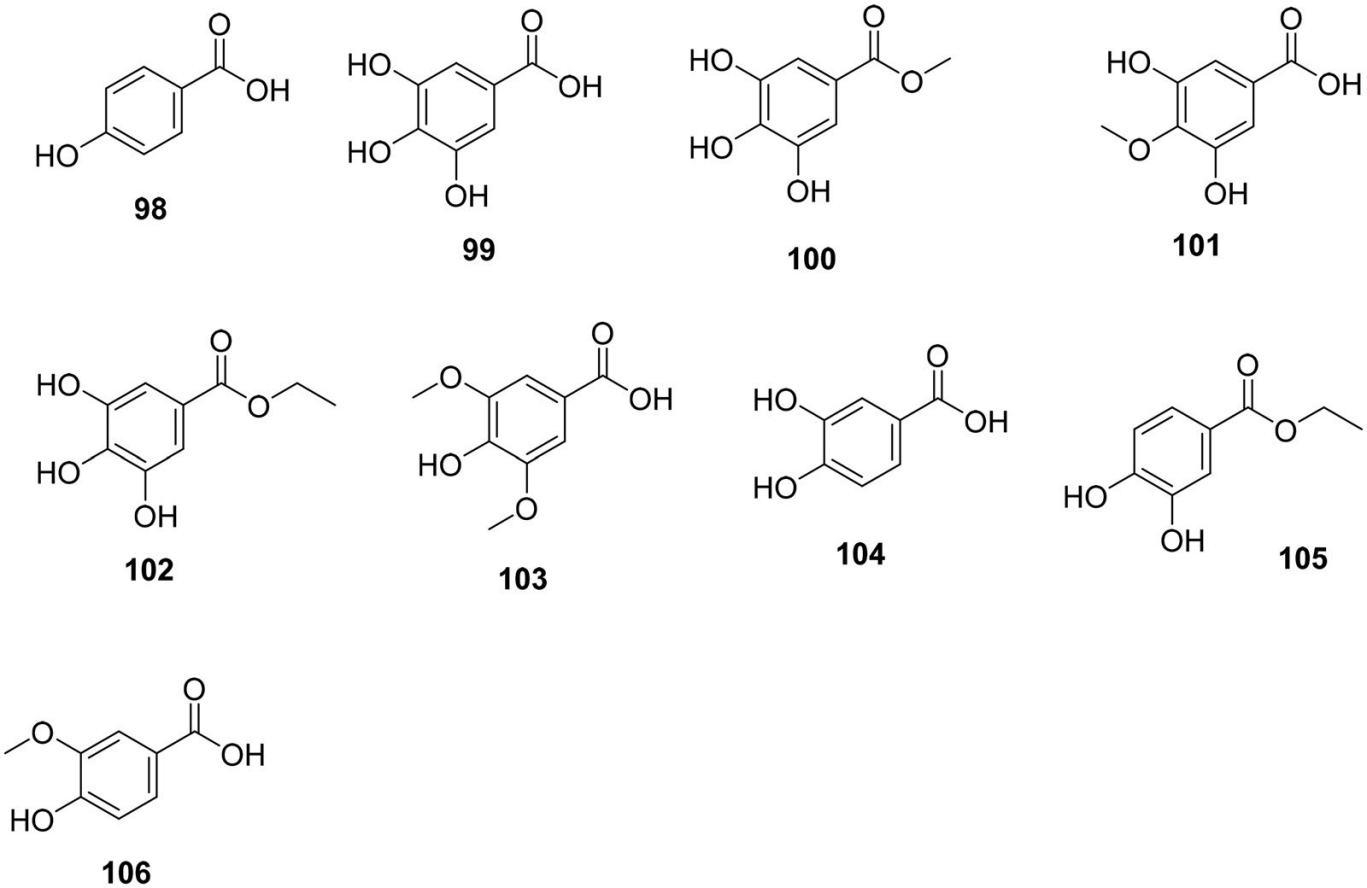
Hydroxybenzoic acid derivatives.

They derive from the shikimate pathway (Ossipov et al. 2003) and have a broad spectrum of antibacterial activity (Figure 8). One of the simplest examples is 4-hydroxybenzoic acid (**98**) from *Oryza sativa* L. (Poaceae, monocots) (Cho et al. 1998). Further addition of hydroxyl groups at positions 3 and 5 forms gallic acid (**99**) active against *S.* aureus, MRSA (MIC: 64 µg/mL), *M. tuberculosis* (MIC: 66.6 µg/mL) (Deng et al. 2013), and *S. epidermidis* (Adesina et al. 2000). Methyl gallate (**100**) from *Rhus chinensis* Mill. (Anacardiaceae, malvids) was active against *P. aeruginosa* and *E. coli* with MIC values of 12.5 and 25 µg/mL, respectively (Saxena et al. 1994) as well as against *S. aureus* (MIC: 7.8 µg/mL) (Xu et al. 2015), *Vibrio cholerae* (MIC: 30 µg/mL) (Sánchez et al. 2013), *Salmonella typhi* (MIC: 3.9 µg/mL) (Choi et al. 2014), and *M. tuberculosis* (MIC: 50 µg/mL) (Hernández-García et al. 2019). Methyl gallate (**100**) was weakly bactericidal against *Shigella dysenteriae* (MIC/MBC: 128/256, µg/mL) (Acharyya et al. 2015) and *K. pneumoniae* (MIC/MBC: 100/300 µg/mL (Li, Lin, et al. 2016). From *Rhus glabra* L. (Anacardiaceae), 4-methoxy-3,5-dihydroxybenzoic acid (**101**) was broadly antibacterial (Saxena et al. 1994).

In the family Saxifragaceae (core eudicots), *Saxifraga melanocentra* Franch. produces ethyl gallate (**102**) which is bactericidal against *K. pneumoniae* (Li, Lin, et al. 2016). Methoxylation of gallic acid (**99**) at positions 3 and 5 forms syringic acid (**103**) in *Ardisia elliptica* Thunb. (Myrsinaceae, asterids) active against *S. typhimurium* (MIC: 62.5 µg/mL) (Phadungkit and Luanratana 2006).

Protocatechuic acid (**104**) (Metsämuuronen and Sirén 2019) from *Arbutus unedo* L. (Ericaceae, asterids) was active against *A. baumannii* (Liu et al. 2005), while protocatechuic acid ester (**105**) from *Arachis hypogaea* L. (Fabaceae) was weakly active against *S. aureus* (Miklasińska et al. 2015). Protocatechuic acid (**104**) is the precursor of vanillic acid (**106**) (Metsämuuronen and Sirén 2019) which was active against *M. tuberculosis* (MIC: 83.3 µg/mL) (Deng et al. 2013).

### Miscellaneous simple phenolic compounds

**Figure 9.**
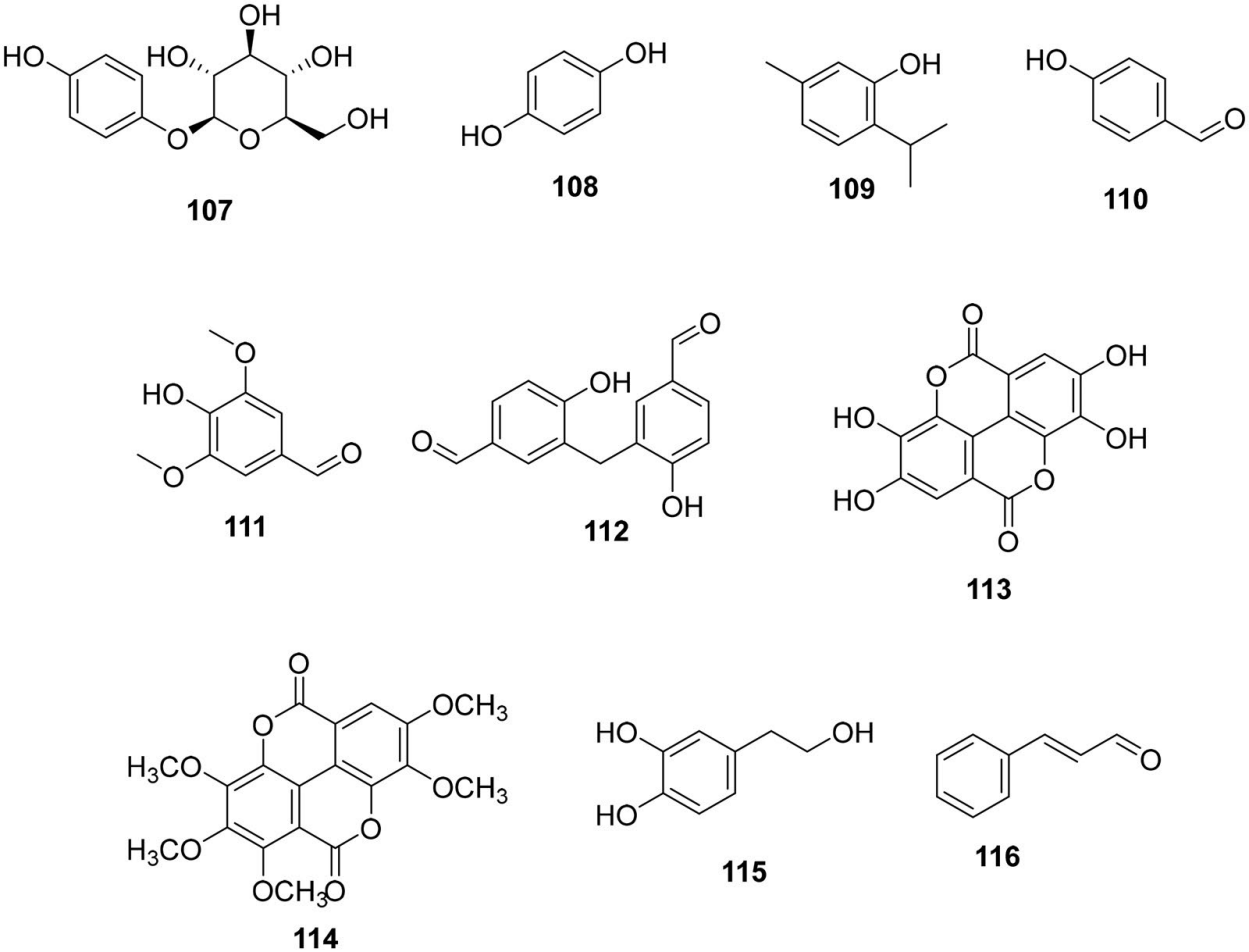
Miscellaneous simple phenolic compounds.

Examples are arbutin (**107**) and hydroquinone (**108**) from *A. unedo* (Jurica et al. 2017) (Figure 9), thymol (**109**) from *Thymus vulgaris* L. (Lamiaceae) (*E. coli*, MIC: 8 µg/mL) (Xu et al. 2008), 4-hydroxybenzaldehyde (**110**) from *Alpinia conchigera* Griff. (Zingiberaceae) (Aziz et al. 2012), syringaldehyde (**111**) from *Juglans regia* L. (Juglandaceae) (Colaric et al. 2005) (*S. mutans*, MIC/MBC: 62.5/62.5 µg/mL) (Meerungrueang and Panichayupakaranant 2014), 3,3′-methylene-bis(4-hydroxybenzaldehyde) (**112**) from *S. asper* (*B. subtilis*, MIC: 27 µg/mL) (Nie et al. 2016), ellagic acid (**113**) (Ghudhaib et al. 2010), 3,3′,4,4′,5′-pentamethylcoruleoellagic acid (**114**) from *Rhodamnia dumetorum* (DC.) Merr. and L.M Perry (*Haemophilus influenza*, MIC: 9.3 µg/mL) (Lakornwong et al. 2018; Munvera et al. 2020), hydroxytyrosol (**115**) from *S. cuneata* (*S. aureus*, MIC: 2 µg/mL) (Zeng et al. 2015), and cinnamaldehyde (**116**) in *Cinnamomum cassia* (L.) J. Presl (Lauraceae) (Firmino et al. 2018).

### Benzoquinones

**Figure 10.**
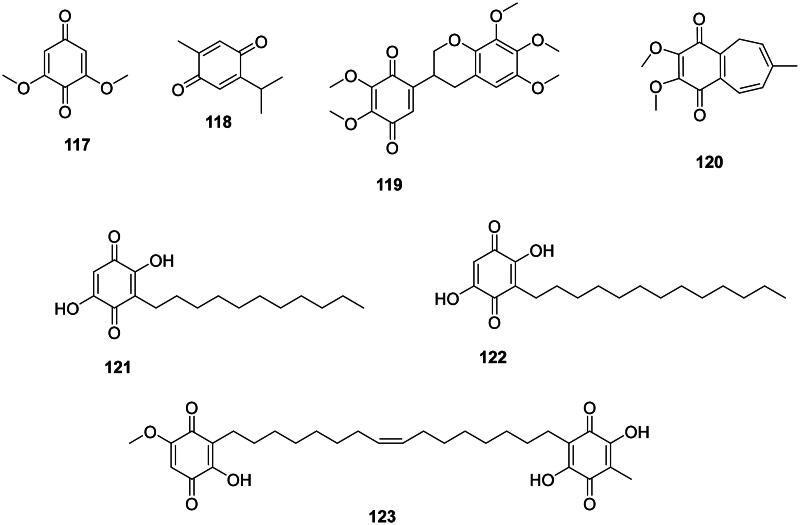
Benzoquinones.

Phenolic compounds in this group are generally strongly antibacterial (Figure 10). Examples are 2,6-dimethoxy-1,4-benzoquinone (**117**) from *Ficus foveolata* Pittier (Moraceae) bactericidal for *S. mutans* (MIC/MBC: 7.8/7.8 µg/mL) (Nishina et al. 1991; Meerungrueang and Panichayupakaranant 2014) and thymoquinone (**118**) from *Nigella sativa* L. (Ranunculaceae, eudicots) (Dey et al. 2014) bactericidal for *Listeria monocytogenes* (MIC: 8/8 µg/mL) and *S. aureus* (MIC/MBC 8/16 µg/mL) (Chaieb et al. 2011) and active against *E. coli* (Cetin-Karaca and Newman 2015). Another example is abruquinone B (**119**) from *Abrus precatorius* L. (Fabaceae) (*M. tuberculosis*, MIC: 12.5 µg/mL) (Limmatvapirat et al. 2004). Pulsaquinone (**120**) from *Pulsatilla koreana* (Yabe ex Nakai) Nakai ex T. Mori (Ranunculaceae) was active against *P. acnes*, *B. subtilis*, *S. aureus*, *S. mutans*, *P. aeruginosa*, and *S. sonnei* with the MIC values of 2, 2.7, 2, 2, 3.3, and 2 µg/mL, respectively (Cho et al. 2009).

Plants of the Myrsinaceae family produce antibacterial benzoquinones substituted with long-chain alkyl groups, such as embelin (**121**) from *Embelia ribes* Burm.f. bactericidal for *S. aureus* (MIC/MBC: 20/75 µg/mL) (Chitra et al. 2003; Radhakrishnan et al. 2011), rapanone (**122**) in *Ardisia crenata* Sims (Podolak et al. 2021), and ardisiaquinone B (**123**) from *Ardisia sieboldii* Miq. (*Enterobacter aerogenes*, MIC: 16 µg/mL) (Ogawa and Shinsaku 1968; Omosa et al. 2016).

### 1,4-Naphthoquinones

#### Simple 1,4-naphthoquinones

**Figure 11.**
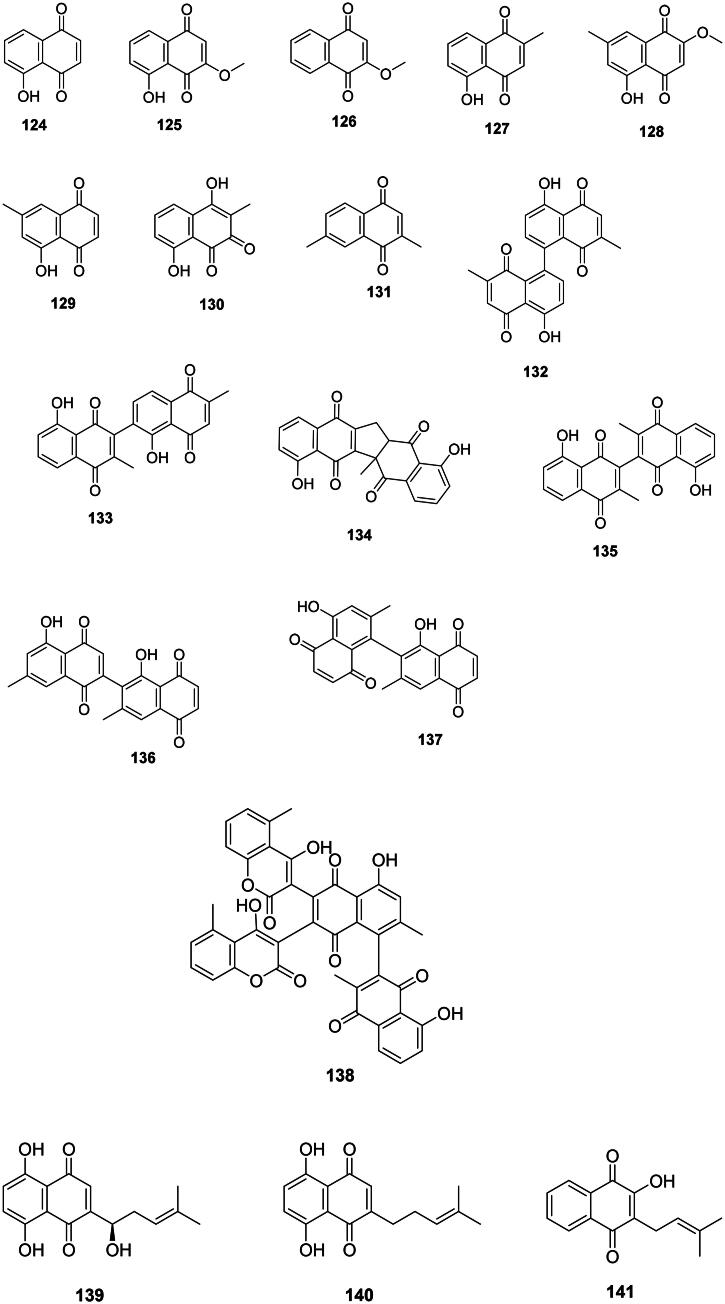
1,4-Naphthoquinones.

Simple 1,4-naphthoquinones originate from the polyketide or shikimate pathways (Widhalm and Rhodes 2016). These are among the most potent known antibacterial compounds found in Angiosperms (Figure 11). An example is juglone (**124**) (Zmantar et al. 2016), bacteriostatic against *Streptococcus pyogenes* (MIC/MBC: 1.5/100 µg/mL) (Macé et al. 2017) and active against *M. smegmatis* (MIC: 0.7 µg/mL) (Clark et al. 1990). 3-Methoxyjuglone (**125**) from *E. roxburghiana* was active against *M. tuberculosis* with a MIC as low as 0.2 µg/mL (Lin et al. 2005). 2-Methoxy-1,4-naphthoquinone (**126**) (lawsone methyl ether) from *Impatiens balsamina* L. (Balsaminaceae, asterids) inhibited the growth of *Aeromonas salmonicida* and MRSA with MIC values of 2 and 15.6 μg/mL, respectively (Yang et al. 2001).

The condensation of one acetyl-CoA unit with five malonyl-CoA units forms naphthoquinones with strong antibacterial effects in the order Ericales (asterids). An example is plumbagin (**127**) from *Diospyros kaki* Thunb. (Ebenaceae) (Lee and Lee 2008) active against *S. epidermidis* (MIC: 0.7 µg/mL) (Jeyachandran et al. 2009), *Neisseria gonorrhoae* (MIC: 19.5 µg/mL) (Kuete et al. 2009), *Gardnerella vaginalis* (Sobhani et al. 2018), MDR-*M. tuberculosis* (MIC/MBC: 0.2/1.5 µg/mL), and bactericidal for *Proteus vulgaris* (MIC/MBC: 16/16 µg/mL) (Dey et al. 2014). Plumbagin (**127**) was bactericidal for *M. smegmatis* and *M. tuberculosis* (MIC/MBC: 4.8/9.7 µg/mL) (Kuete et al. 2009). From *D. kaki*, 2-methoxy-7-methyl juglone (**128**) (Gu et al. 2004) inhibited the growth of *M. tuberculosis* with a MIC as low as 0.5 µg/mL (selectivity index: 30.2) (Mahapatra et al. 2007). *Diospyros maritima* Bl. (Ebenaceae) yields 7-methyljuglone (**129**) active against *M. tuberculosis* (MIC: 0.5 µg/mL) (Bapela et al. 2006) and bacteriostatic for *M. smegmatis* (MIC/MBC: 1.5/15.6 µg/mL) (McGaw et al. 2008). *D. maritima* produces droserone (**130**) which inhibited the growth of pan-resistant *Mycobacterium tuberculosis* (clinical isolate CIBIN 99) with the MIC value of 25 µg/mL) (Uc-Cachón et al. 2014). Chimaphilin (**131**) from *Monenes uniflora* L. (Ericaceae) was active against *S. aureus* (MIC: 25 µg/mL) (Saxena et al. 1996) and *M. tuberculosis* (IC_50_: 5.4 µg/mL) (Li et al. 2018).

#### 1,4-Naphthoquinones oligomers

These phenolic compounds are produced from the oxydative coupling of naphthoquinones in the Ebenaceae (Figure 11). Examples of dimers of plumbagin (**127**) are maritinone (**132**), chitranone (**133**), zeylanone (**134**), and 3,3′-biplumbagin (**135**) in *D. maritima* (Gu et al. 2004) which inhibited pan-resistant *M. tuberculosis* (clinical isolate CIBIN 99) with the MIC values of 3.1, 3.1, 12.5, and 3.1 µg/mL, respectively (Uc-Cachón et al. 2014). Of note, the selectivity indexes of maritinone (**132**) and 3,3′-biplumbagin (**135**) were 74.3 and 194.1, respectively (Uc-Cachón et al. 2014).

Diospyrin (**136**) formed from the coupling between a pair of 7-methyljuglone (**129**) is active against *Corynebacterium dyphtheriae* (MIC: 3.1 µg/mL) (Adeniyi et al. 2000) and bacteriostatic for *Mycobacterium bovis* (MIC/MBC: 1.7/39 µg/mL) (McGaw et al. 2008). Likewise, isodiospyrin (**137**) inhibited *Streptococcus pneumoniae* with a MIC value as low as 0.7 µg/mL (McGaw et al. 2008). An example of 1,4-naphthoquinone dimer coupled with coumarins is diospyrone (**138**) active against MDR-*K. pneumoniae* and MDR-*P. aeruginosa* (Kuete et al. 2009).

#### Prenylated 1,4-naphthoquinones

Lamiids and plants of the Boraginaceae family combine a 4-hydroxybenzoic acid unit with a geranyl group to form antibacterial prenylated 1,4-naphthoquinones with naphthazarin scaffolds (Rajbhandari et al. 2007). This is the case of shikonin (**139**) and deoxyshikonin (**140**) from *Lithospermum erythrorhizon* Siebold & Zucc. used in traditional Chinese medicine (Brigham et al. 1999). Shikonin (**139**) from *Arnebia euchroma* (Royle ex Benth.) I.M. Johnst. was bactericidal against MRSA (MIC/MBC: 6.2/12.5 µg/mL) (Shen et al. 2002). In the Bignoniaceae family, an example is lapachol (**141**) from *Oroxylum indicum* (L.) Kurz (Bignoniaceae) (Ali et al. 1998).

### Anthraquinones

#### Simple anthraquinones

**Figure 12.**
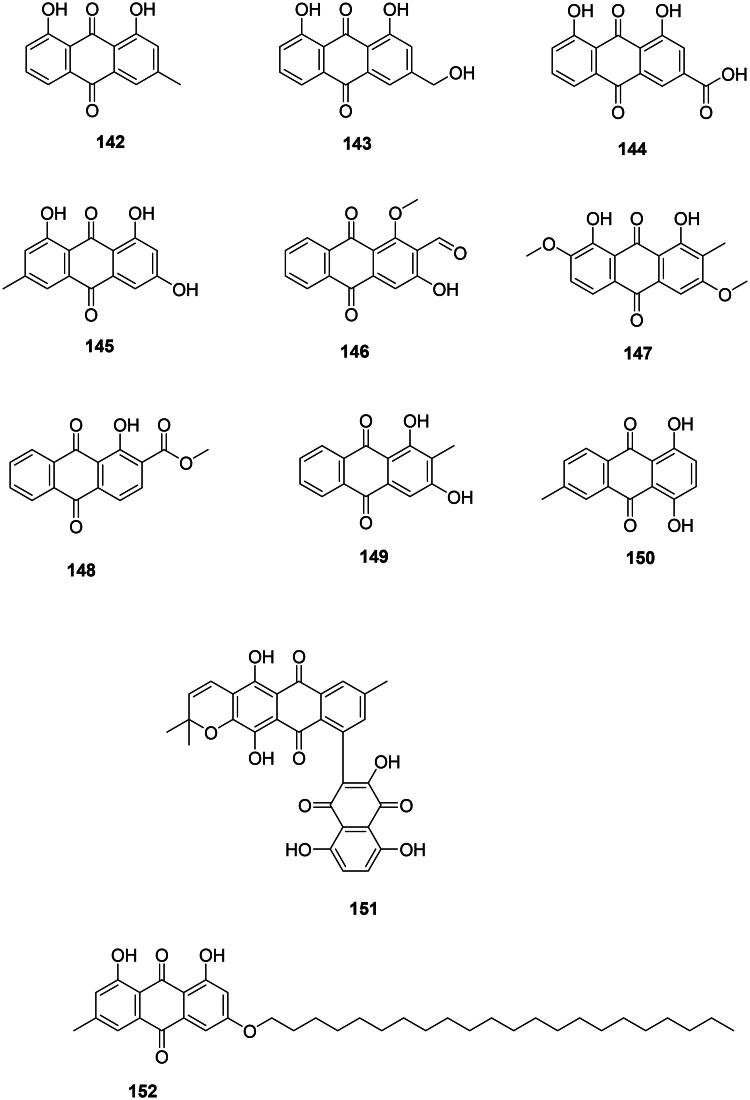
Anthraquinones.

Angiosperms produce antibacterial anthraquinones from the polyketide or shikimate pathways (Figure 12). For example, chrysophanol (**142**) from *R. rhaponticum*, formed by the addition of one acetyl-CoA unit to seven malonyl-CoA units, was bactericidal against *M. tuberculosis* (64/128 µg/mL) (Smolarz et al. 2013) and active against *S. epidermidis* (MIC: 31.2 µg/mL) (Coopoosamy and Magwa 2006a). The oxidation of chrysophanol (**142**) at carbon 3 forms aloe-emodin (**143**) in *R. rhaponticum* (Coopoosamy and Magwa 2006b; Alaadin et al. 2007; Lee, Kang, et al. 2010). Aloe-emodin (**143**) was active against MRSA (MIC: 2 µg/mL) (Hatano et al. 1999; Alaadin et al. 2007), *S. mutans* (MIC: 1.2 µg/mL) (Zheng et al. 2011), and bactericidal for *M. tuberculosis* (MIC/MBC: 64/128 µg/mL) (Smolarz et al. 2013). The oxidation of aloe-emodin (**143**) at carbon 3 forms rhein (**144**) in *Rheum officinale* Baill. (Polygonaceae) effective against MRSA (MIC: 15.6 µg/mL) (Joung et al. 2012), *Bacteroides fragilis* (MIC: 1.5 µg/mL) (Cyong et al. 1987), and *Porphyromonas gingivalis* (MIC: 2.5 µg/mL) (Azelmat et al. 2015).

Another example is emodin (**145**) from *Cassia alata* L. (Fabaceae) with methicillin-sensitive *S. aureus* (MIC: 25 µg/mL) (Joung et al. 2012), *S. aureus* (MIC: 8 µg/mL) (Yan et al. 2017), MRSA (MIC: 1.5 µg/mL) (Promgool et al. 2014), *M. tuberculosis* (MIC/MBC: 4/8 µg/mL) (Dey et al. 2014), *B. cereus* (MIC/MBC: 8/8 µg/mL) (Dey et al. 2014), and *Haemophilus parasuis* (MIC/MBC: 32/64 µg/mL) (Li, Song, et al. 2016).

In the family Rubiaceae, examples are damnacanthal (**146**) from *Morinda elliptica* (Hook.f.) Ridl. (*M. tuberculosis*, MIC: 13 μg/mL) (Pollo et al. 2020), 1,8-dihydroxy-2-methyl-3,7-dimethoxyanthraquinone (**147**) from *Morinda angustifolia* Roxb. (Xiang et al. 2008), 1-hydroxy-2-methoxycarbonyl-anthraquinone (**148**) from *Coptosapelta flavescens* Korth. (MRSA, MIC: 16 µg/mL) (Kongyen et al. 2014), and rubiadin (**149**) from *Rubia tinctoria* L. (*S. aureus*, MIC: 32 µg/mL) (Comini et al. 2011).

6-Methyl-1,4-dihydroxyanthraquinone (**150**) from *Tectona grandis* L.f. (Verbenaceae) was bacteriostatic for *Klebsiella aerogenes* (16/128 µg/mL) (Bitchagno et al. 2015).

#### Miscellaneous anthraquinones

*T. grandis* produces an unusual dimer of anthraquinone and naphthoquinone: tectograndone (**151**), bactericidal for *E. coli* (MIC/MBC: 32/128 µg/mL) (Bitchagno et al. 2015). Revandchinone-3 (**152**) from *Rheum emodi* Wall. (Polygonaceae) inhibited the growth of a broad spectrum of bacteria (Babu et al. 2003).

### Tannins

#### Proanthocyanidins

The coupling of two catechin and or epigallocatechin units forms proanthocyanidins (Figure 13). These phytoanticipins are weakly antibacterial but their spectrum of activity is broad. An example is cinnamtanin B1 (**153**) from *Vaccinium vitis-idaea* L. (Ericaceae) against *P. gingivalis* and *Prevotella intermedia* (MIC: 100 µg/mL) (Ho et al. 2001). Another illustration is (+)-epigallocatechin-(2β→*O*→7, 4β→8)-(+)-catechin (**154**) from *Quercus ilex* L (Fagaceae, fabids) (Karioti et al. 2011). The antibacterial spectrum of proanthocyanidin oligomers and polymers is limited to Gram-positive bacteria as in ZP-CT-A from *Zanthoxylum piperitum* DC (Rutaceae) (MRSA, MIC: 128 µg/mL) (Kusuda et al. 2006), theasinensin A. (**155**) and B (**156**) (MRSA, MIC: 64 µg/mL) (Hatano et al. 2003), and proanthocyanidins from *Diospyros kaki* L. (Ebenaceae) (Wang et al. 2020).

Figure 13. Tannins.

**Figure:**
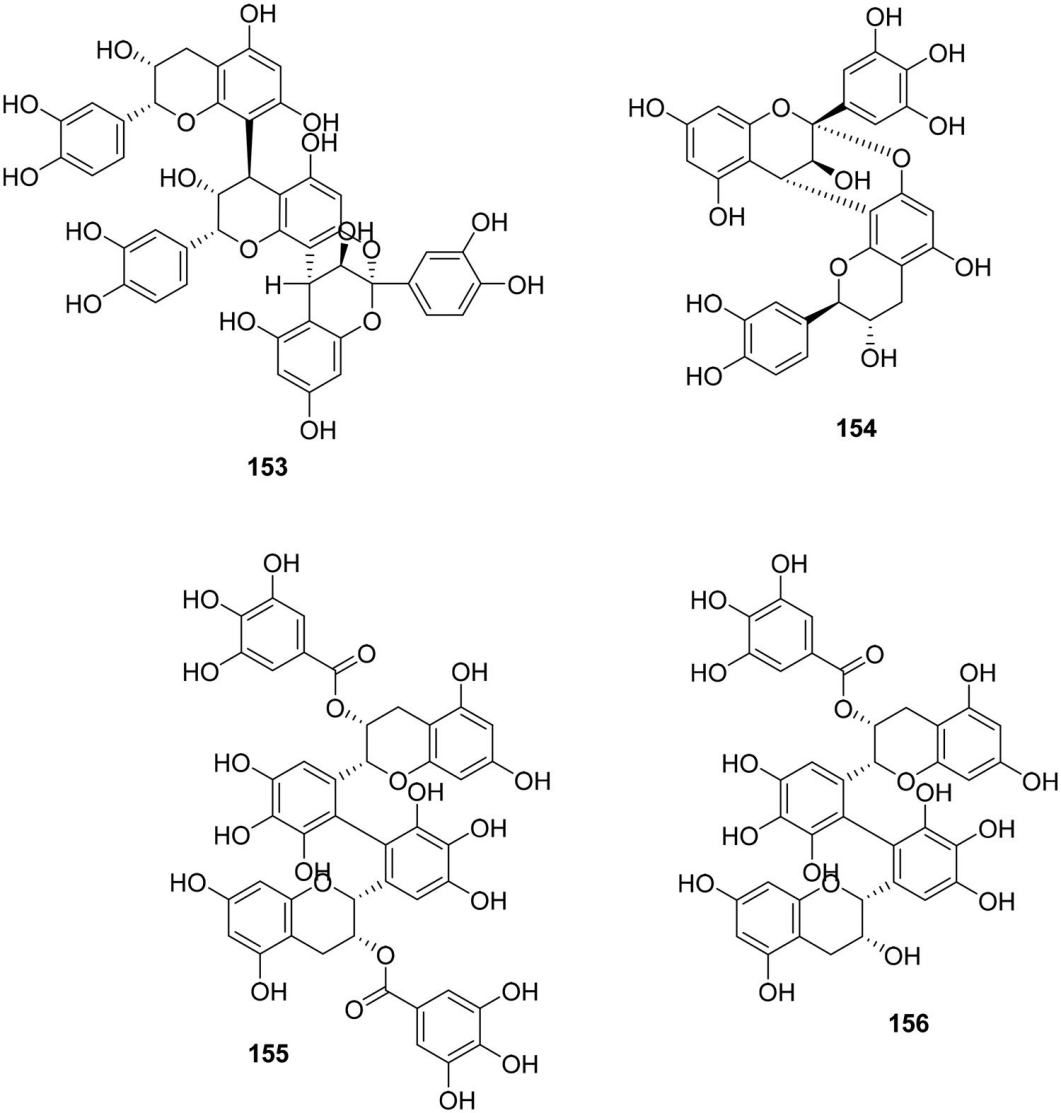

**Figure:**
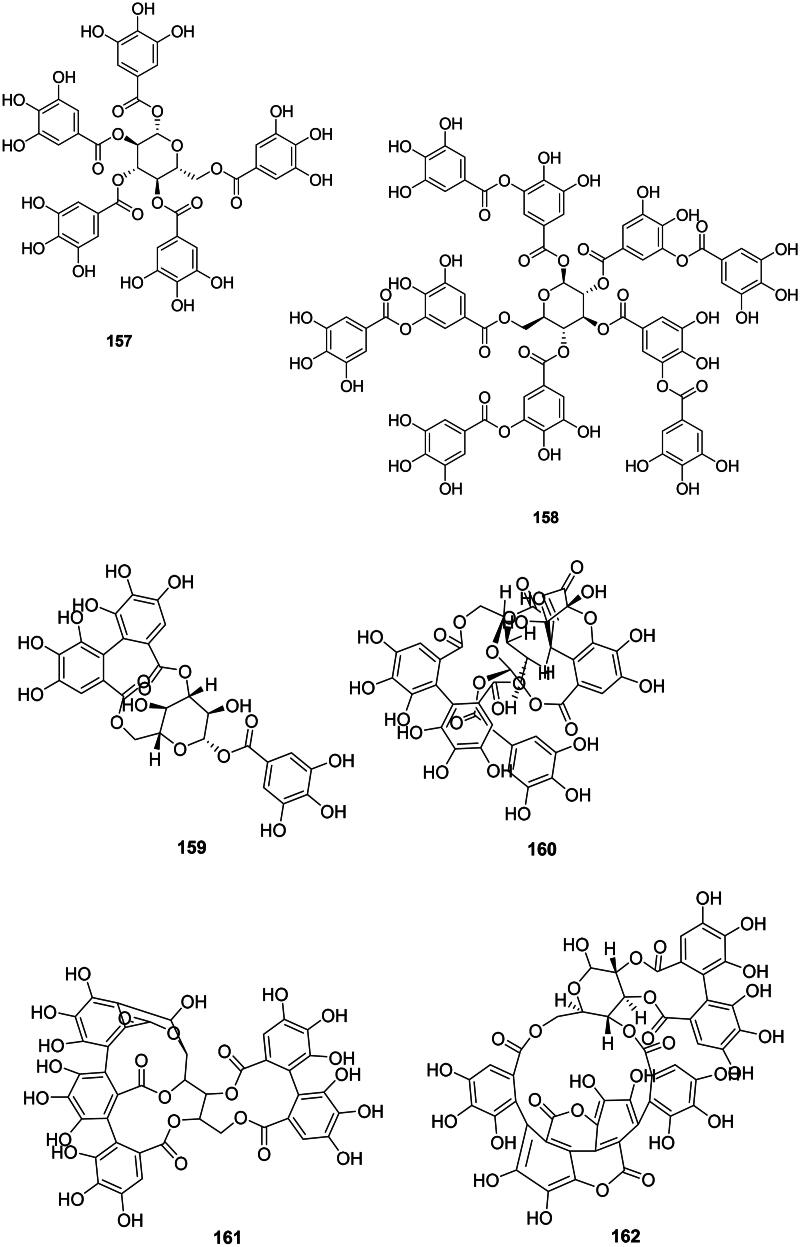

#### Gallotannins

The esterification of a glucose unit by several units of gallic acid (**99**) forms gallotannins (Ossipov et al. 2003). These phytoanticipins are weakly antibacterial and include 1,2,3,4,6-penta-*O*-galloyl-β-d-glucose (**157**) from *A. truncatum* (*S. aureus*, MIC: 60 µg/mL) (Zhang et al. 2008; Lin et al. 2011) and tannic acid (**158**) from *Alnus japonica* (Thunb.) Steud. (Betulaceae, fabids) (Wu et al. 2010).

#### Ellagitannins

Ellagitannins result from the coupling of two adjacent units of gallic acid (**99**) within gallotannins and generally have weak but broad-spectrum antibacterial activities (Figure 13) (Al-Harbi et al. 2017). These tannins are found in fabids and include, for example, corilagin (**159**) and geraniin (**160**) from *Acalypha wilkesiana* Müll. Arg. (Euphorbiaceae) active against *S. aureus* with MIC values of 50 and 25 µg/mL, respectively (Adesina et al. 2000). Corilagin (**159**) was active against *E. coli* (MIC: 62.5 µg/mL) (Li et al. 2013) and geraniin (**159**) with *Vibrio vulficus* (MIC: 25 µg/mL) (Taguri et al. 2006). In the malvids, examples are castalagin (**161**) from *Terminalia catappa* L. (Combretaceae) (*Clostridium perfringens*, MIC: 67 µg/mL) (Taguri et al. 2006) and punicalagin (**162**) (*S. aureus*, MIC: 250 µg/mL) from *Punica granatum* L. (Lythraceae) (Xu et al. 2017; Li et al. 2020). The oligomeric ellagitannin isorugosin A from *Liquidambar formosana* Hance (Altingiaceae, core eudicots) was active against MRSA (Shimozu et al. 2017).

### Miscellaneous phenolic compounds

#### Long-chain alkyl phenols

**Figure 14.**
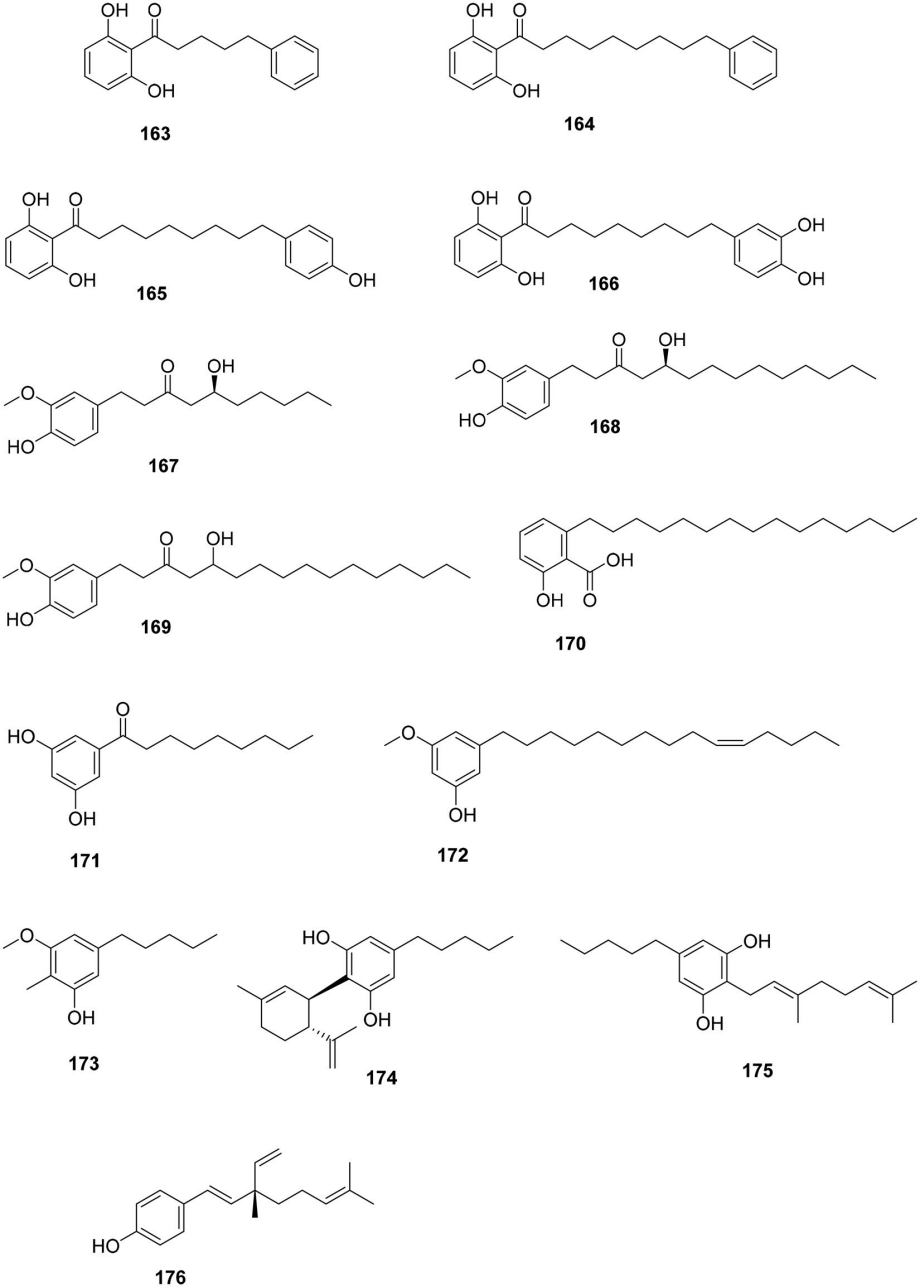
Long-chain alkyl phenols.

These phenolic compounds originate from the polyketide pathway and are strongly active against Gram-positive bacteria (Sampietro et al. 2013) (Figure 14). In the basal Angiosperms, magnoliids produce antibacterial alkylresorcinols, such as knerachelin B (**163**) from *Knema furfuracea* (Hook.f. and Thomson) Warb. (Myristicaceae) (*S. aureus*, MIC: 4 µg/mL) (Zahir et al. 1993), malabaricone A (**164**) (*S. aureu*s, MIC: 0.5 µg/mL, bactericidal, selectivity index ≥ 80), and malabaricone B (**165**) (MRSA, MIC: 0.5 µg/mL, selectivity index ≥ 80) (Sivadas et al. 2023). Malabaricone B (**165**) inhibited the growth of VRE and MRSA with MIC values as low as 1 µg/mL and was bactericidal against MRSA (Sivadas et al. 2023). *Myristica fragrans* produces malabaricone C (**166**) active against *S. aureus* (MIC: 4 µg/mL) (Orabi et al. 1991). In monocots, *Zingiber officinale* Roscoe (Zingiberaceae) produces antibacterial alkyl catechols, such as [6]-gingerol (**167**), [10]-gingerol (**168**), and [12]-gingerol (**169**) (Hiserodt et al. 1998; Park et al. 2008).

Anacardic acid (**170**) from *Anacardium occidentale* L. (Anacardiaceae) inhibited the growth of *S. mutans* and *P. acnes* with MIC values as low as 1.5 and 0.7 µg/mL, respectively (Kubo et al. 1993). Other long-chain alkyl phenols of this kind have been identified in *Semecarpus anacardium* L.f. (Anacardiaceae) (Sundaram et al. 2014).

In upper Angiosperms, *Ardisia cornudentata* Mez (Myrsinaceae) produces 1-(3,5-dihydroxyphenyl)nonan-1′-one (**171**), belamcandol (**172**), and 3-methoxy-2-methyl-5-pentylphenol (**173**) which inhibited the growth of *M. tuberculosis* with MIC values of 6, 33.8, and 2.5 µg/mL, respectively (Chang et al. 2011). *Cannabis sativa* L (Cannabaceae, fabids) produce cannabidiol (**174**) and cannabigerol (**175)** active against *S. aureus* with MIC values as low as 0.5 and 1 µg/mL, respectively (Appendino et al. 2008; Radwan et al. 2009). Cannabidiol (**174**) inhibited the growth of *E. faecium*, *M. catarrhalis*, *N. gonorrhoeae*, *Neisseria meningitidis*, *Legionella pneumophila*, and *A. baumannii* with MIC values of 0.5, 1, 1, 0.2, 1, and 64 µg/mL, respectively (Blaskovich et al. 2021). Cannabidiol (**174**) applied topically could treat MRSA-infected mice but was inactive when given orally (Blaskovich et al. 2021). *Aerva sanguinolenta* (L.) Bl. (Amaranthaceae) produces bakuchiol (**176**) active against *S. mutans* with a MIC as low as 0.9 µg/mL (Rao et al. 2012) as well as *Mycobacterium aurum* (MIC: 15. 9 µg/mL) (Newton et al. 2002).

#### Prenylated phloroglucinols

**Figure 15.**
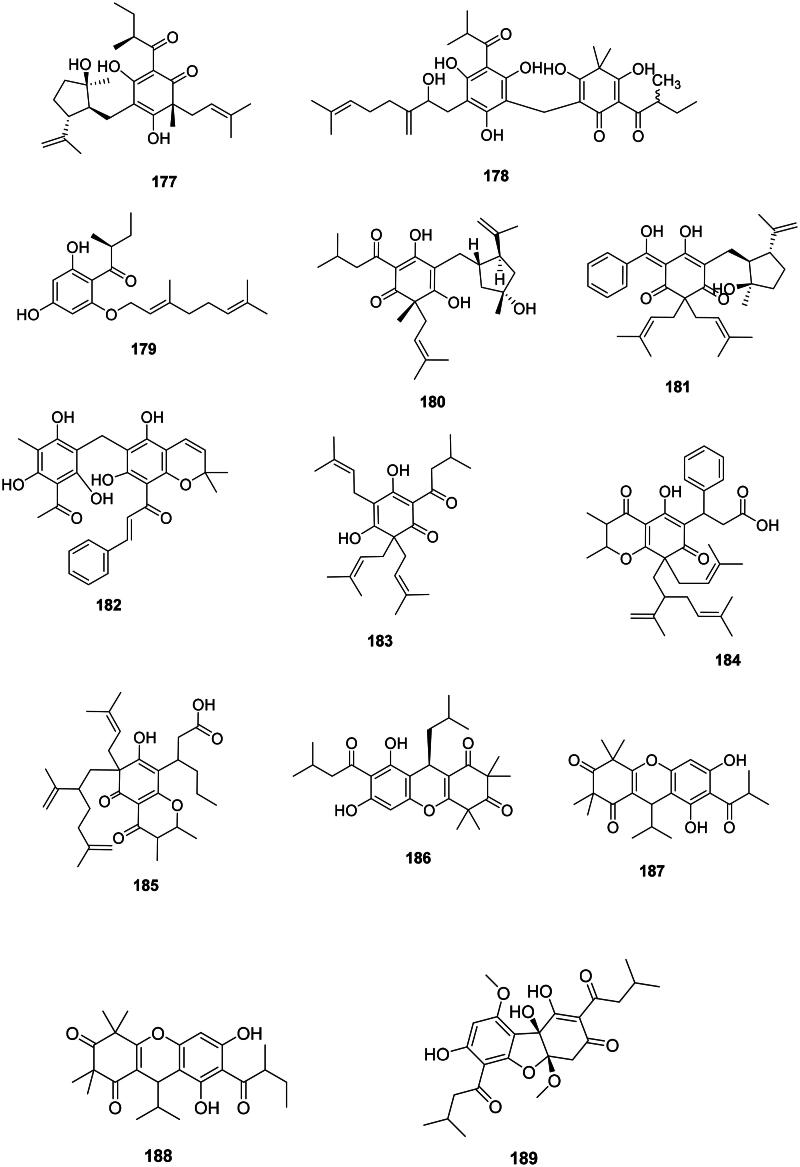
Prenylated phloroglucinols.

Fabids, and to a lesser extent malvids, produce prenylated phloroglucinols active against Gram-positive bacteria (Figure 15). In the family Hypericaceae, examples are chinesin I (**177**) from *Hypericum japonicum* Thunb. (*S. aureus*, MIC: 3.1 µg/mL) (Nagai and Tada 1987) as well as hyperjaponicol C (**178)** (Li et al. 2018), and olympicin A (**179**) from *Hypericum olympicum* L. (Shiu et al. 2012). Hypercalin A (**180**) and hypercalin B (**181**) from *Hypericum acmosepalum* N. Robson inhibited *S. aureus* (expressing NorA) with MIC values as low as 2 and 0.5 µg/mL, respectively (Osman et al. 2012).

Other examples are, in the fabids, rottlerin (**182**) from *Mallotus philippensis* (Lam.) Müll. Arg. (Euphorbiaceae) (Pandey et al. 2016), lupulone (**183**) from *Humulus lupulus* L. (Cannabaceae) (MRSA, MIC: 0.6 µg/mL) (Bocquet et al. 2019), as well as calophynic acid (**184**) and brasiliensic acid (**185**) from *C. inophyllum* (Yimdjo et al. 2004). In the family Myrtaceae, rhodomyrtone (**186**), isomyrtucommulone B (**187**), and myrciarone B (**188**) from *Myrciaria dubia* (Kunth) McVaugh inhibited *B. subtilis* with the MIC values of 0.7, 1.5, and 1.5 µg/mL, respectively (Kaneshima et al. 2017). Callistemenonone A (**189**) from *Callistemon viminalis* (Sol. ex Gaertn.) G. Don was bactericidal for *B. cereus* (MIC/MBC: 5/20 µg/mL) (Xiang et al. 2017).

#### Prenylated acetophenones

**Figure 16.**
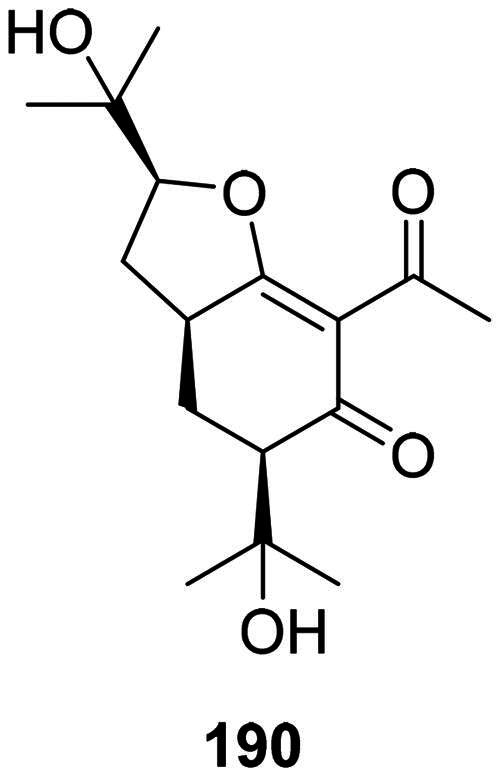
Prenylated acetophenones.

Meliviticine A (**190**) from *Melicope viticina* (Wall. ex Kurtz) T.G. Hartley (Rutaceae) inhibited the growth of MRSA, *S. typhi*, and *P. aeruginosa* (MIC: 50 µg/mL) (Li et al. 2019) (Figure 16).

#### Prenylated benzophenones

**Figure 17.**
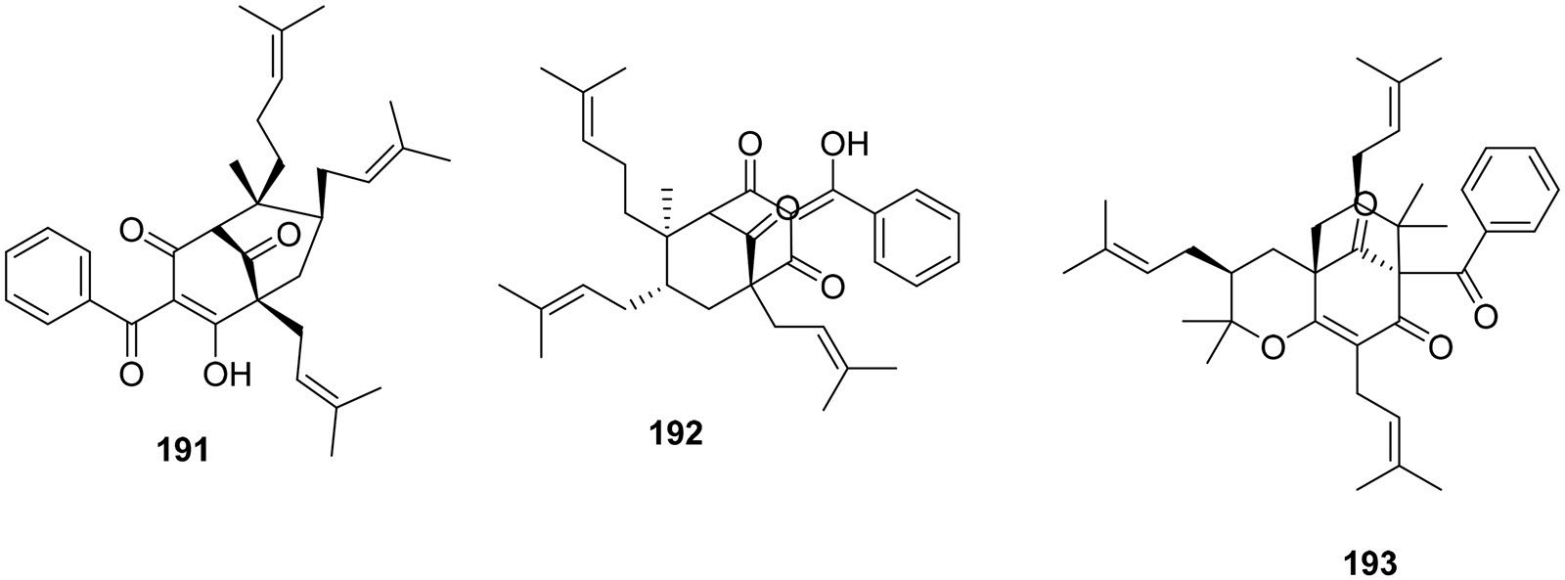
Prenylated benzophenones.

The addition of one benzoyl-CoA unit with three malonyl-CoA units and substitutions with dimethylallyl groups form prenylated benzophenones (Abe 2020). They are active against Gram-positive bacteria and are found in the family Clusiaceae (fabids) (Figure 17). Cowanone (**191**) from *Garcinia cowa* Roxb. inhibited the growth of MRSA with a MIC value as low as 0.5 µg/mL (Trisuwan and Ritthiwigrom 2012). *Garcinia multiflora* Champ. ex Benth. produces chamuangone (**192**) bactericidal for *S. pyogenes* (MIC/MBC: 7.8/31.2 µg/mL) (Sakunpak and Panichayupakaranant 2012) as well as garcimultiflorone A (**193**) (Chen et al. 2008).

#### Prenylated xanthones

Internal coupling of benzophenones forms a wide range of antibacterial prenylated xanthones in the Hypericaceae, Calophyllaceae, and Clusiaceae families (Figure 18). In the Hypericaceae family, examples are isocudraniaxanthone B (**194**), isojacareubine (**195**) bactericidal against MRSA (SCCmec III) (MIC/MBC: 4/16 µg/mL) (Zuo et al. 2012), and cochinchinone A (**196**) (*P. aeruginosa*, MIC: 4.7 µg/mL) from *Cratoxylum cochinchinense* (Lour.) Bl. (Boonnak et al. 2009). Other examples are gerontoxanthone I (**197**) and 9-hydroxycalabaxanthone (**198**) from *Cratoxylum formosum* (Jack) Benth. & Hook.f. ex Dyer (*S. typhi*, MIC: 1.1 µg/mL) (Boonsri et al. 2006). Caloxanthone A (**199**) from *C. inophyllum* was active against *S. aureus* (Yimdjo et al. 2004).

Figure 18. Prenylated xanthones.

**Figure:**
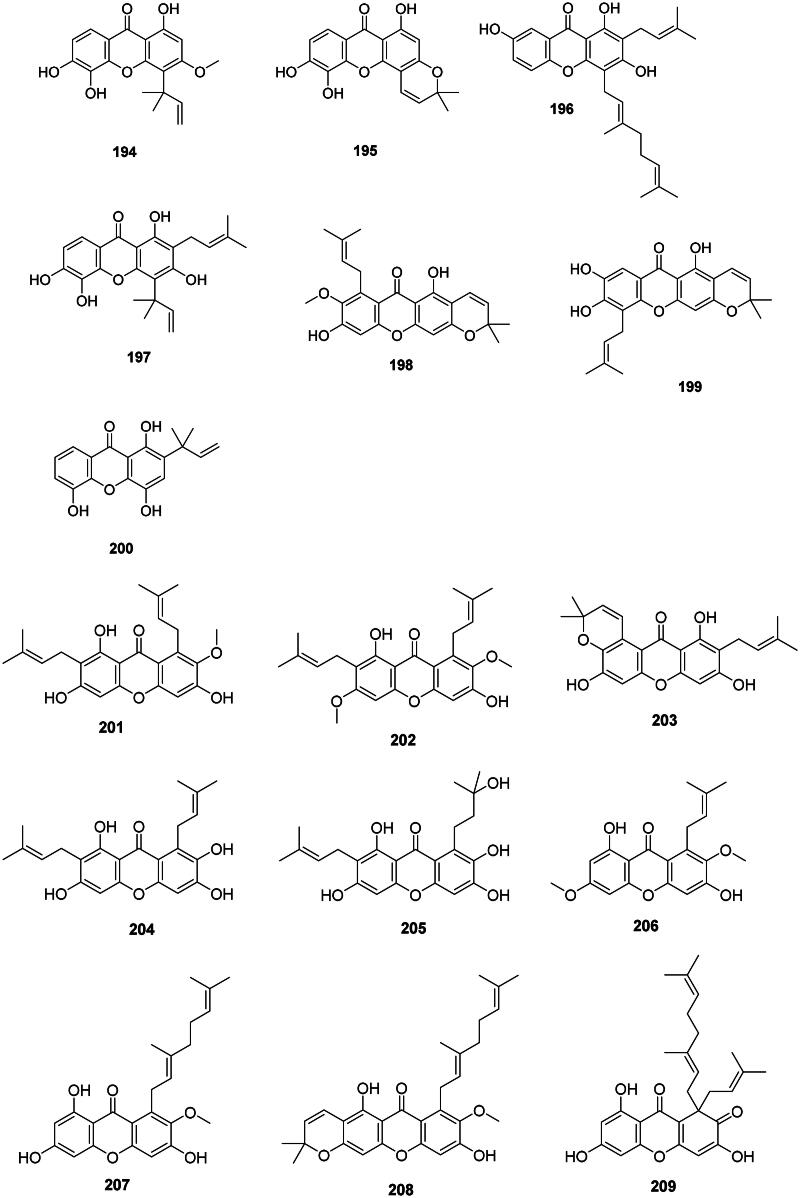

**Figure:**
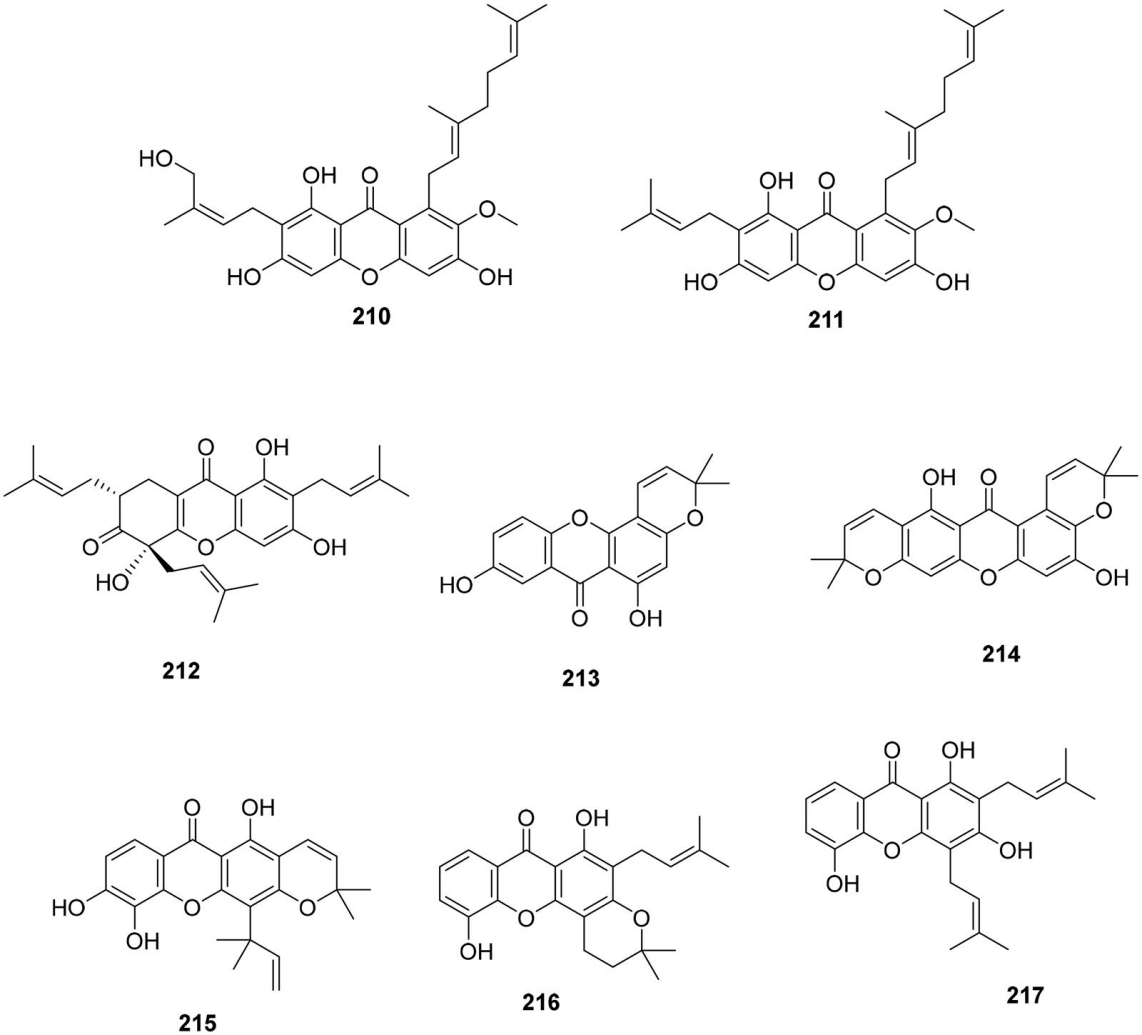

**Figure:**
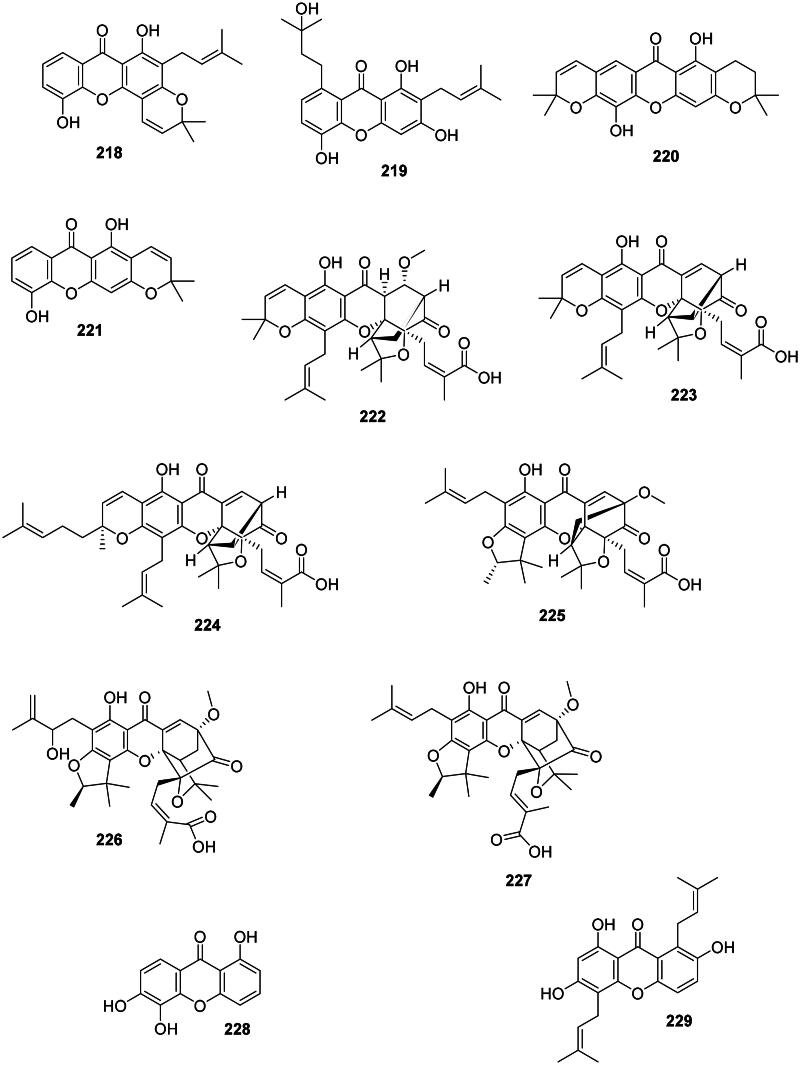

Thai researchers have identified in plants of the *Garcinia* L. genus (Clusiaceae) a plethora of antibacterial xanthones that have often in common dimethylallyl groups at position 1 and or 7. This is the case for 12b-hydroxy-des-d-garcigerrin A (**200**) from *Garcinia dulcis* (Roxb.) Kurz bacteriostatic for MRSA (MIC/MBC: 4/>200 µg/mL) (Thepthong et al. 2017). *Garcinia mangostana* L. produces α-mangostin (**201**) (Nguyen and Marquis 2011) active against VRE (MIC: 3.1 µg/mL) (Sakagami et al. 2005), *B. subtilis* (MIC: 0.5 µg/mL) (Auranwiwat et al. 2014), *S. typhimurium* (Yahayu et al. 2013), and *P. acnes* (MIC: 0.7 µg/mL) (Al-Massarani et al. 2013; Ahmad et al. 2019). α-Mangostin (**201**) and β-mangostin (**202**) inhibited *M. tuberculosis* (MIC: 6.2 µg/mL) as well as garcinone B (**203**) (MIC: 12.7 µg/mL) (Suksamrarn et al. 2003). β-Mangostin (**202**) was active against *B. cereus* with a MIC value as low as 0.2 µg/mL (Auranwiwat et al. 2014). γ-Mangostin (**204**) inhibited the growth of MRSA and VRE with MIC values of 3.1 and 6.2 µg/mL, respectively (Dharmaratne et al. 2013). Garcinone C (**205**) was antileptospiral (Seesom et al. 2013). We can also mention garcicowanone A (**206**), rubraxanthone (**207**), fuscaxanthone A (**208**), 9-hydroxycalabaxanthone (**198**), and garcinianone A (**209**) from *G. cowa*, active against *B. subtilis* with the MIC values of 0.2, 1, 8, 4, and 4 µg/mL, respectively (Trisuwan and Ritthiwigrom 2012; Auranwiwat et al. 2014). From this plant, cowanol (**210**), cowanin (**211**), and garciniacowone (**212**) inhibited *S. aureus* with MIC values of 2, 4, and 2 µg/mL, respectively. Cowanol (**210**) was effective against *E. coli* (MIC: 8 µg/mL) (Siridechakorn et al. 2012) and yielded a MIC value of 2 µg/mL against MRSA (Siridechakorn et al. 2012; Trisuwan and Ritthiwigrom 2012). Other examples are nigrolineaxanthone F (**213**), brasilixanthone (**214**) (MRSA, MIC: 2 µg/mL) (Rukachaisirikul, Tadpetch, et al. 2005), 3-hydroxyblancoxanthone (**215**) (*B. cereus*, MIC: 4 µg/mL), nigrolineaxanthone Q (**216**) (*Micrococcus luteus*, MIC: 8 µg/mL) (Raksat et al. 2019), 8-desoxygartanin (**217**) (*S. aureus*, MIC: 16 µg/mL), ananixanthone (**218**) (*S. aureus*, MIC: 32 µg/mL), and nigrolineaxanthone N (**219**) (MRSA, MIC: 4 µg/mL) from *Garcinia nigrolineata* Planch. ex T. Anderson (Rukachaisirikul et al. 2003). *Garcinia scortechinii* King produces nigrolineaxanthone G (**220**) and 6-deoxyjacareubin (**221**) (MRSA, MIC: 4 µg/mL) (Rukachaisirikul, Tadpetch, et al. 2005).

The internal cyclization of dimethylallyl groups forms caged xanthones, such as moreollic acid (**222**) from *Garcinia hanburyi* Hook.f. (MRSA, MIC: 25 µg/mL), as well as morellic acid (**223**) (Sukpondma et al. 2005) and gambogic acid (**224**) bactericidal for MRSA (USA300) with the MIC/MBC of 12.5/25 and 25/50 µg/mL, respectively (Chaiyakunvat et al. 2016). From *G. scortechinii*, scortechinone B (**225**), C (**226**), and F (**227**) were active against *S. aureus* with the MIC of 2, 8, and 4 µg/mL, respectively (Rukachaisirikul, Phainuphong, et al. 2005).

Non-prenylated and hydroxylated xanthones have milder activities as in 1,5,6-trihydroxyxanthone (**228**) from *Garcinia succifolia* Kurz (*S. aureus*, MIC: 64 µg/mL) (Duangsrisai et al. 2014). In the family Moraceae, gerontoxanthone H (**229**) from *Cudrania cochinchinensis* (Lour.) Kudô & Masam. was active against *B. cereus* with the MIC of 1.5 μg/mL (Fukai et al. 2004).

#### Chromanes and chromenes

**Figure 19.**
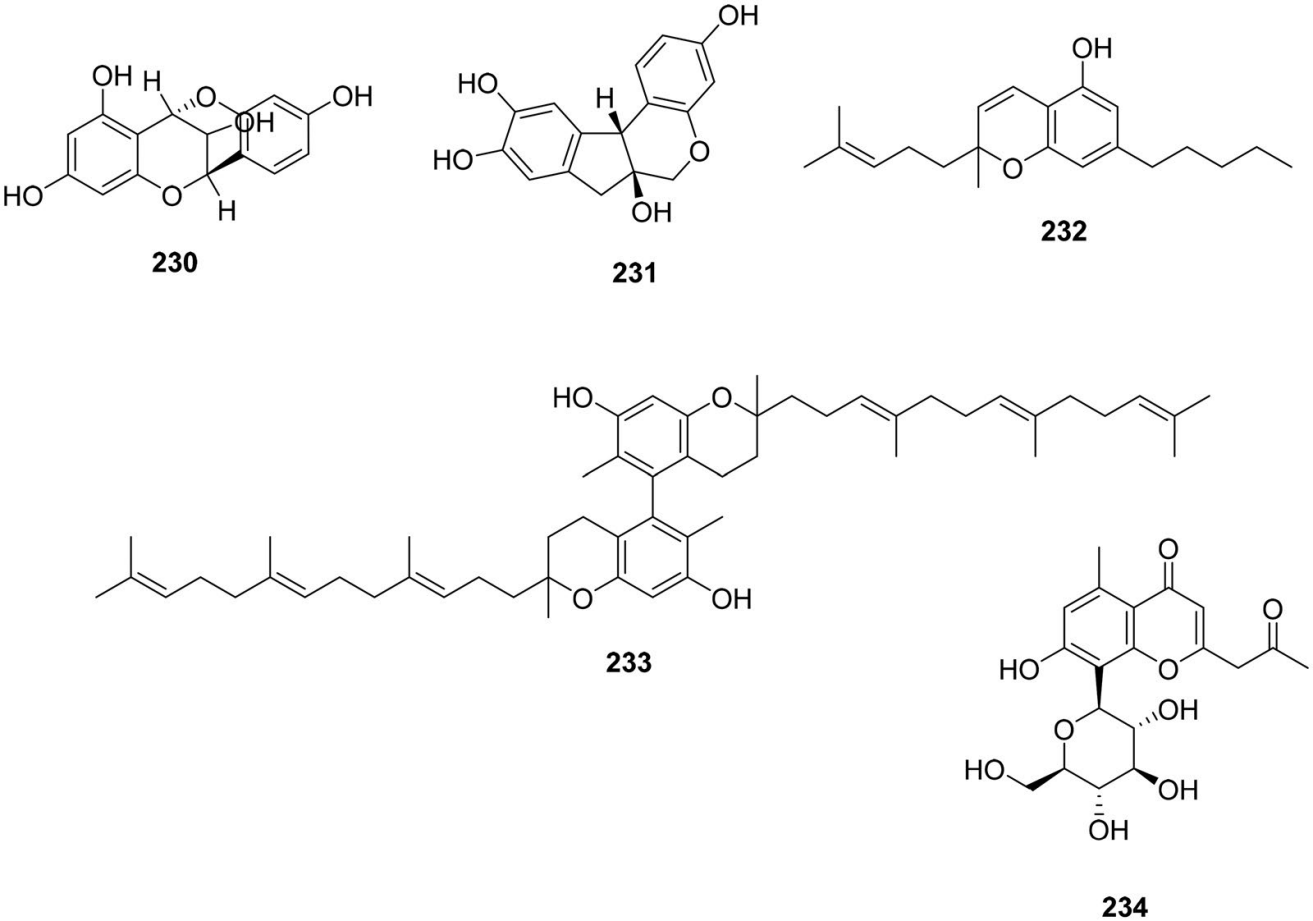
Chromanes and chromenes.

Plants in the fabids and malvids produce antibacterial chromanes and chromenes (Figure 19). For instance, cyanomaclurin (**230**) from *Artocarpus heterophyllus* Lam. (Moraceae) was bacteriostatic for *S. mutans* (Septama and Panichayupakaranant 2015). Brasilin (**231**) from *Caesalpinia sappan* L. (Fabaceae) used in traditional Chinese medicine was active against MRSA, VRE, and MDR-*Burkholderia cepacia* (Xu and Lee 2004) as well as *S. pyogenes* (MIC: 4 µg/mL) (Yin et al. 2004). *C. sativa* produces cannabichromene (**232**) (*S. aureus*, MIC: 2 µg/mL) (Appendino et al. 2008; Radwan et al. 2009). The dimeric prenylated chromane garciniacowol (**233**) from *G. cowa* inhibited MRSA with the MIC of 2 µg/mL (Siridechakorn et al. 2012). The chromane glycoside aloesin (**234**) in the genus *Rumex* L. (Polygonaceae) was active against *M. tuberculosis* (MIC: 2.8 µM) (Liang et al. 2010).

#### Naphthalenols

**Figure 20.**
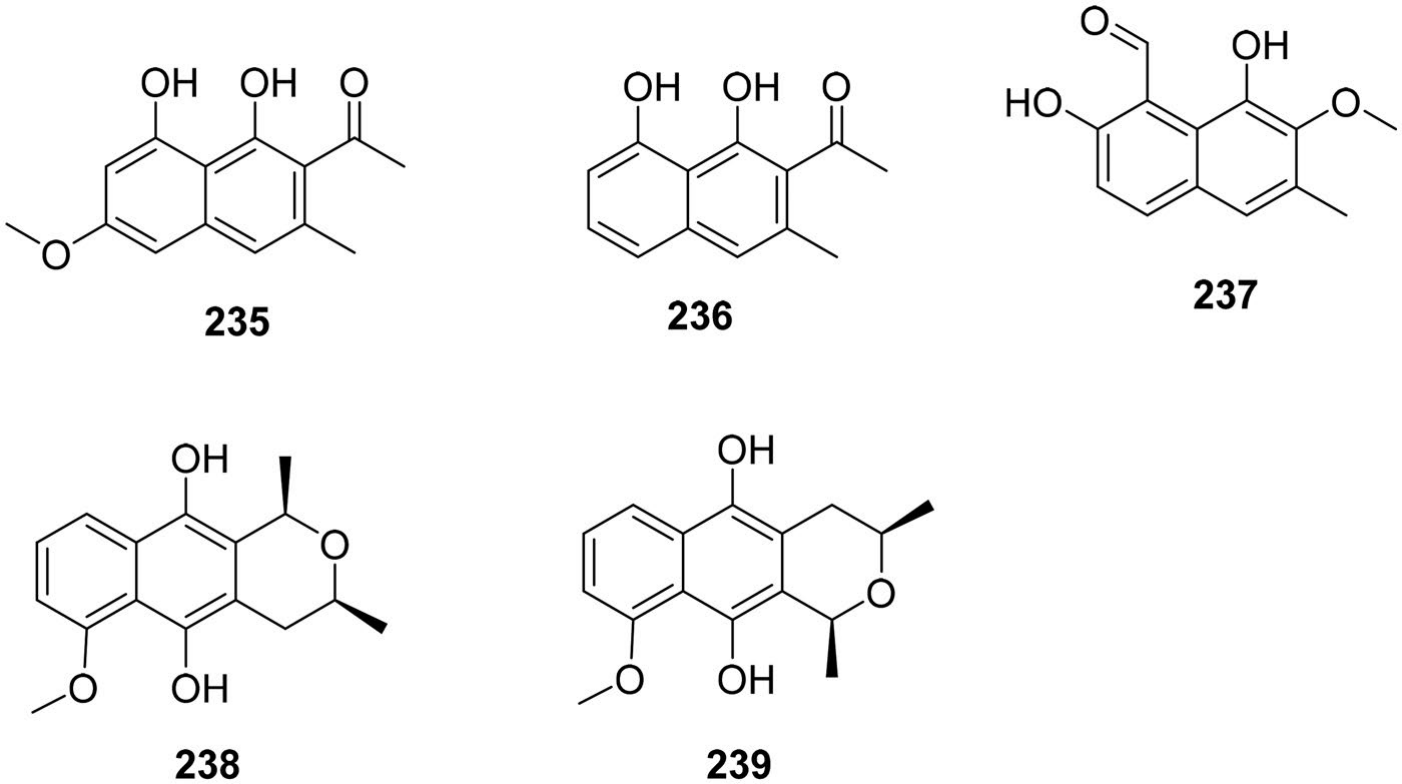
Naphthalenols.

They are found in the malvids. Examples are torachrysone (**235**) in *Rumex japonicus* Houtt. (Polygonaceae) active against MRSA (MIC: 32 µg/mL) (Hatano et al. 1999) and *M. tuberculosis* (Nishina et al. 1993; Liang et al. 2010), nepodin (**236**) from *Rumex aquaticus* L. (Polygonaceae) (Orbán-Gyapai 2017), and hibicuslide C (**237**) from *Hibiscus taiwanensis* S.Y. Hu (Malvaceae) (MDR-*P. aeruginosa*) (Lee, Choi, et al. 2016). In the monocots, *Eleutherine bulbosa* (Mill.) Urb. (Iridaceae) produces eleubosas A (**238**) and B (**239**) (*E. coli*, MIC: 12.5 µg/mL) (Jiang et al. 2020) (Figure 20).

#### Phenanthrenes

**Figure 21.**
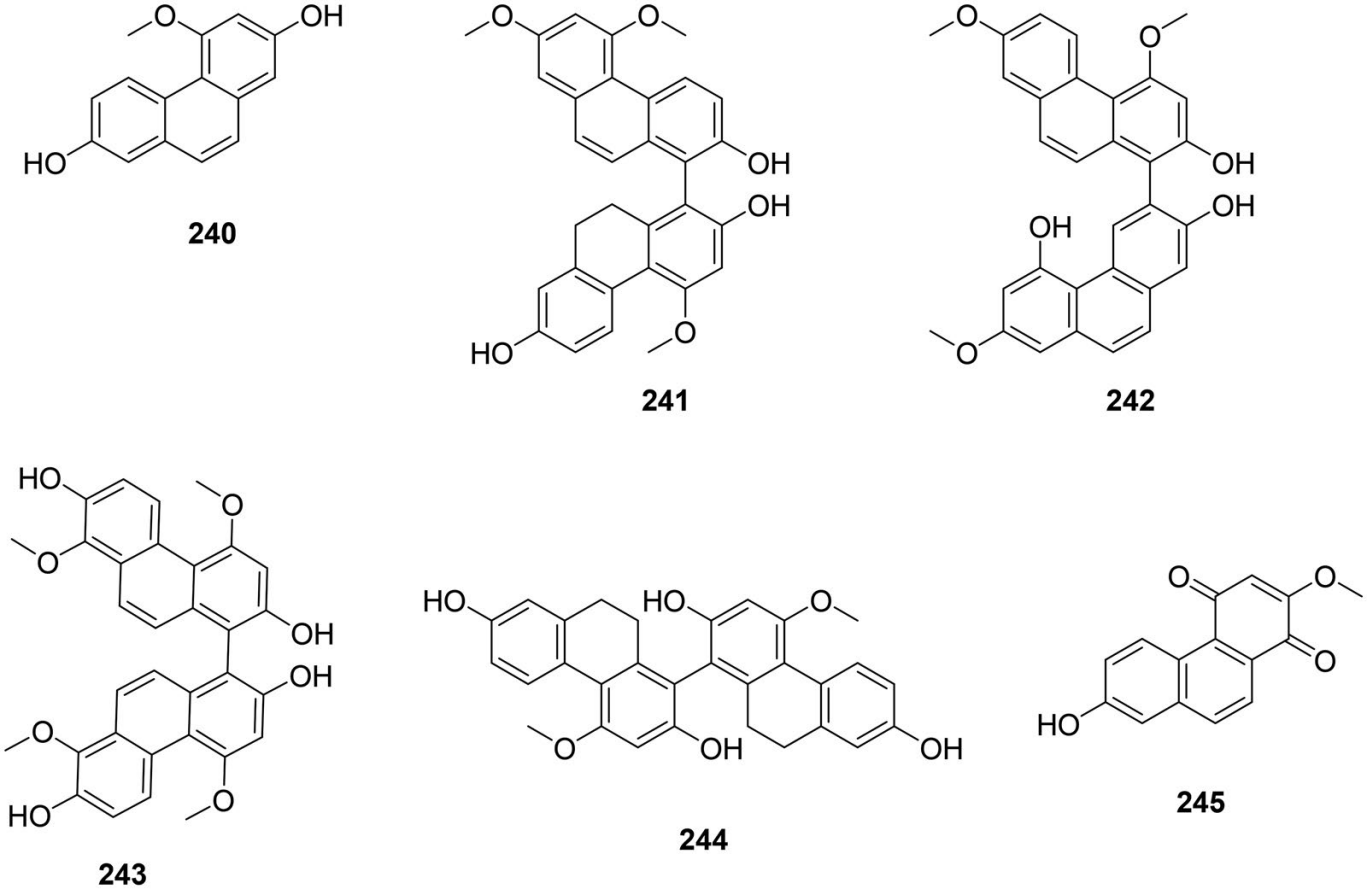
Phenanthrenes.

Internal cyclization of stilbenes forms antibacterial phenanthrenes and biphenanthrenes in monocots (Chapatwala et al. 1981) (Figure 21). Examples are 2,7-dihydroxy-4-methoxyphenanthrene (**240**) from *Dioscorea bulbifera* L. (Dioscoreaceae) (Kuete et al. 2012) and blestriacin (**241**) from *B. striata* (MRSA, MIC: 2 μg/mL, bactericidal) (Chen et al. 2018). *Bletilla striata* produces 4,7,7′-trimethoxy-9′,10′-dihydro(1,3′-biphenanthrene)-2,2′,5′-triol (**242**) (*S. aureus*, MIC: 8 µg/mL) as well as 4,8,4′,8′-tetramethoxy(1,1′-biphenanthrene)-2,7,2′,7′-tetrol (**243**) (*S. aureus*, bactericidal) (4,8,4′,8′-TBT) (Qian et al. 2015). *Arundina graminifolia* (D. Don) Hochr (Orchidaceae) yields blestiarene A (**244**) and densiflorol B (**245**) bacteriostatic for *S. aureus* and *E. coli* (Zhang et al. 2022).

#### Phenylphenalenones

**Figure 22.**
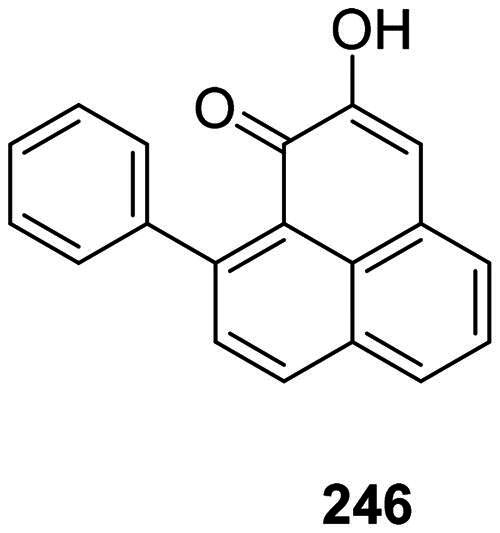
Phenylphenalenones.

Diarylheptanoids serve in the monocots as precursors for the synthesis of phenylphenalenones phytoalexins, such as anigorufone (**246**) from *Macropidia fuliginosa* (Hook.) Druce (Haemodoraceae) (Brkljaca et al. 2019) (Figure 22). Other antibacterial phenylphenalenones are found in the genus *Musa* L. (Musaceae) (Krishnamurthy et al. 2023).

#### Tetralones

**Figure 23.**
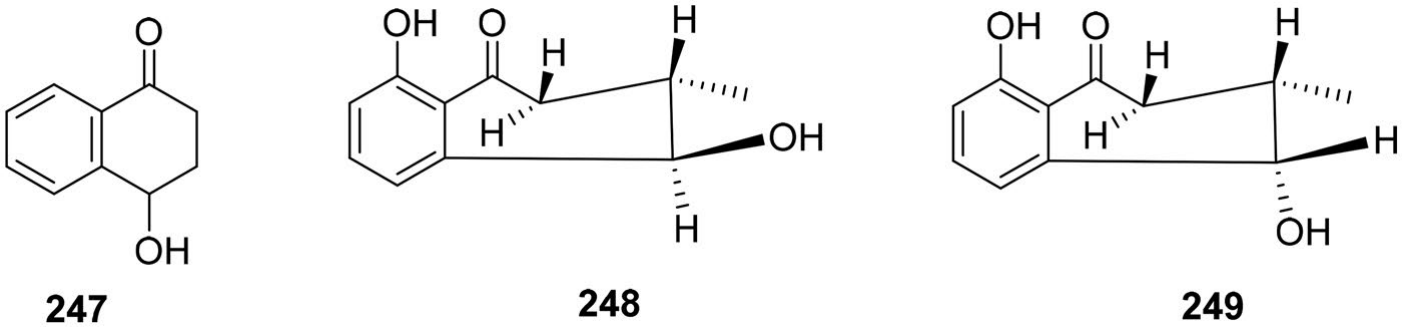
Tetralones.

Examples are 4-hydroxy-1-tetralone (**247**) from *E. roxburghiana* (*M. tuberculosis*, MIC: 4 µg/mL) (Lin et al. 2005; Wu et al. 2012), l-*epineo*-isoshinanolone (**248**) (*E. coli*, MIC: 12.5 µg/mL), and *neoiso*-shinanolone (**249**) from *Plumbago zeylanica* L. (Plumbaginaceae, malvids) (Jetty et al. 2010) (Figure 23).

### Other non-flavonoids

**Figure 24.**
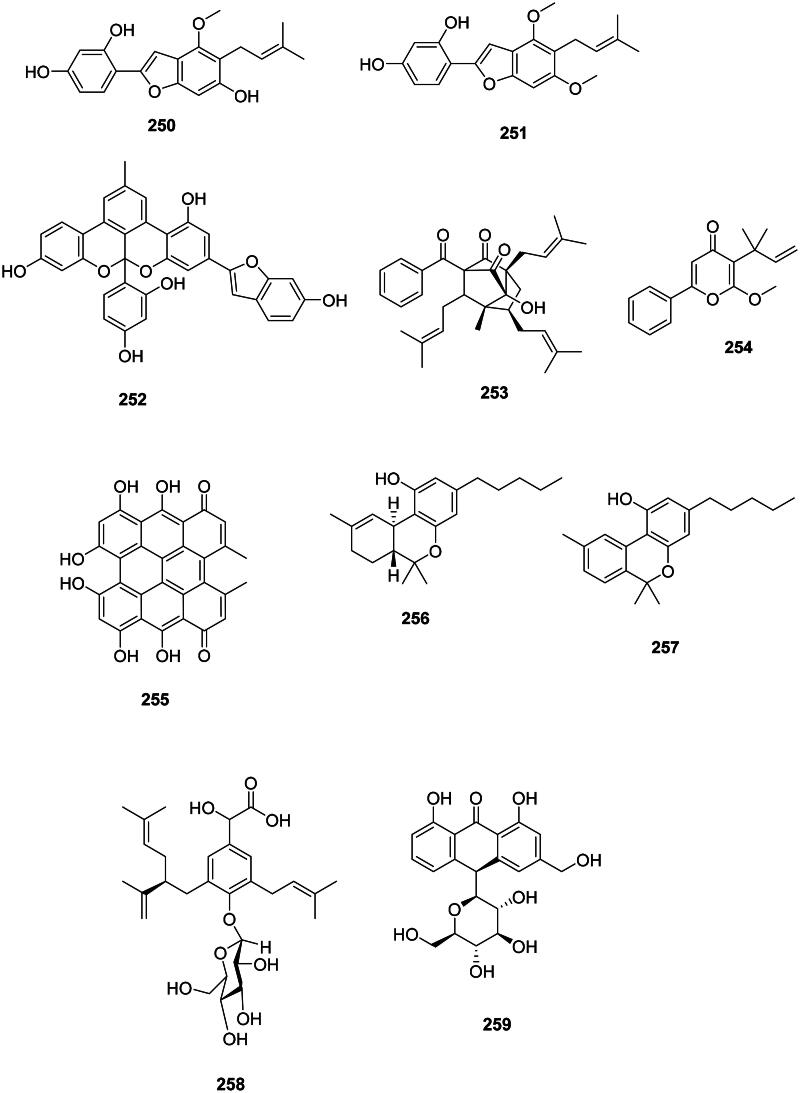
Other non-flavonoid phenolic compounds.

They mostly occur in fabids (Figure 24). Examples are licocoumarone (**250**) (*S. aureus*, MIC: 6.2 µg/mL) from *G. glabra* (Demizu et al. 1988), gancaonin I (**251**) (MRSA, MIC: 1.5 µg/mL) from *Glycyrrhiza uralensis* Fisch. ex DC. (Fabaceae) (Fukai et al. 2002), and albanol B (**252**) from *Morus alba* L. (Moraceae) (*S. typhimurium*, MIC: 5 µg/mL) (Park et al. 2003, Sohn et al. 2004).

In the family Hypericaceae, examples are hypatulin A (**253**) from *Hypericum patulum* Thunb. (*B. subtilis*, MIC: 16 μg/mL) (Tanaka et al. 2016), hyperenone A (**254**) from *H. acmosepalum* (*S. aureus* expressing NorA, MIC: 2 µg/mL) (Osman et al. 2012), and hypericin (**255**) from *Hypericum perforatum* L. (Feyzioğlu et al. 2013).

Δ_9_-Tetrahydrocannabinol (**256**) and cannabinol (**257**) from *C. sativa* are formed *via* the polyketide pathway (Abe 2020) and were very strongly active against *S. aureus* (MIC: 1 µg/mL) (Appendino et al. 2008; Radwan et al. 2009). We can also cite harpulliaside A (**258**) from *Harpullia pendula* Planch. ex F. Muell. (Sapindaceae) (*V. parahaemolyticus*, MIC: 35 µg/mL) (Abdelkader et al. 2016) as well as aloin A (**259**) from *A. vera* (Coopoosamy and Magwa 2006b).

## Flavonoids

### Chalcones

**Figure 25.**
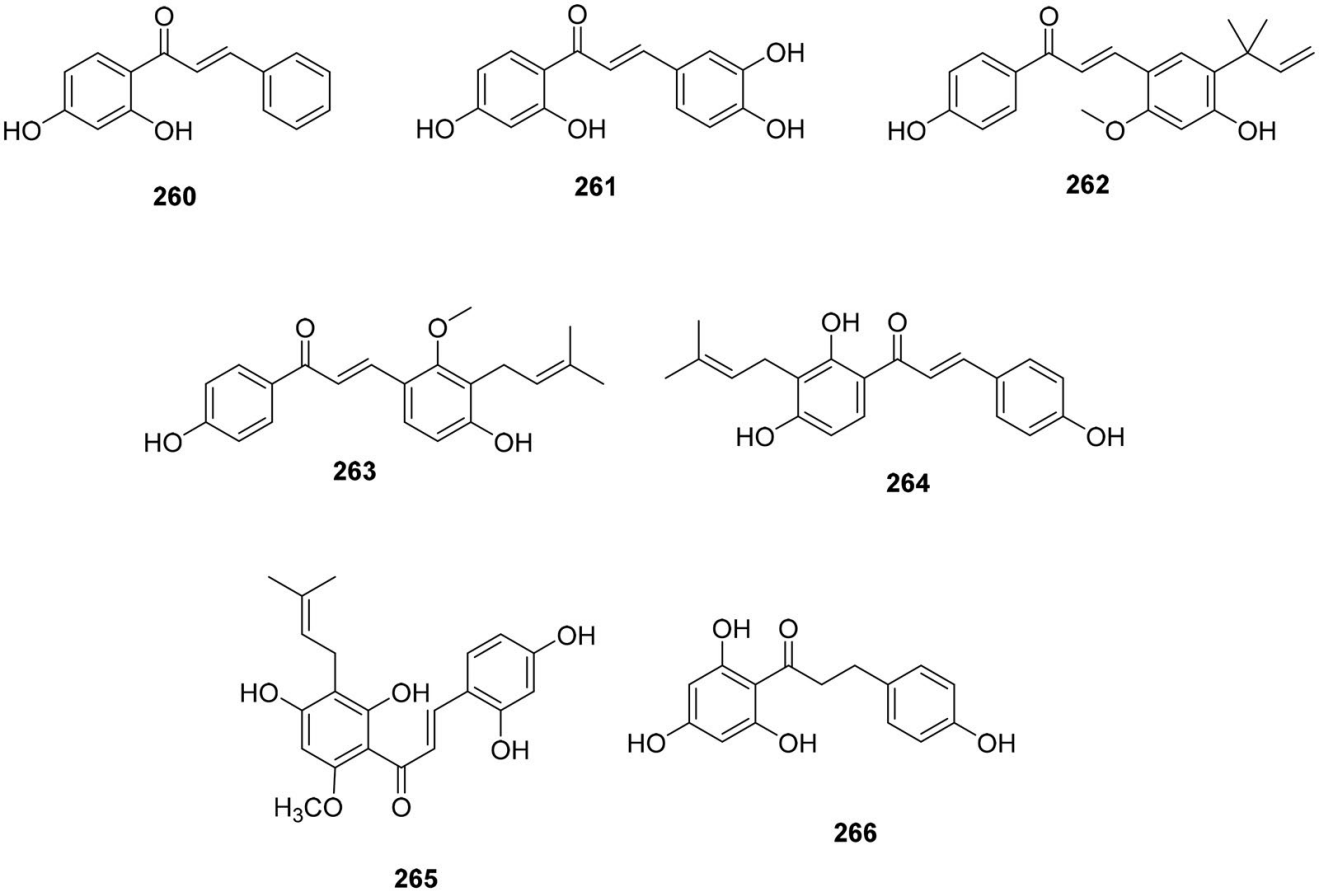
Chalcones.

The addition of one hydroxycinnamic acid unit with three malonyl-CoA units forms chalcones (Abe et al. 2006; Wang et al. 2023) (Figure 25). These 1,3-diphenyl-2-propen-1-ones occur in the fabids and malvids and are active against Gram-positive bacteria and mycobacteria. Examples are 2′,4′-dihydroxychalcone (**260**) from *Muntingia calabura* L. (Mutingiaceae, malvids) bactericidal for *S. aureus* (MIC/MBC: 50/100 µg/mL) (Sufian et al. 2013) and butein (**261**) from *Butea monosperma* (Lam.) Taub. (Fabaceae) (*M. tuberculosis*, MIC: 12.5 µg/mL) (Chokchaisiri et al. 2009).

The prenylation of chalcones in the family Fabaceae enhances their antibacterial activity as in licochalcone A (**262**) from *Glycyrrhiza inflata* Batalin with *S. aureus* (MIC: 3 µg/mL) (Tsukiyama et al. 2002), MRSA (MIC: 6.2 µg/mL), and *P. gingivalis* (MIC: 10 µg/mL) (Fukai et al. 2002). Other examples are licochalcone C (**263**) (*M. luteus*, MIC: 6.2 µg/mL) (Haraguchi et al. 1998) and isobavachalcone (**264**) from *P. corylifolia* (Yin et al. 2004). *Sophora flavescens* Aiton is used in traditional Chinese medicine and produces 7,9,2′,4′-tetrahydroxy-8-isopentenyl-5-methoxychalcone (7,9,2′,4′-TIMC) (**265**) which was effective against MRSA (MIC: 0.9 µg/mL) and VRE (MIC: 7.8 µg/mL) (Lee, Kim, et al. 2010).

The condensation of one dihydrohydroxycinnamic acid unit with three malonyl-CoA units forms antibacterial dihydrochalcones (Ibdah et al. 2017) such as phloretin (**266**) from *Malus domestica* (Suckow) Borkh. (Rosaceae, fabids) (*S. aureus*, MIC: 7.8 µg/mL) (Barreca et al. 2014).

### Flavanones

#### Simple flavanones

**Figure 26.**
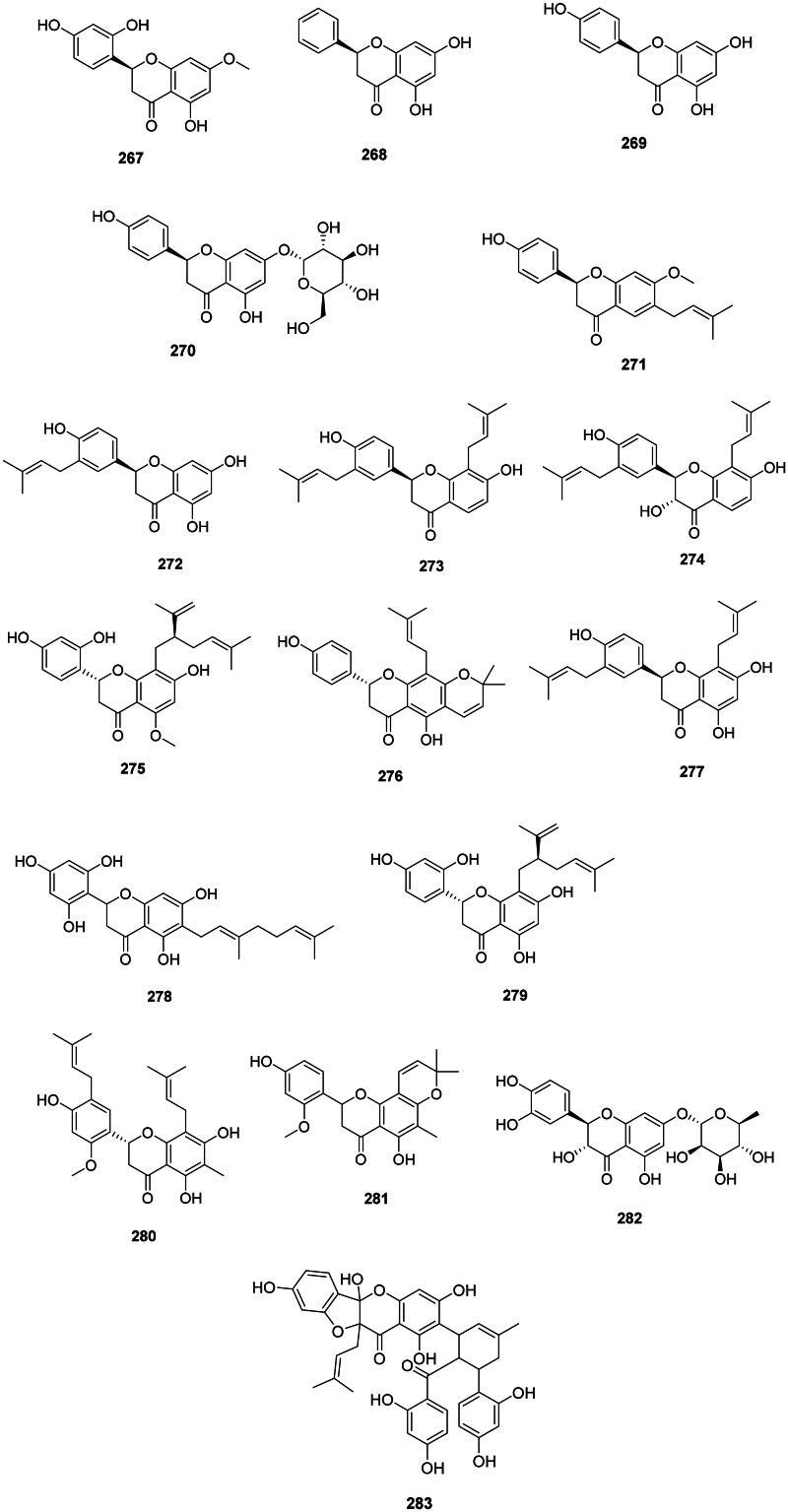
Flavanones.

These antibacterial 2-phenyl-2,3-dihydro-4H-chromen-4-ones come from the cyclization of chalcones in malvids and fabids (Shah and Smith 2020) (Figure 26). Examples are artocarpanone (**267**) from *A. heterophyllus* bactericidal for *E. coli* (MIC/MBC: 3.9/7.8 µg/mL) (Septama and Panichayupakaranant 2017), pinocembrin (**268**) from *G. glabra* (*M. tuberculosis*, MIC: 3.3 µg/mL) (Fukui et al. 1988; Chou et al. 2011), and naringenin (**269**) in the genus *Citrus* L. (Rutaceae) (*S. pyogenes*, MIC: 50 µg/mL) (Macé et al. 2017).

Glycosylation of naringenin (**269**) in position 8 forms prurin (**270**) in *Acacia farnesiana* Wall. (Fabaceae) active against MDR-*M. tuberculosis* (MIC: 50 µg/mL) and *C. jejuni* (MIC: 50 µg/mL) (Hernández-García et al. 2019).

#### Prenylated flavanones

Plants of the Fabaceae family produce prenylated flavanones active against Gram-positive bacteria (Figure 26). Examples of flavanones with dimethylallyl moieties are bavachinin (**271**) from *P. corylifolia* (Yin et al. 2004) and licoflavanone (**272**) in *G. glabra* (Fukui et al. 1988). From this plant, glabrol (**273**) and 3-hydroxyglabrol (**274**) yielded the MIC values of 1.5 and 6.2 µg/mL against *S. aureus*, respectively (Mitscher et al. 1980). Glabrol (**273**) was active against *M. smegmatis* (MIC: 1.5 µg/mL) (Mitscher et al. 1980). Other instances are kurarinone (**275**) from *S. flavescens* (MRSA, MIC: 2 µg/mL) (Chen et al. 2005), lupinifolin (**276**) from *Derris reticulata* Craib. (Mazimba et al. 2012) (*S. aureus*, MIC: 8 µg/mL, bactericidal) (Yusook et al. 2017), and euchrestaflavanone A (**277**) from *Flemingia strobilifera* (L.) W.T. Aiton (*P. aeruginosa*, MIC: 17 µg/mL) (Madan et al. 2008).

An example of geranylated flavanone is sophoraflavanone D (**278**) from *Echinosophora koreensis* Nakai (Fabaceae) (*E. coli* MIC: 20 µg/mL) (Sohn et al. 2004). A lavandulyl group enhances the antibacterial strength of flavanones as in sophoraflavanone G (**279**) from *Sophora exigua* Craib. (Fabaceae) with a MIC as low as 0.5 µg/mL against MRSA (Cha et al. 2007, 2009).

Plants of the Celastraceae family (fabids) produce antibacterial flavanones. Examples are (2*S*)-5,7,4′-trihydroxy-2′-methoxy-8,5′-di(3-methyl-2-butenyl)-6-methylflavanone (**280**) and (±)-5,4′-dihydroxy-2′-methoxy-6′,6″-dimethypyraro-(2″,3″:7,8)-6-methyflavanone (**281**) (MRSA, IC_50_: 2 µg/mL) from *Tripterygium wilfordii* Hook.f. used in traditional Chinese medicine (Chen et al. 2017).

#### Flavanone-O-glycosides

Taxifolin-7-*O*-rhamnoside (**282**) from *H. japonicum* was bactericidal for MRSA (MIC/MBC: 32/64 µg/mL) (An et al. 2011).

#### Miscellaneous

There are flavones with complex polyphenolic structures, such as sanggenon D (**283**) from *Morus alba* L. (Moraceae) active against *S. epidermidis* (MIC: 40 µg/mL) (Sohn et al. 2004).

### Isoflavans

**Figure 27.**
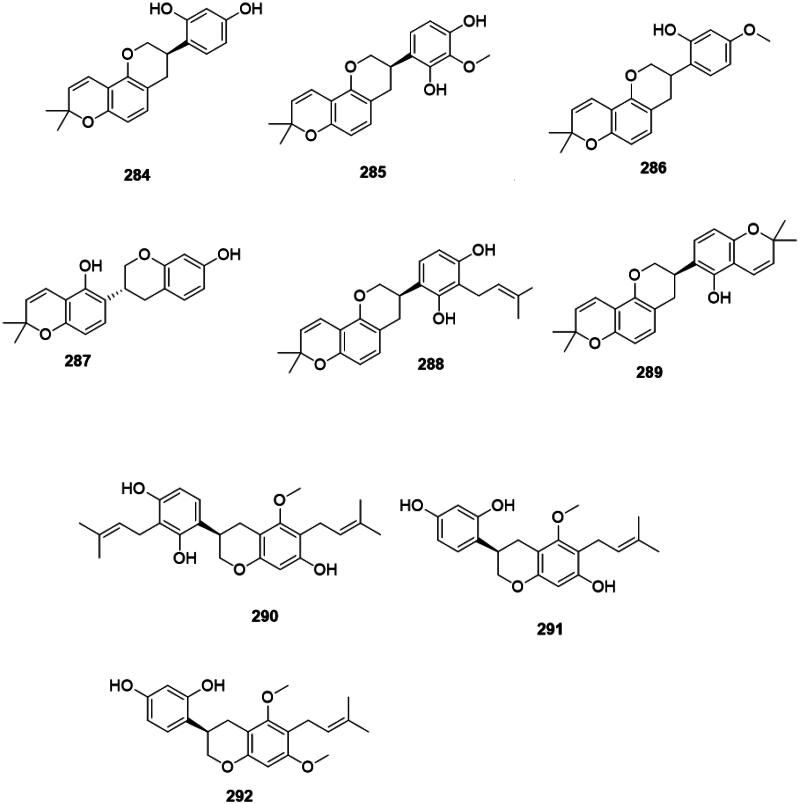
Isoflavans.

Plants of the Fabaceae produce antibacterial isoflavans (Pičmanová et al. 2013) (Figure 27). Glabridin (**284**) from *G. glabra* inhibited the growth of *S. aureus* (MIC: 6.2 µg/mL) (Gupta et al. 2008), MRSA (MIC: 12.5 µg/mL) (Fukai et al. 2002), *M. smegmatis* (6.2 µg/mL) (Mitscher et al. 1980), and *P. gingivalis* (MIC: 10 µg/mL) (Azelmat et al. 2015). From *G. glabra*, other antibacterial isoflavans are 3′-methoxyglabridin (**285**), 4′-*O*-methylglabridin (**286**), phaseollinisoflavan (**287**), hispaglabridin A (**288**) (*S. aureus*, MIC: 3.1 µg/mL), and hispaglabridin B (**289**) (*S. aureus*, MIC: 6.2 µg/mL) (Mitscher et al. 1980). Other examples include licoricidin (**290**) (MRSA, MIC: 3.1 µg/mL), glyasperin C (**291**) (*E. faecium*), and glyasperin D (**292**) (MRSA, MIC: 6.2 µg/mL) from *G. uralensis* (Fukai et al. 2002; Gafner et al. 2011; Eerdunbayaer et al. 2014; Villinski et al. 2014).

### Isoflavanones

**Figure 28.**
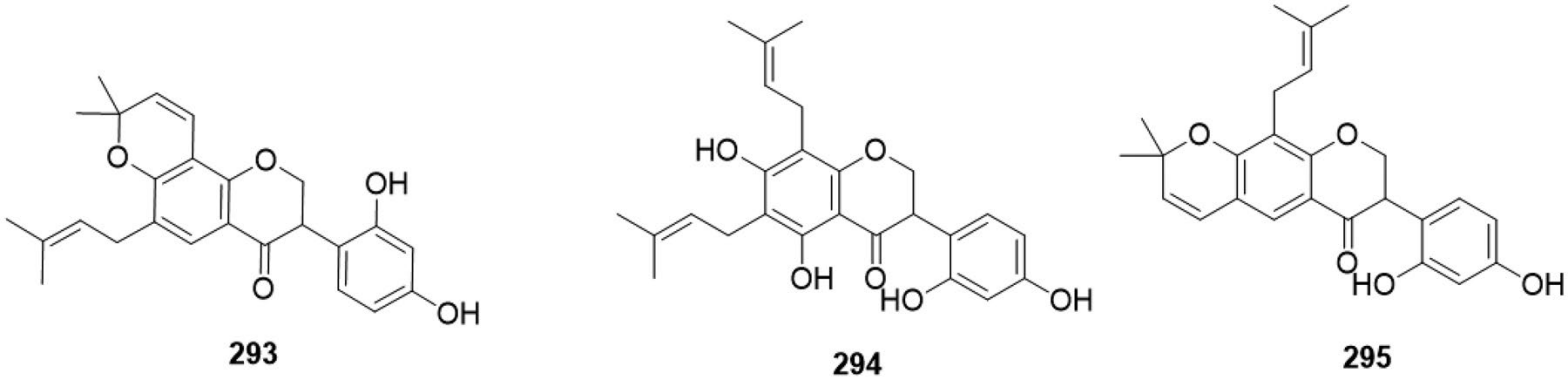
Isoflavanones.

Plants of the Fabaceae family isomerize flavanones into isoflavanones active against Gram-positive bacteria (Pičmanová et al. 2013). For example, *Erythrina variegata* L. (Fabaceae) produces orientanol F (**293**) (MRSA, MIC_90_: 12.5 µg/mL) (Tanaka et al. 2002), orientanol E (**294**) (MRSA, MIC_90_/MBC_90_: 3.1/25 µg/mL, bacteriostatic) (Tanaka et al. 2015), and bidwillon B (**295**) (MRSA, MIC: 3.1 µg/mL) (Sato et al. 2003) (Figure 28).

### Flavones

#### Simple flavones

The formation of a Δ^2,3^ double bond in flavanones forms flavones that generally have moderate antibacterial activities (Figure 29) (Zhao et al. 2016). Flavones that have an unsubstituted C ring are common in upper Angiosperms. This is the case, for example, of chrysin (**297**) of *Oroxylum indicum* (L.) Kurz (Bignoniaceae, lamiids) (Ali et al. 1998;  Zhao et al. 2022) and baicalein (**298**) (*S. typhimurium*, MIC: 64 μg/mL) in *Scutellaria baicalensis* Georgi (Lamiaceae, lamiids) used in traditional Chinese medicine (Yang et al. 2000; Wu et al. 2018). Another example is galangin (**299**) from *Helichrysum aureonitens* Sch. Bip. (Asteraceae, campanulids) which was active against 4-quinolone-resistant *S. aureus* (Cushnie and Lamb 2006) and *Mycobacterium phlei* (MIC: 50 µg/mL) (Pomilio et al. 1992).

Figure 29. Flavones.

**Figure:**
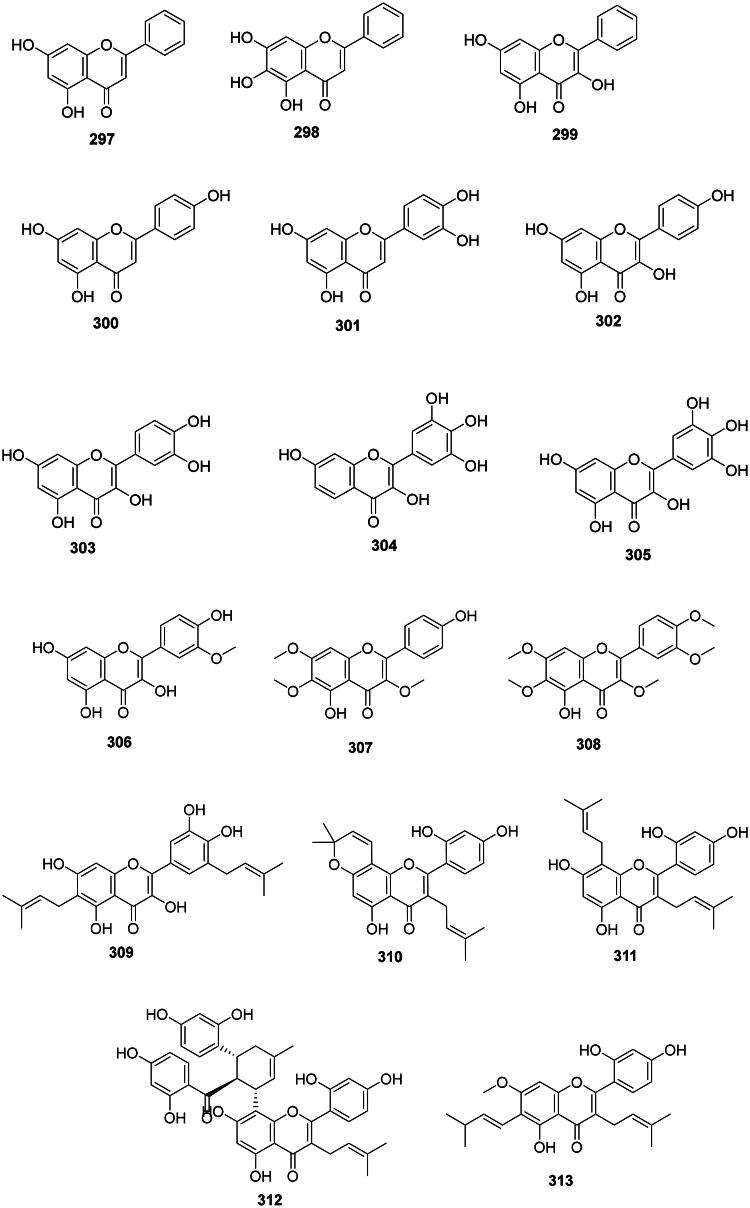

**Figure:**
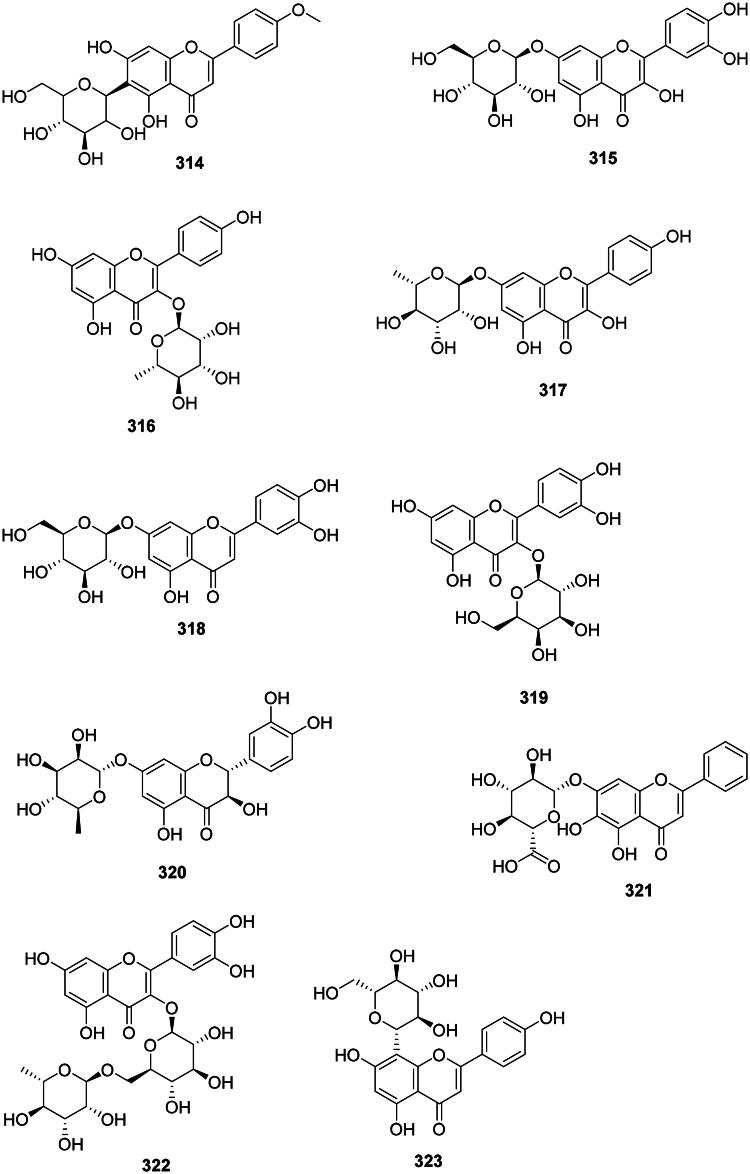

Flavones with hydroxylated C rings are ubiquitous in Angiosperms and include notably apigenin (**300**) (*K. pneumoniae*, MIC: 25 µg/mL), luteolin (**301**) (*P. aeruginosa*, MIC: 25 µg/mL) (Sathiamoorthy et al. 2007; Bustos et al. 2018), kaempferol (**302**) (*S. mutans*, MIC: 32 µg/mL) (Yamada et al. 1999), quercetin (**303**) (*Salmonella enteridis*, MIC: 15.6 µg/mL) (Phadungkit and Luanratana 2006), robinetin (**304**) (Mori et al. 1987), and myricetin (**305**) (Xu et al. 2015).

#### Methoxylated flavones

Upper Angiosperms often produce antibacterial methoxylated flavones (Figure 29). We can cite for instance isorhamnetin (**306**) from *A. elliptica* (*S. typhimurium*, MIC: 15.6 µg/mL) (Phadungkit and Luanratana 2006). *Vitex negundo* L. (Verbenaceae) produces penduletin (**307**) (MRSA, MIC: 10 µg/mL) (Sichaem et al. 2021) and artemetin (**308**) (*K. pneumoniae*, MIC: 25 µg/mL) (Sathiamoorthy et al. 2007). Other examples are found in the family Asteraceae (Murillo et al. 2003).

#### Prenylated flavones

They are common in the family Moraceae and include papyriflavonol A (**309**) from *Broussonetia papyrifera* (L.) L’Hér. ex Vent. (*S. typhimurium*, MIC: 10 µg/mL), morusin (**310**) from *Morus mongolica* (Bureau) C.K. Schneid. (*S. epidermidis*, MIC: 20 µg/mL), kuwanon C (**311**) (*S. typhimurium*, MIC: 6.2 µg/mL) (Sohn et al. 2004), kuwanon G (**312**) (*S. mutans*, MIC: 8 µg/mL) from *M. alba* (Park et al. 2003), and artocarpin (**313**) from *A. heterophyllus* (*S. mutans*, MIC/MBC: 4.4/8.9 µM) (Septama and Panichayupakaranant 2018) (Figure 29).

#### Flavone-O-glycosides

Examples are isocytisoside (**314**) from *Aquilegia vulgaris* L. (Ranunculaceae) (*S. aureus*, MIC: 15.6 µg/mL) (Bylka et al. 2004), quercetin 7-*O*-glucoside (**315**) in *Gossypium arboreum* L. (Malvaceae) (Waage and Hedin 1984) (Figure 29). Other illustrations are afzelin (**316**) and kaempferol-7-rhamnoside (**317**) from *Bryophyllum pinnatum* (Lam.) Oken (Crassulaceae, core eudicots) active against *S. typhi* with the MIC values of 2 and 1 µg/mL, respectively (Tatsimo et al. 2012), luteoloside (**318**) from *L. japonica* (Xiong et al. 2013), hyperoside (**319**) from *H. perforatum* (Pretorius et al. 2003; Saçıcı and Yesilada 2022), as well as taxifolin-7-*O*-α-l-rhamnopyranoside (**320**) (TLRP) (An et al. 2011). Baicalin (**321**) from *S. baicalensis* inhibited the growth of *S. typhimurium* (MIC/MBC: 64/>128 μg/mL) (Wu et al. 2018). Rutin (**322**) from *Sophora japonica* L. (Fabaceae) (Balbaa et al. 1974) was active *P. aeruginosa*, *A. baumannii*, and *S. aureus* with the MIC values of 16, 8, and 4 µg/mL, respectively (Orhan et al. 2010).

#### Flavone-C-glycosides

Apigenin-8-*C*-glucopyranoside (vitexin) (**323**) (Figure 29) from *V. negundo* inhibited the growth of *Mycobacterium fortuitum* (MIC: 30 µg/mL) (Aderogba et al. 2019). It should be noted that vitexin (**323**), although weakly active *in vitro* against *S. aureus*, was nevertheless active *in vivo* against *S. aureus* with an additional inflammatory action (Das et al. 2022).

### Isoflavones

The isomerization and dehydrogenation of flavanones form isoflavones in the family Fabaceae (Artigot et al. 2013) (Figure 30). These phytoalexins are often active against Gram-positive bacteria and include for instance biochanin A (**324**) from *Cassia fistula* L. (Sartorelli et al. 2009) (*S. pyogenes*, MIC: 32 µg/mL) (Pohjala et al. 2012; Hummelova et al. 2015), santal (**325**) from *Derris scandens* (Roxb.) Benth. (MRSA, MIC: 2 µg/mL) (Mahabusarakam et al. 2004), and formononetin (**326**) from *Glycyrrhiza pallidiflora* Maxim. (Kajiyama et al. 1993; Mutai et al. 2015). In the family Iridaceae (monocots), tectorigenin (**327**) from *Belamcanda chinensis* (L.) Redouté was active against *S. aureus* (MIC: 50 µg/mL) (Oh et al. 2001) and MRSA (MIC: 125 µg/mL) (Joung et al. 2014).

Figure 30. Isoflavones.

**Figure:**
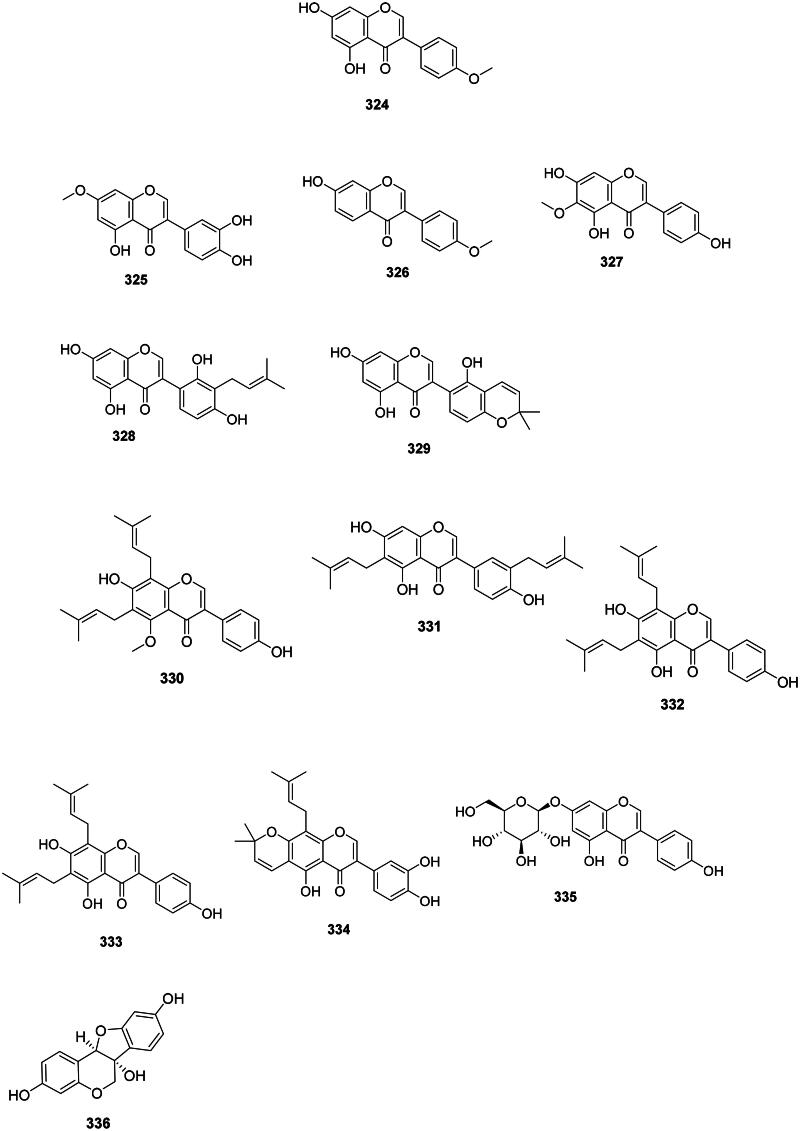

**Figure:**
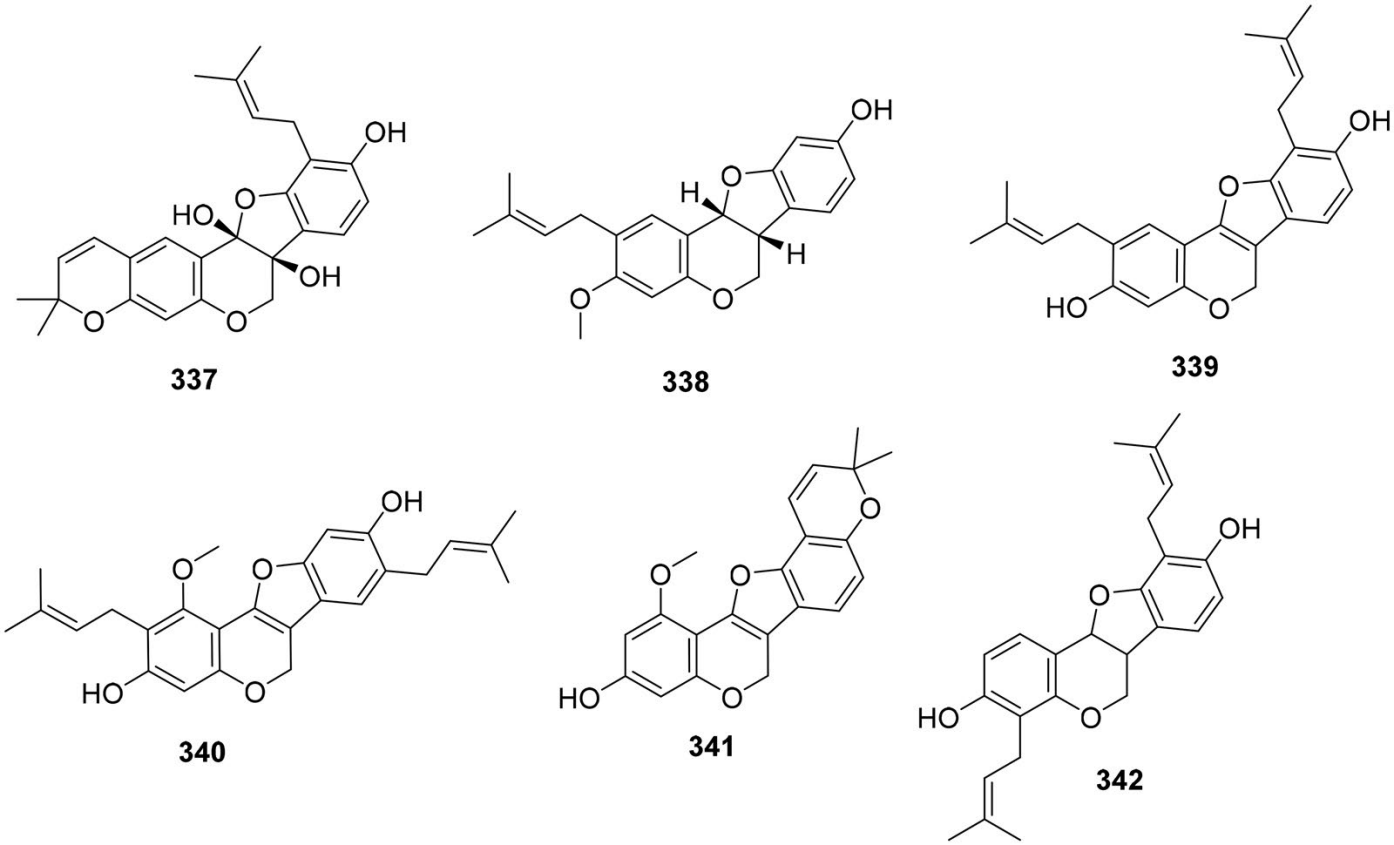

#### Prenylated isoflavones

They are prevalent in the Fabaceae family and are active against Gram-positive bacteria (Figure 30). Examples are licoisoflavone A (**328**) and B (**329**) from *G. uralensis* (MRSA, MIC: 32 µg/mL) (Wu et al. 2019; Chen et al. 2023), derrisisoflavone A (**330**) from *D. scandens* (MRSA, MIC: 4 µg/mL) (Mahabusarakam et al. 2004), lupalbigenin (**331**) from *G. dulcis* (*S. aureus*, MIC: 4 µg/mL) (Deachathai et al. 2005), 6,8-diisoprenyl-5,7,4′-trihydroxyisoflavone (**332**) (*S. mutans*, MIC: 2 µg/mL) (He et al. 2006), 8-(γ,γ-dimethylallyl)-wighteone (**333**) from *G. uralensis* (MRSA, MIC: 8 µg/mL) (Hatano et al. 2000; Eerdunbayaer et al. 2014), and auriculasin (**334**) from *Flemingia philippinensis* Merr. & Rolfe with MDR-*E. coli* (MIC: 2 µg/mL) (Mohamed et al. 2022).

#### Isoflavone glycosides

Genistin (**335**) from *F. strobilifera* inhibited the growth of *S. epidermidis*, *S. aureus*, MRSA, *P. aeruginosa*, and *E. coli* with the MIC of 31.2, 62.5, 34, 125, and 146 µg/mL, respectively (Madan et al. 2008; Boutaghane et al. 2019) (Figure 30).

#### Pterocarpans

The reduction and cyclization of isoflavones form pterocarpans in the family Fabaceae. These phytoalexins are strongly active against Gram-positive bacteria (Figure 30). Examples are glycinol (**336**) from *Glycine max* (L.) Merr. (Weinstein and Albersheim 1983), orientanol C (**337**) from *E. variegata* (MRSA, MIC_90_:12.5 µg/mL), as well as orientanol B (**338**) (MIC: 3.1 µg/mL) (Tanaka et al. 2002). From this plant, erycristagallin (**339**) was active against MRSA (MIC_90_: 6.2 µg/mL) (Tanaka et al. 2002) and *Actinomyces viscosum* (MIC: 1.5 µg/mL) (Sato et al. 2003). Glycyrrhizol A (**340**) and B (**341**) from *G. uralensis* inhibited *S. mutans* with the MIC values of 1 and 32 µg/mL, respectively (He et al. 2006). Erybraedin A (**342**) from *E. zeyheri* was bacterostatic for VRE (MIC: 1.5 µg/mL) (Sato et al. 2004).

## Flavans

**Figure 31.**
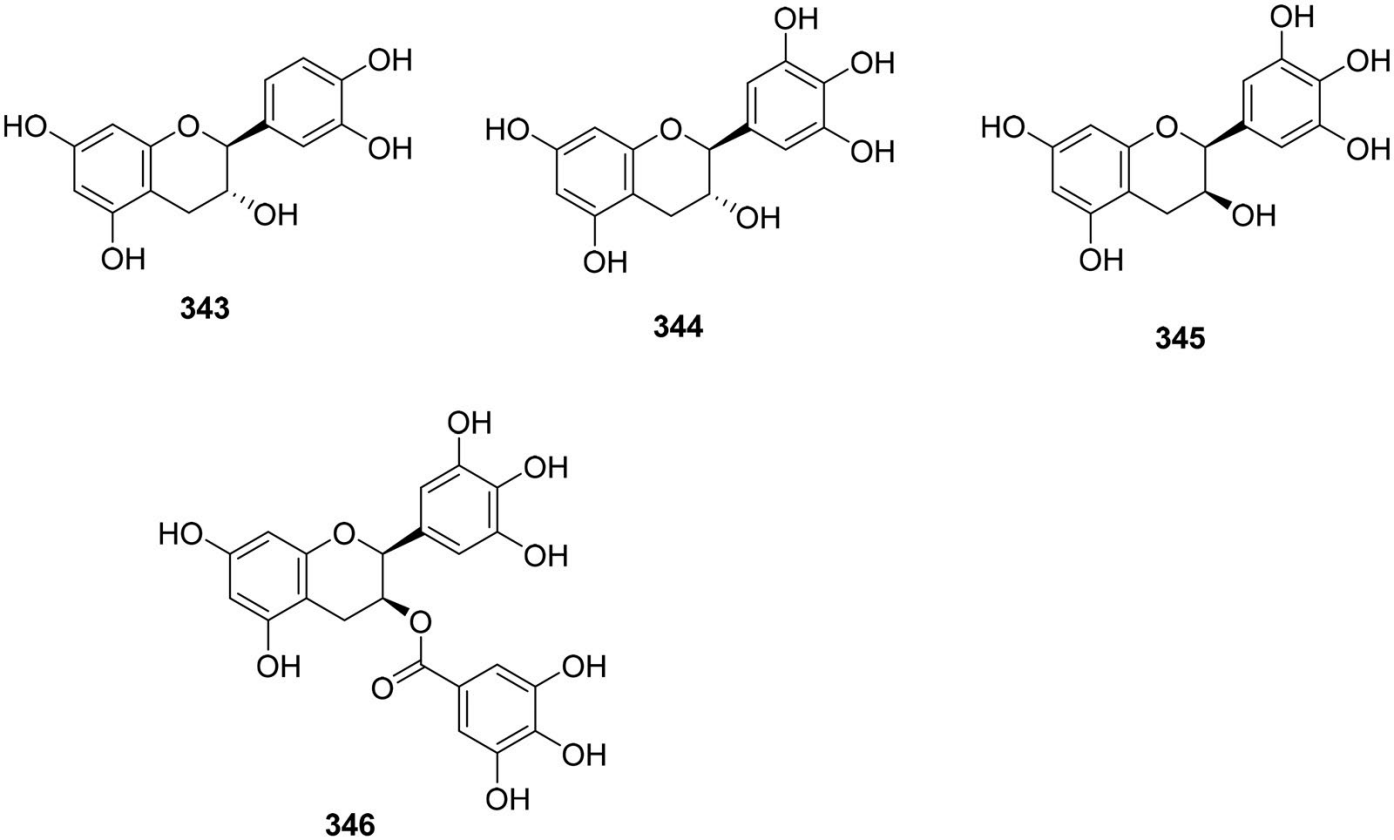
Flavans.

The reduction of flavanones forms antibacterial flavans (Cao et al. 2020) (Figure 31). Examples are catechin (**343**) gallocatechin (**344**) (Pretorius et al. 2003), and epigallocatechin (**345**) (*M. smegmatis*, MIC: 7.8 µg/mL) (Mativandlela et al. 2007). (−)-Epigallocatechin 3-*O*-gallate (EGCG) **(346**) from *Camellia sinensis* (L.) Kuntze (Theaceae, asterids) inhibited the growth of *C. jejuni* (MIC: 8 μg/mL), *N. gonorrhoea* (MIC: 32 μg/mL), *S. pneumoniae* (MIC: 32 μg/mL) (Matsumoto et al. 2012), MDR-*A. baumannii* (MIC: 78 μg/mL, bactericidal) (Osterburg et al. 2009), and *S. maltophilia* (Navarro-Martínez et al. 2005; Gordon and Wareham 2010).

## Antibacterial strength and spectrum of activity

Over the last decades, several MIC threshold values have been proposed to identify natural plant products with very strong antibacterial activity (Fabry et al. 1998; Ríos and Recio 2005), the latest being a MIC ≤ 4 µg/mL (Tankeo and Kuete 2023). Since phenolic compounds often have low therapeutic indices and limited oral bioavailabilities (Serrano et al. 2009; Velderrain-Rodríguez et al. 2014) we recommend reducing this threshold value below 2 µg/mL.

Thus, out of ∼350 antibacterial phenolic compounds identified from 1945 to 2023 in Angiosperms from Asia and the Pacific, 44 are very strongly active (MIC < 2 µg/mL) (Table 1). To the extent that resistance thresholds must be considered for antibiotics, a MIC of <1 µg/mL could be used as a threshold for the possible clinical development of natural products. Plumbagin (**127**), isodiospyrin (**137**), malabaricone A (**164**), malabaricone B (**165**), anacardic acid (**170**), cannabidiol (**174**), bakuchiol (**176**), hypercalin B (**181**), lupulone (**183**), rhodomyrtone (**186**), cowanone (**191**), α-mangostin (**201**), β-mangostin (**202**), garcicowanone A (**206**), 7,9,2′,4′-tetrahydroxy-8-isopentenyl-5-methoxychalcone (**265**), and sophoraflavanone G (**279**) have a MIC < 1 µg/mL against Gram-positive bacteria. A MIC of <1 µg/mL was obtained with 1′-acetoxychavicol acetate (**21**), juglone (**124**), 3-methoxyjuglone (**125**), plumbagin (**127**), and 2-methoxy-7-methyljuglone (**128**) against mycobacteria. Dendrocoumarin (**53**) and cannabidiol (**174**) have a MIC < 1 µg/mL against Gram-negative bacteria.

**Table 1.** Phenolic compounds with very strong antibacterial activity (MIC < 2 µg/mL).

	MM	LogD	PSA	FRB	Planar	G	HA	HD	S	MIC < 1 µg/mL	BS	BC
Non-flavonoids												
Hydroxycinnamic acid derivatives												
2-Methoxy-2-butenolide-3-cinnamate (**15**)	260	–	–	–			–	–	Mb			
Phenylpropanoids												
1′-Acetoxychavicol acetate (**21**)	234.2	2.3	53	6			4	0	Mb	**√**		
Coumarins												
Dendrocoumarin (**53**)	244	–	–	–	**√**		–	–	G−	**√**		
Itolide A (**54**)	244	–	–	–	**√**		–	–	G−**/**G+			
Stilbenes												
Cajanin stilbene acid (**62**)	338.4	5.8	67	6			2	4	G+			
1,4-Naphthoquinones												
Juglone (**124**)	174.1	0.9	54	0			1	3	G+		**√**	
									Mb	**√**		
3-Methoxyjuglone (**125**)	–	–	–	–	**√**		–	–	Mb	**√**		
Plumbagin (**127**)	188.1	1.4	54	0	**√**		3	1	G+**/**Mb	***√***		
2-Methoxy-7-methyl juglone (**128**)	218.2	–	64	1	**√**		4	1	Mb	**√**		
7-Methyljuglone (**129**)	188.1	1.3	54	0	**√**		3	1	Mb		**√**	
Diospyrin (**136**)	372	0.5	109	1	**√**		6	2	Mb		**√**	
Isodiospyrin (**137**)	374	0.5	109	1	**√**		6	2	G+	**√**		
Anthraquinones												
Aloe-emodin (**143**)	270.2	1.2	95	1	**√**		5	3	G−**/**G+			
Rhein (**144**)	284.2	−0.5	112	1	**√**		6	3	G−			
Emodin (**145**)	270.2		1.7	0	**√**		5	3	G+			
Long chain alkyl phenols												
Malabaricone A (**164**)	326.4	5.6	58	10		**√**	3	2	G+	**√**		**√**
Malabaricone B (**165**)	328.4	4.5	78	10		**√**	4	3	G+	**√**		**√**
Anacardic acid (**170**)	348.5	6.4	58	10		**√**	5	4	G+	**√**		
Cannabidiol (**174**)	324.2	6.4	40	6		**√**	2	2	G+**/**G−	**√**		
Cannabigerol (**175**)	316.4	6.7	40	9		**√**	2	2	G+			
Bakuchiol (**176**)	256.3	5.6	20	6		**√**	1	1	G+	**√**		
Prenylated phloroglucinols												
Hypercalin B (**181**)	518.7	–	94.8	8	**√**		5	3	G+	**√**		
Lupulone (**183**)	414.5	3.8	75	9	**√**		4	4	G+	**√**		
Rhodomyrtone (**186**)	442.5	5.3	101	5	**√**	**√**	2	6	G+	**√**		
Isomyrtucommulone B (**187**)	415.2	–	–	–	**√**	**√**	–	–	G+			
Myrciarone B (**188)**	429.2	–	–	–	**√**	**√**	–	–	G+			
Prenylated benzophenones												
Cowanone (**191**)^,^	–	–	–	–		**√**	–	–	G+	**√**		
Prenylated xanthones												
Gerontoxanthone I (**197**)	396.4	3.8	107	4	**√**	**√**	6	4	G−			
9-Hydroxycalabaxanthone (**198**)	426.5	4.4	85	3	**√**	**√**	6	2	G−			
α-Mangostin (**201**)	410.4	4.2	96	5	**√**	**√**	6	3	G+	**√**		
β-Mangostin (**202**)	424.4	4.6	85	6	**√**	**√**	6	2	G+	**√**		
Garcicowanone A (**206**)	356.4	4.7	85.2	4	**√**	**√**	6	2	G+	**√**		
Rubraxanthone (**207**)	410.1	4.1	96	6	**√**	**√**	6	3	G+			
Gerontoxanthone H (**229**)	380.4	4.6	87	4	**√**	**√**	5	3	G+			
Other non-flavonoids												
Gancaonin I (**251**)	354.3	4.1	72	5			5	2	G+			
Δ_9_-tetrahydrocannabinol (**256**)	314.4	7.2	29	4	**√**	**√**	2	1	G+			
Cannabinol (**257**)	310.2	6.7	29	4	**√**	**√**	2	1	G+			
Flavonoids												
Chalcones												
7,9,2′,4′-TIMC (**265**)	–	–	–	–		**√**	–	–	G+	**√**		
Flavanones												
Glabrol	(**273**)	392.4	5.7	67	5	**√**	4	2	G+/Mb			
Sophoraflavanone G (**279**)	424.4	5.3	107	6	**√**	6	4	G+	**√**			
Flavones												
Kaempferol-7-rhamnoside (**317**)	432.3	−0.3	166	3		10	6	G−				
Isoflavones												
Erycristagallin (**339**)	390.4	6.3	63	4	**√**	**√**	4	2	G+			
Glycyrrhizol A (**340**)	420.1	6.3	72	5	**√**	**√**	5	5	G+			
Erybraedin A (**342**)	392.4	6	59	4	**√**	**√**	4	2	G+			

MM: molecular mass (g/mol); LogD: at pH 7.4; PSA: polar surface area (Å²); G: one or more long prenyl or long-chain alkyl group present; FRB: freely rotating bond; H: hydrogen bond acceptor; HD: hydrogen bond donor; S: spectrum; G+: Gram-positive; Mb: mycobacteria; BS: bacteriostatic; BC: bactericidal; –: not available.

## Distribution of phenolic compounds with very strong antibacterial activity

Among those above 44 phenolic compounds, 33 originate from core Angiosperms, including 25 from fabids (Table 2). In the fabids, eight phenolic compounds come from the Fabaceae family, five from the Clusiaceae family, and three from the Hypericaceae family. The other major clades that produce such compounds are the core Angiosperms are the malvids and that fabids (which are sister groups) and the Asterids Fabids and Malvids produce prenylated phenolic compounds. Asterids produce naphthoquinones. No phenolic compounds with very strong antibacterial activity were identified among protomagnoliids, eudicots, rosids, lamiids, and campanulids.

**Table 2.** Distribution of phenolics compound with very strong antibacterial activity (MIC < 2 µg/mL).

	Group	Clade	Order	Family
Non-flavonoids				
Hydroxycinnamic acid derivatives				
2-Methoxy-2-butenolide-3-cinnamate (**15**)	Core angiosperms	Malvids	Caryophyllales	Polygonaceae
Phenylpropanoids				
1′-Acetoxychavicol acetate (**21**)	Basal angiosperms	Monocots	Zingiberales	Zingiberaceae
Coumarins				
Dendrocoumarin (**53**)	Basal angiosperms	Monocots	Asparagales	Orchidaceae
Itolide A (**54**)	Basal angiosperms	Monocots	Asparagales	Orchidaceae
Stilbenes				
Cajanin stilbene acid (**62**)	Core angiosperms	Fabids	Fabales	Fabaceae
1,4-Napthoquinones				
Juglone (**124**)	Core angiosperms	Fabids	Fagales	Juglandaceae
3-Methoxyjuglone (**125**)	Core angiosperms	Fabids	Fagales	Juglandaceae
Plumbagin (**127**)	Upper angiosperms	Asterids	Ericales	Ebenaceae
2-Methoxy-7-methyl juglone (**128**)	Upper angiosperms	Asterids	Ericales	Ebenaceae
7-Methyljuglone (**129**)	Upper angiosperms	Asterids	Ericales	Ebenaceae
Diospyrin (**136**)	Upper angiosperms	Asterids	Ericales	Ebenaceae
Isodiospyrin (**137**)	Upper angiosperms	Asterids	Ericales	Ebenaceae
Anthraquinones				
Aloe-emodin (**143**)	Core angiosperms	Malvids	Caryophyllales	Polygonaceae
Rhein (**144**)	Core angiosperms	Malvids	Caryophyllales	Polygonaceae
Emodin (**145**)	Core angiosperms	Malvids	Caryophyllales	Polygonaceae
Long chain alkyl phenols				
Malabaricone A (**164**)	Basal angiosperms	Magnoliids	Laurales	Myristicaceae
Malabaricone B (**165**)	Basal angiosperms	Magnoliids	Laurales	Myristicaceae
Anacardic acid (**170**)	Core angiosperms	Fabids	Sapindales	Anacardiaceae
Cannabidiol (**174**)	Core angiosperms	Fabids	Rosales	Cannabaceae
Cannabigerol (**175**)	Core angiosperms	Fabids	Rosales	Cannabaceae
Bakuchiol (**176**)	Core angiosperms	Malvids	Caryophyllales	Amaranthaceae
Prenylated phloroglucinols				
Hypercalin B (**181**)	Core angiosperms	Fabids	Malpighiales	Hypericaceae
Lupulone (**183**)	Core angiosperms	Fabids	Rosales	Cannabaceae
Rhodomyrtone (**186**)	Core angiosperms	Malvids	Myrtales	Myrtaceae
Isomyrtucommulone B (**187**)	Core angiosperms	Malvids	Myrtales	Myrtaceae
Myrciarone B (**188**)	Core angiosperms	Malvids	Myrtales	Myrtaceae
Prenylated benzophenones				
Cowanone (**191**)	Core angiosperms	Fabids	Malpighiales	Clusiaceae
Prenylated xanthones				
Gerontoxanthone I (**197**)	Core angiosperms	Fabids	Malpighiales	Hypericaceae
9-Hydroxycalabaxanthone (**198**)	Core angiosperms	Fabids	Malpighiales	Hypericaceae
α-Mangostin (**199**)	Core angiosperms	Fabids	Malpighiales	Clusiaceae
β-Mangostin (**201**)	Core angiosperms	Fabids	Malpighiales	Clusiaceae
Garcicowanone A (**206**)	Core angiosperms	Fabids	Malpighiales	Clusiaceae
Rubraxanthone (**207**)	Core angiosperms	Fabids	Malpighiales	Clusiaceae
Gerontoxanthone H (**229**)	Core angiosperms	Fabids	Rosales	Moraceae
Others non-flavonoids				
Gancaonin I **(251**)	Core angiosperms	Fabids	Fabales	Fabaceae
Δ_9-_Tetrahydrocannabinol (**256**)	Core angiosperms	Fabids	Rosales	Cannabaceae
Cannabinol (**257**)	Core angiosperms	Fabids	Rosales	Cannabaceae
Flavonoids				
Chalcones				
7,9,2′,4′-TIMC (**265**)	Core angiosperms	Fabids	Fabales	Fabaceae
Flavanones				
Glabrol (**274**)	Core angiosperms	Fabids	Fabales	Fabaceae
Sophoraflavanone G (**280**)	Core angiosperms	Fabids	Fabales	Fabaceae
Flavones				
Kaempferol-7-rhamnoside (**318)**	Core angiosperms	Core eudicots	Saxifragales	Crassulaceae
Isoflavones				
Erycristagallin (**340**)	Core angiosperms	Fabids	Fabales	Fabaceae
Glycyrrhizol A (**341**)	Core angiosperms	Fabids	Fabales	Fabaceae
Erybraedin A (**342**)	Core angiosperms	Fabids	Fabales	Fabaceae

## Influence of molecular mass on the antibacterial strength and spectrum of activity

The molecular mass of phenolic compounds can be tentatively classified as follows: <200 g/mol = low, 200–400 g/mol = moderate, and >400 g/mol = high. Consequently, phenolic compounds with very strong activity against Gram-positive bacteria, Gram-negative bacteria, or mycobacteria have on average a low, moderate, or high molecular mass (Table 1). These phenolic compounds have molecular masses ranging from 174.1 to 518.7 g/mol. None of the phenolic compounds with a MIC < 1 µg/mL against Gram-negative bacteria or mycobacteria have high molecular masses, probably due to porins’ molecular mass exclusion limit (Bauer et al. 1988; Niederweis 2003).

## Influence of solubility on the antibacterial strength and spectrum of activity

The solubility of phenolic compounds can be tentatively classified as follows at pH 7.4: LogD <1 = hydrophilic, LogD between 1 and 5 = amphiphilic, and LogD > 5 = lipophilic. Consequently, phenolic compounds with very strong antibacterial activity are on average amphiphilic (Table 1). Phenolic compounds with a MIC < 1 µg/mL against Gram-positive bacteria are lipophilic or nearly lipophilic. Phenolic compounds with a MIC < 1 µg/mL against Gram-negative bacteria are hydrophilic, amphiphilic, or lipophilic. Lipophilic compounds do not pass through the porins of Gram-negative bacteria (Bauer et al. 1988; van den Berg 2010), suggesting a mechanism of action for lipophilic phenolic compounds involving the permeabilization of the outer membrane. Likewise, porins of mycobacteria likely prevent the entry of lipophilic phenolic compounds. The polar surface area of phenolic compounds with very strong antibacterial activity varies from 29 to 166 Å^2^.

## Main mechanisms of action

Most phenolic compounds are bactericidal. Their two main mechanisms of action are the permeabilization of the cytoplasmic membrane and the destruction of the peptidoglycan wall (Table 3). Downstream effects of cytoplasmic membrane permeabilization include respiratory chain inhibition, change in cytoplasmic pH, generation of reactive oxygen species, and ultimately the inhibition of DNA, RNA, and protein synthesis (Booth 1985; Kubo et al. 2003; Yuan et al. 2021). For mycobacteria, the principal mechanism of action is the inhibition of enzymes, such as DNA primase (Gajadeera et al. 2015) or shikimate kinase (Pandey et al. 2016). There does not appear to be a relationship between molecular mass or solubility with a specific bacterial target.

**Table 3.** Mechanism of action.

	MM	LogD	Planar	G	Target	S	References
Non-flavonoids							
Hydroxycinnamic acid derivatives							
Cinnamic acid (**1**)	148.1	−0.6			Membrane	G+	Cai et al. 2019
						G−	Hemaiswarya and Doble 2010
Caffeic acid (**3**)	180.1	−1.7			Membrane	G+	Hemaiswarya and Doble 2010
						G−	Hemaiswarya and Doble 2010
Chlorogenic acid (**4**)	354.3	−3.9			Membrane	G+	Cai et al. 2019
						G−	Hemaiswarya and Doble 2010
Phenylpropanoids							
Eugenol (**22**)	164.2	2.4		√	Membrane	G−	Ashrafudoulla et al. 2020
Coumarins							
Fraxetin (**28**)	208.1	0.2	√		Membrane	G+	Wang et al. 2014
					DNA	G+	Wang et al. 2014
					RNA	G+	Wang et al. 2014
					Topoisomerases	G+	Wang et al. 2014
Stilbenes							
Resveratrol (**56**)	228.2	2.8			DNA	G+	Paulo et al. 2010
Pterostilbene (**58**)	256.2	3.8			DNA	G+	Shih et al. 2021
Cajanin stilbene acid (**62**)	338.4	5.8		√	Phosphotransferase	G+	Tan, Hua, et al. 2020
ɛ-Viniferin (**69**)	454.4	4.6			Membrane	G+	Basri et al. 2014
Dehydro-*δ*-viniferin (**73**)	451.1	–			Membrane	G+	Mattio et al. 2019
Lignans							
(−)-Nortrachelogenin (**85**)	374.3	1.1			Membrane	G−	Lee, Ji, et al. 2016
Glochidioboside (**89**)	522.5	–			Membrane	G−	Lee, Woo, et al. 2015
Magnolol (**90**)	266.3	4.0			Membrane	G+	Liu et al. 2014
Honokiol (**91**)	266.3	4.1			Membrane	G+	Liu et al. 2014
Hydroxybenzoic acid derivatives							
Gallic acid (**99**)	170.1	−2.3			Membrane	G−	Kang et al. 2018
Methyl gallate (**100**)	184.1	1.3			Membrane	G−	Acharyya et al. 2015
Ethyl gallate (**102**)	198.1	1.4			Peptidoglycan	G−	Li, Song, et al. 2016
Miscellaneous simple phenolic compounds							
Hydroquinone (**108**)	110.1	0.5			Membrane	G+	Jeyanthi et al. 2021
					Membrane	G0	Jeyanthi et al. 2021
					Peptidoglycan	G−	Ma et al. 2019
Thymol (**109**)	150.2	3.0			Membrane	G−	Xu et al. 2008
					Membrane	G+	Yuan et al. 2018
Ellagic acid (**113**)	302.1	−2.0	**√**		DNA gyrase	G−	Ohemeng et al. 1993
Benzoquinones							
Thymoquinone (**118**)	164.2	1.9			ATP synthase	G−	Ahmad et al. 2015
1,4-Naphthoquinones							
Plumbagin (**127**)	188.1	1.4	**√**		DNA	G−	Farr et al. 1985
					Membrane	G−	Wang, Kong, et al. 2022
					Peptidoglycan	G+	Periasamy et al. 2019
7-Methyljuglone (**129)**	188.1	1.3	**√**		Mycothiol disulfide reductase	Mb	Mahapatra et al. 2007
Shikonin (**139**)	288.2	3.2	**√**	√	Membrane	G+	Lee, Lee, et al. 2015
					Peptidoglycan	G+	Lee, Lee, et al. 2015
					ATPase	G+	Lee, Lee, et al. 2015
					Topoisomerases		Plyta et al. 1998
Anthraquinones							
Aloe-emodin (**143**)	270.2	1.7	**√**		DNA primase	Mb	Gajadeera et al. 2015
					Membrane	G+	Li et al. 2021
Emodin (**145**)	270.2	1.7	**√**		Membrane	G−	Gajadeera et al. 2015
					DNA	G−	Zhang et al. 2020
Tannins							
Tannic acid (**158**)	1701.1	3.1			Peptidoglycan	G+	Dong et al. 2018
Corilagin (**159)**	634.4	0.9			Membrane	G−	Li et al. 2013
Punicalagin (**162**)	1084.7	−4.4			Membrane	G−	Li et al. 2020
ZP-CT-A	–	–			Membrane	G+	Kusuda et al. 2006
Long chain alkyl phenols							
Malabaricone A (**164**)	328.4	4.5		**√**	Membrane	G+	Sivadas et al. 2023
Malabaricone B (**165**)	326.4	5.6		**√**	Membrane	G+	Sivadas et al. 2023
Anacardic acid (**170**)	348.5	6.4		**√**	Membrane	G+	Kubo et al. 2003
Cannabidiol (**174**)	324.2	6.4		**√**	Membrane	G+	Blaskovich et al. 2021
Cannabigerol (**175**)	316.4	6.7		**√**	Membrane	G+	Aqawi et al. 2021
Prenylated phloroglucinols							
Rottlerin (**182**)	516.5	5.8		**√**	Shikimate kinase	Mb	Pandey et al. 2016
Rhodomyrtone (**186**)	442.5	5.3	**√**	**√**	Membrane	G+	Saeloh et al. 2018
					Peptidoglycan	G+	Saeloh et al. 2018
Callistemenonone A (**189**)	461.1	–	**√**	**√**	Membrane	G+	Xiang et al. 2017
					DNA	G+	Saeloh et al. 2018
Prenylated xanthones							
Cochinchinone A (**196**)	448.5	6.9	**√**	**√**	Membrane	G−	Boonnak et al. 2009
α-Mangostin (**201**)	410.4	4.2	**√**	**√**	Respiratory chain	G+	Nguyen and Marquis 2011
					Membrane	G+	Sivaranjani et al. 2019
Chromanes and chromenes							
Brasilin (**231**)	286.2	1.6	**√**		DNA	G+	Xu and Lee 2004
Naphthalenols							
Hibicuslide C (**237**)	232.3	–	**√**		DNA	G−	Lee, Choi, et al. 2016
Phenanthrenes							
Blestriacin (**241**)	–	–	**√**		Membrane	G+	Chen et al. 2018
4, 8, 4′, 8′-TBT (**243**)	–	–	**√**		Membrane	G+	Huang et al. 2021
Blestiarene A (**244**)	–	–	**√**		Membrane	G+	Zhang et al. 2022
Densiflorol B (**245**)	–		**√**		Membrane	G+	Zhang et al. 2022
Other non-flavonoids							
Hyperenone A (**254**)	271.1	–		**√**	MurE ligase	Mb	Osman et al. 2010
Flavonoids							
Chalcones							
Licochalcone A (**262**)	338.3	4.3		**√**	Respiratory chain	G+	Haraguchi et al. 1998
Flavanones							
Artocarpanone (**267**)	302.2	2.3			Membrane	G−	Septama and Panichayupakaranant 2017
Naringenin (**269**)	272.2	2.2			Membrane	G+	Tsuchiya and Iinuma 2000
Glabrol (**273**)	392.4	5.7		**√**	Membrane	G+	Wu et al. 2019
Lupinifolin (**276**)	406.4	5.8		**√**	Membrane	G+	Yusook et al. 2017
Sophoraflavanone G (**279**)	424.4	5.3		**√**	Membrane	G+	Tsuchiya and Iinuma 2000
					Peptidoglycan	G+	Kim and Kim 2020
Isoflavanones							
Bidwillon B (**295**)	–	–	**√**	**√**	DNA	G+	Sato et al. 2003
Flavones							
Galangin (**299**)	270.2	1.5	**√**		Membrane	G+	Cushnie and Lamb 2005
					DNA helicase	G−	Chen and Huang 2011
Luteolin (**301**)	286.2	1.1	**√**		Membrane	G+	Siriwong et al. 2015
					Peptidoglycan	G+	Siriwong et al. 2015
					DNA	G+	Siriwong et al. 2015
Quercetin (**303**)	302.2	2.1	**√**		Membrane	G+	Siriwong et al. 2015
					Peptidoglycan	G+	Siriwong et al. 2015
					DNA	G+	Siriwong et al. 2015
Kuwanon G (**312**)	692.2	5.1	√	**√**	Membrane	G+	Park et al. 2003
Artocarpin (**313**)	436.3	4.5	√	**√**	Membrane	G+	Septama and Panichayupakaranant 2018
					Membrane	G−	Septama et al. 2022
Isoflavones							
Tectorigenin (**327**)	300.2	1.5	√		Membrane	G+	Joung et al. 2014
					ATP-binding cassette	G+	Joung et al. 2014
					Peptidoglycan	G+	Joung et al. 2014
Glycinol (**336**)	272.2	1.4	√		Membrane	G−	Weinstein and Albersheim 1983
					DNA	G−	Weinstein and Albersheim 1983
Isoflavans							
Erycristagallin (**339**)	390.4	6.3	**√**	**√**	Membrane	G+	Sato et al. 2003
					DNA	G+	Sato et al. 2003
Flavans							
EGCG (**346**)	458.3	1.6			Membrane	G−	Cao et al. 2019
					DNA gyrase	G+	Gradišar et al. 2007
					DNA gyrase	G−	Gradišar et al. 2007
					Dihydrofolate reductase	G−	Navarro-Martínez et al. 2005

MM: molecular mass (g/mol); LogD: at pH 7.4; G: prenyl or long-alkyl chain group; –: non-available; S: spectrum; G+: gram-positive; G−: gram-negative; Mb: mycobacteria.

## Structure-activity

### General observations

Phenolic compounds with a MIC < 2 µg/mL are, for the most part, not flavonoids (Table 1). These mainly include long-chain alkyl phenols, prenylated phloroglucinols, prenylated xanthones, and 1,4-naphthoquinones. Dimethylallyl, geranyl, farnesyl, lavandulyl, and long-chain alkyl groups tend to penetrate and remain in the hydrophobic region of the cytoplasmic membrane and, in doing so, render it permeable (Tsukiyama et al. 2002; Appendino et al. 2008; Kim et al. 2014; Omosa et al. 2016; Araya-Cloutier et al. 2018; Yuan et al. 2021) (Table 3). In addition, when these groups make phenolic compounds amphiphilic, they become surfactants (Xia et al. 1995). Phenolic compounds that cross the porins of Gram-negative bacteria (hydrophilic or close to hydrophilic) reach the cytoplasmic membrane to destabilize it.

This is probably the case for (−)-nortrachelogenin (**85**) (Lee, Ji, et al. 2016) and glochidioboside (**89**) with *E. coli* O157:H7 (Lee, Woo, et al. 2015). A restricted number of freely rotating chemical bonds and the presence of a planar skeleton (as in anthraquinones or naphthoquinones) favor the intercalation of phenolic compounds within bacterial DNA (Table 3) (Bhakta and Siva 2012). Most phenolic compounds with a MIC < 1 µg/mL against mycobacteria are 1,4-naphthoquinones.

### Non-flavonoid phenolic compounds

The presence of quinone scaffolds and an increase in hydrogen bond acceptors enhance the activity of non-flavonoid phenolic compounds (Table 1). Quinones, through the redox cycle (Lown 1983) and in the presence of Cu^2+^ and O_2_, generate reactive oxygen species that destroy bacterial DNA (Sakihama et al. 2002). This is the case for plumbagin (**127**) in *E. coli* (Farr et al. 1985). Naphthoquinones and other planar phenolic compounds with adjacent hydroxyl and ketone groups chelate Zn^2+^ ions and, in doing so, inhibit zinc metalloenzymes, such as topoisomerase I (Tse-Dinh and Beran-Steed 1988; Plyta et al. 1998; Tesauro et al. 2010). Catechol groups in tannins and other polyhydroxylated phenolic compounds chelate Fe^3+^ ions which are necessary for bacterial growth (Serrano et al. 2009; Farha et al. 2020). Similarly, pyrogallol groups chelate divalent cations that are essential for the stabilization of the negative charges in the central oligosaccharide chains of the outer layer of Gram-negative bacteria (Denyer and Maillard 2002; Taguri et al. 2006).

Certain functional groups readily and randomly form cross-links with bacterial macromolecules. For example, furan and pyrone moieties in furanocoumarins form covalent bonds with DNA and proteins (Dall’Acqua et al. 1978; Bordin et al. 1993; Wamer et al. 1995). Similarly, α, β-unsaturated carbonyls in butenolides readily open to form covalent bonds with the nucleophilic groups (thiols) of proteins *via* hetero-Michael addition reactions (Jackson et al. 2017). Benzoquinones form covalent bonds with proteins (Wipf and Jung 1999; Hettegger et al. 2021). Other examples include α-methylene γ-lactones that alkylate DNA and proteins (Gach and Janecka 2014). Tannins randomly interact with surface proteins and the cytoplasmic membrane (Shimozu et al. 2017; Tintino et al. 2017; Wang et al. 2020) and at high concentrations coagulate proteins (Serrano et al. 2009). Thus, they often behave as invalid metabolic panaceas.

Another mechanism of action is the induction of apoptosis as in pterostilbene (**58**) with *B. cereus* (Shih et al. 2021) and quercetin (**303**) (Li et al. 2015). None of these compounds are known so far to interact with outer membrane protein A (OmpA) in *A. baumannii* (Nie et al. 2020).

### Flavonoids

Prenyl groups enhance the activity of flavonoids against Gram-positive bacteria (Matsumoto et al. 2012; Yuan et al. 2021), as in 7,9,2′,4′-tetrahydroxy-8-isopentenyl-5-methoxychalcone (**265**) and sophoraflavanone G (**279**) (Table 1). Sophoraflavanone G (**279**) is a surfactant that induced the lysis of *E. faecium* (Tsuchiya and Iinuma 2000; Kim and Kim 2020). Flavonoid glycosides are able to pass through porins and can therefore dissolve the cytoplasmic membrane of Gram-negative bacteria (Tagousop et al. 2018). This could explain why the only flavonoid with a MIC < 2 against a Gram-negative bacterium is kaempferol-7-rhamnoside (**317**). No flavonoid showed very strong activity against mycobacteria.

Regarding the influence of the number and the position of hydroxyl groups on the antibacterial mechanism of flavonoids, a hydroxyl group at carbon 3 of ring A and hydroxylation of ring B at carbons 3′, 4′, or 5′ promote the intercalation of flavonoids into bacterial DNA (Bartoszewski and Króliczewski 2019). It has also been observed that a hydroxyl group at carbon 3 (Hazni et al. 2008), 5, 6, or 7 enhance antibacterial activity (Hummelova et al. 2015). However, an increasing number of hydroxyl groups results in a decrease in activity (Mori et al. 1987; Xu et al. 2015). Flavonoids with catechol scaffolds or hydroxyl groups at carbon 3 or 5 and a ketone group at position 4 can chelate Fe^3+^ ions (Porfírio et al. 2014; Jahanshahi et al. 2022).

## Synergistic activity with antibiotics

### Weakening of intrinsic resistance

Phenolic compounds are primarily synergistic with β-lactam antibiotics against Gram-positive bacteria and, to a lesser extent, with aminoglycosides, tetracyclines, and fluoroquinolones (Table 4). There is no obvious association between the molecular mass or solubility of phenolic compounds and synergistic effects with specific classes of antibiotics. However, there appears to be a link between mechanism of action and synergy with a given class of antibiotic. For example, phenolic compounds targeting the cytoplasmic membrane or the peptidoglycan wall often work synergistically with antibiotics targeting the cytoplasmic membrane or the peptidoglycan wall (Table 4). The synergistic mechanism of action phenolic compounds with prenyl or long-chain alkyl groups relies, at least in part, on the destabilization of the cytoplasmic membrane (Tsukiyama et al. 2002; Appendino et al. 2008; Omosa et al. 2016; Zabawa et al. 2016).

**Table 4.** Synergy with antibiotics.

	MM	LogD	Class of antibiotic	Target	Antibiotic	Bacteria	References
Non-flavonoids							
Hydroxycinnamic acid derivatives							
Cinnamic acid (**1**)	148.1	−0.6	Aminoglycoside	Ribosomes	Amikacin	*S. aureus*	Hemaiswarya and Doble 2010
			Aminoglycoside	Ribosomes	Amikacin	*E. coli*	Hemaiswarya and Doble 2010
			β-Lactam	Peptidoglycan	Ampicillin	*S. aureus*	Hemaiswarya and Doble 2010
			β-Lactam	Peptidoglycan	Ampicillin	*E. coli*	Hemaiswarya and Doble 2010
			β-Lactam	Peptidoglycan	Ciprofloxacin	*S. aureus*	Hemaiswarya and Doble 2010
			β-Lactam	Peptidoglycan	Ciprofloxacin	*E. coli*	Hemaiswarya and Doble 2010
			β-Lactam	Peptidoglycan	Ciprofloxacin	*P. aeruginosa*	Hemaiswarya and Doble 2010
			Macrolide	Ribosomes	Erythromycin	*S. aureus*	Hemaiswarya and Doble 2010
			Macrolide	Ribosomes	Erythromycin	*E. coli*	Hemaiswarya and Doble 2010
			Glycopeptide	Ribosomes	Vancomycin	*S. aureus*	Hemaiswarya and Doble 2010
			Glycopeptide	Peptidoglycan	Vancomycin	*E. coli*	Hemaiswarya and Doble 2010
Caffeic acid (**3**)	180.1	−1.7	Fluoroquinolone	DNA	Norfloxacin	*S. aureus*	Lima et al. 2016
			β-Lactam	Peptidoglycan	Imipenem	*E. coli*	Lima et al. 2016
Chlorogenic acid (**4**)	354.3	−3.9	Fluoroquinolone	DNA	Levofloxacin	*K. pneumoniae*	Tan, Gao, et al. 2020
			Macrolide	Ribosomes	Erythromycin	*S. aureus*	Kępa et al. 2018
			Licosamide	Ribosomes	Clindamycin	*S. aureus*	Kępa et al. 2018
			β-Lactam	Peptidoglycan	Cefoxitin	*S. aureus*	Kępa et al. 2018
Phenylpropanoids							
Eugenol (**22**)	164.2	2.4	Aminoglycoside	Ribosomes	Streptomycin	*L. monocytogenes*	Liu et al. 2015
			Aminoglycoside	Ribosomes	Streptomycin	*S. typhimuriim*	Liu et al. 2015
Coumarins							
Isoimperatorin (**32**)	270.2	–	Ansamycin	DNA	Rifampicin	*M. tuberculosis*	Guo et al. 2014
			Ethylenediamine	Wall	Ethambutol	*M. tuberculosis*	Guo et al. 2014
			Hydrazide	Membrane	Isoniazid	*M. tuberculosis*	Guo et al. 2014
Stilbenes							
Resveratrol (**56**)	228.2	2.8	Polypeptide	Membrane	Polymixin B	*E. coli*	Liu et al. 2020
			Polypeptide	Membrane	Polymixin B	*K. pneumoniae*	Liu et al. 2020
Pterostilbene (**58**)	256.2	3.8	β-Lactam	Peptidoglycan	Oxacillin	MRSA	Ishak et al. 2016
			Aminoglycoside	Ribosomes	Gentamycin	*S. aureus*	Lee et al. 2017
			Aminoglycoside	Ribosomes	Gentamycin	*P. aeruginosa*	Lee et al. 2017
			Aminoglycoside	Ribosomes	Gentamycin	*E. coli*	Lee et al. 2017
Cajanin stilbene acid (**62**)	338.4	5.8	Polypeptide	Membrane	Colistin	*E. coli*	Jia et al. 2023
ɛ-Viniferin (**69**)	454.4	4.6	Glycopeptide	Peptidoglycan	Vancomycin	MRSA	Basri et al. 2014
Lignans							
Magnolol (**90**)	266.3	4.0	Aminoglycoside	Ribosomes	Amikacin	MRSA	Zuo et al. 2015
			Fluoroquinolone	DNA	Levofloxacin	MRSA	Zuo et al. 2015
			Phosphonic	Peptidoglycan	Fosfomycin	MRSA	Zuo et al. 2015
			β-Lactam	Peptidoglycan	Piperacillin	MRSA	Zuo et al. 2015
Honokiol (**91**)	266.3	4.1	Aminoglycoside	Ribosomes	Amikacin	MRSA	Zuo et al. 2015
			Fluoroquinolone	DNA	Levofloxacin	MRSA	Zuo et al. 2015
			Phosphonic	Peptidoglycan	Fosfomycin	MRSA	Zuo et al. 2015
			β-Lactam	Peptidoglycan	Piperacillin	MRSA	Zuo et al. 2015
Hydroxybenzoic acid derivatives							
Gallic acid (**99**)	170.1	−2.3	Sulfonamide	Folic acid	Sulfamethoxazole	*P. aeruginosa*	Jayaraman et al. 2010
			Tetracycline	Ribosomes	Tetracycline	*P. aeruginosa*	Jayaraman et al. 2010
Methyl gallate (**100**)	184.1	1.3	Fluoroquinolone	DNA	Ciprofloxacin	*Salmonella* sp.	Choi et al. 2008
Protocatechuic acid (**104**)	154.1	−1.8	Sulfonamide	Folic acid	Sulfamethoxazole	*P. aeruginosa*	Jayaraman et al. 2010
Miscellaneous simple phenolic compounds							
Thymol (**109**)	150.2	3.0	Aminoglycoside	Ribosomes	Streptomycin	*L. monocytogenes*	Liu et al. 2015
Ellagic acid (**113**)	302.1	−2.0	Tetracycline	Ribosomes	Tetracycline	*E. coli*	Jenic et al. 2021
			Aminocoumarin	DNA	Novobiocin	*A. baumannii*	Chusri et al. 2009
			Steroid	Ribosome	Fusidic acid	*A. baumannii*	Chusri et al. 2009
			Ansamycin	DNA	Rifampicin	*A. baumannii*	Chusri et al. 2009
Cinnamaldehyde (**116**)	132.1	1.7	Aminoglycoside	Ribosomes	Streptomycin	*L. monocytogenes*	Liu et al. 2015
			Aminoglycoside	Ribosomes	Streptomycin	*S. typhimurium*	Liu et al. 2015
			Aminoglycoside	Ribosomes	Streptomycin	*S. typhimurium*	Liu et al. 2015
Benzoquinones							
Thymoquinone (**118**)	164.2	1.9	Aminoglycoside	Ribosomes	Gentamicin	*S. epidermidis*	Liu et al. 2015
			β-Lactam	Peptidoglycan	Penicillin	*S. epidermidis*	Dera et al. 2021
			Fluoroquinolone	DNA	Ofloxacin	*K. pneumoniae*	Dera et al. 2021
			Tetracycline	Ribosomes	Tetracycline	*K. pneumoniae*	Dera et al. 2021
			β-Lactam	Peptidoglycan	Penicillin	*K. pneumoniae*	Dera et al. 2021
			Quinolone	DNA	Nalidixic acid	*K. pneumoniae*	Dera et al. 2021
1,4-Naphthoquinones							
Plumbagin (**124**)	188.1	1.4	Aminoglycoside	Ribosomes	Gentamycin	*K. pneumoniae*	Chen et al. 2020
			β-Lactam	Peptidoglycan	Oxacillin	*S. aureus*	Rondevaldova et al. 2015
			polypeptide	Membrane	Colistin	*P. aeruginosa*	Wang, Wang, et al. 2022
Lawsone methyl ether (**126**)	188.1	1.6	β-Lactam	Peptidoglycan	Ampicillin	MRSA	Meah et al. 2020
7-Methyljuglone (**129**)	188.1	1.3	Hydrazide	Membrane	Isoniazid	*M. tuberculosis*	Bapela et al. 2006
			Ansamycin	DNA	Rifampicin	*M. tuberculosis*	Bapela et al. 2006
Shikonin (**139**)	288.2	3.2	Aminoglycoside	Ribosomes	Gentamycin	MRSA	Li et al. 2022
			β-Lactam	Peptidoglycan	Amoxicillin	MRSA	Li et al. 2022
			Aminoglycoside	Ribosome	Amikacin	MRSA	Li et al. 2022
Anthraquinones							
Rhein (**144**)	284.2	−0.5	β-Lactam	Peptidoglycan	Amoxicillin	MRSA	Joung et al. 2012
			β-Lactam	Peptidoglycan	Ampicillin	MRSA	Joung et al. 2012
			Nitroimidazole	DNA	Metronidazole	*P. gingivalis*	Azelmat et al. 2015
Emodin (**145**)	270.2	1.7	β-Lactam	Peptidoglycan	Ampicillin	MRSA	Lee, Kang, et al. 2010
			β-Lactam	Peptidoglycan	Oxacillin	MRSA	Lee, Kang, et al. 2010
Tannins							
Theasinensin A (**155**)	914.7	2.8	β-Lactam	Peptidoglycan	Oxacillin	MRSA	Hatano et al. 2003
			β-Lactam	Peptidoglycan	Penicillin G	MRSA	Hatano et al. 2003
			β-Lactam	Peptidoglycan	Ampicillin	MRSA	Hatano et al. 2003
			Aminoglycoside	Ribosomes	Streptomycin	MRSA	Hatano et al. 2003
Corilagin (**159**)	634.4	0.9	β-Lactam	Peptidoglycan	Oxacillin	MRSA	Shimizu et al. 2001
Tannic acid (**158**)	1701.1	3.1	Aminocoumarin	DNA	Novobiocin	*A. baumannii*	Chusri et al. 2009
			Steroid	Ribosomes	Fusidic acid	*A. baumannii*	Chusri et al. 2009
			Ansamycin	DNA	Rifampicin	*A. baumannii*	Chusri et al. 2009
Punicalagin (**162**)	1084.7	−4.4	β-Lactam	Peptidoglycan	Oxacillin	MRSA	Mun et al. 2018
ZP-CT-A	–	–	β-Lactam	Peptidoglycan	Oxacillin	MRSA	Kusuda et al. 2006
			β-Lactam	Peptidoglycan	Penicillin G	MRSA	Kusuda et al. 2006
			β-Lactam	Peptidoglycan	Ampicillin	MRSA	Kusuda et al. 2006
			β-Lactam	Peptidoglycan	Cefmetazole	MRSA	Kusuda et al. 2006
Long chain alkyl phenols							
Malabaricone B (**165)**	328.4	4.5	Aminoglycoside	Ribosomes	Gentamycin	*S. aureus*	Sivadas et al. 2023
			Polypeptide	Membrane	Daptomycin	*S. aureus*	Sivadas et al. 2023
Anacardic acid (**170**)	348.5	6.4	β-Lactam	Peptidoglycan	Methicillin	MRSA	Muroi and Kubo 1996
Cannabidiol (**174**)	324.2	6.4	Polypeptide	Membrane	Polymixin B	*A. baumannii*	Hussein et al. 2022
			Polypeptide	Membrane	Bacitracin	*S. aureus*	Wassmann et al. 2020
			β-Lactam	peptidoglycan	Ampicillin	Gram-*S. typhimurium*	Gildea et al. 2022
			Polypeptide	Membrane	Polymyxin B	*S. typhimurium*	Gildea et al. 2022
Prenylated xanthones							
Isojacareubin (**195**)	328.3	2.8	β-Lactam	Peptidoglycan	Ampicillin	MRSA	Zuo et al. 2012
			β-Lactam	Peptidoglycan	Ceftazidime	MRSA	Zuo et al. 2012
			Fluoroquinolone	DNA	Levofloxacin	MRSA	Zuo et al. 2012
α-Mangostin (**201**)	410.4	4.2	Tetracycline	Ribosomes	Tetracycline	*S. aureus*	Ahmad et al. 2019
			Macrolide	Ribosomes	Erythromycin	*S. aureus*	Ahmad et al. 2019
			Licosamide	Ribosomes	Clindamycin	*S. aureus*	Ahmad et al. 2019
			Aminoglycoside	Ribosomes	Gentamicin	VRE	Sakagami et al. 2005
			Glycopeptide	Peptidoglycan	Vancomycin	MRSA	Sakagami et al. 2005
γ-Mangostin (**204**)	396.4	3.8	β-Lactam	Peptidoglycan	Penicillin G	L. interrogans	Seesom et al. 2013
Naphthalenols							
Hibicuslide C (**237**)	232.3	–	Ansamycin	DNA	Rifampicin	*P. aeruginosa*	Lee, Choi, et al. 2016
			Fluoroquinolone	DNA	–	*P. aeruginosa*	Lee, Choi, et al. 2016
Phenanthrenes							
4,8,4′,8′-TBT (**243**)	–	–	Glycopeptide	Peptidoglycan	Vancomycin	*S. aureus*	Huang et al. 2021
			Macrolide	Ribosomes	Erythromycin	*S. aureus*	Huang et al. 2021
Flavonoids							
Chalcones							
Licochalcone A (**262**)	338.3	4.3	Nitroimidazole	DNA	Metronidazole	*P. gingivalis*	Azelmat et al. 2015
7,9,2′,4′-TIMC (**265**)	–	–	β-Lactam	Peptidoglycan	Gentamicin	MRSA	Lee, Kim, et al. 2010
			β-Lactam	Peptidoglycan	Ampicillin	MRSA	Lee, Kim, et al. 2010
			β-Lactam	Peptidoglycan	Ampicillin	VRE	Lee, Kim, et al. 2010
			Licosamide	Ribosome	Clindamycin	VRE	Lee, Kim, et al. 2010
Flavanones							
Artocarpanone (**267**)	302.2	2.3	Fluoroquinolone	DNA	Norfloxacin	MRSA	Septama et al. 2017
Lupinifolin (**276**)	406.4	5.8	β-Lactam	Peptidoglycan	Ampicillin	MRSA	Rattanakiat et al. 2021
			β-Lactam	Peptidoglycan	Cloxacillin	MRSA	Rattanakiat et al. 2021
Sophoraflavanone G (**279**)	424.4	5.3	β-Lactam	Peptidoglycan	Ampicillin	*S. mutans*	Cha et al. 2007
			Fluoroquinolone	DNA	Norfloxacin	MRSA	Sun et al. 2020
			Phosphonic	Peptigoglycan	Fosfomycin	MRSA	Sakagami et al. 1998
			Glycopeptide	Peptidoglycan	Vancomycin	MRSA	Sakagami et al. 1998
Isoflavans							
Glabridin (**284**)	324.3	4.3	Nitroimidazole	DNA	Metronidazole	*P. gingivalis*	Azelmat et al. 2015
Bidwillon B (**295**)	–	–	Monoxycarbolic acid	Ribosomes	Mupirocin	MRSA	Sato et al. 2003
Isoflavanones							
Eryzerin C (**296**)	422.5	–	Glycopeptide	Peptidoglycan	Vancomycin	VRE	Sato et al. 2004
Flavones							
Baicalein (**298**)	270.2	1.6	β-Lactam	Peptidoglycan	Ampicillin	*S. suis*	Lu et al. 2021
Galangin (**299**)	270.2	1.5	β-Lactam	Peptidoglycan	Ceftazidime	*S. aureus*	Eumkeb et al. 2010
			β-Lactam	Peptidoglycan	Ampicillin	MRSA	Lu et al. 2021
Luteolin (**301**)	286.2	1.1	β-Lactam	Peptidoglycan	Ceftazidime	*S. pyogenes*	Siriwong et al. 2015
Quercetin (**303**)	302.2	2.1	β-Lactam	Peptidoglycan	Ceftazidime	*S. pyogenes*	Siriwong et al. 2015
Myricetin (**305**)	318.2	1.5	Sulfonamide	Folic acid	Sulfamethoxazole	*P. aeruginosa*	Jayaraman et al. 2010
			β-Lactam	Peptidoglycan	Oxacillin	MRSA	Pinto et al. 2020
			Nitroimidazole	DNA	Metronidazole	*P. gingivalis*	Azelmat et al. 2015
Morusin (**310**)	420.2	4.7	β-Lactam	Peptidoglycan	Oxacillin	MRSA	Aelenei et al. 2020
Kuwanon G (**312**)	692.2	5.1	β-Lactam	Peptidoglycan	Oxacillin	MRSA	Aelenei et al. 2020
Artocarpin (**313**)	436.3	4.5	Tetracycline	Ribosome	Tetracycline	*P. aeruginosa*	Septama et al. 2022
TLRP (**320**)	450.4	–	Fluoroquinolone	DNA	Levofloxacin	MRSA	An et al. 2011
			β-Lactam	Peptidoglycan	Ceftazidime	MRSA	An et al. 2011
Isoflavones							
Biochanin A (**324**)	284.2	1.9	Fluoroquinolone	DNA	Ciprofloxacin	*S. aureus*	Liu et al. 2011
Erybraedin A (**342**)	392.4	6.0	Glycopeptide	Peptidoglycan	Vancomycin	VRE	Sato et al. 2004
Flavans							
EGCG (**346**)	458.3	1.6	Sulfonamide	Folic acid	Sulfamethoxazole	*S. maltophilia*	Navarro-Martínez et al. 2005

–: non-available; LogD: at pH 7.4.

Antibiotics whose activity is increased against Gram-negative bacteria in the presence of phenolic products are often those that target the synthesis of ribosomes, DNA, or folic acid metabolism, as well as the lipopolysaccharide envelope. For example, polymyxin B, colistin, and other antibiotics whose functioning is based on the permeabilization of the outer envelope of lipopolysaccharides allow the penetration of phenolic compounds (amphiphilic or lipophilic) previously unable to cross porins. This mechanism could, at least in part, explain the synergy observed between polymyxin B and cajanin stilbene acid (**62**) in mice against *E. coli* (Jia et al. 2023) and polymyxin B and cannabidiol (**174**) against polymyxin B-resistant *A. baumannii* (Hussein et al. 2022). In addition, pyrogallol form complexes with divalent cations which are essential for the stabilization of the outer lipopolysaccharides coat of Gram-negative bacteria (Denyer and Maillard 2002; Taguri et al. 2006) and thus reduce the resistance of Gram-negative bacteria to antibiotics as in the case of ellagic acid (**113**) and tannic acid (**158**) (Andjelković et al. 2006; Jayaraman et al. 2010). Tannins, such as corilagin (**159**), work synergistically with β-lactam antibiotics against Gram-positive bacteria (Shimizu et al. 2001).

Phenolic compounds are not only synergistic with antibiotics but also among each other. The question then arises as to whether the synergy of phenolic compounds would constitute an additional strategy developed by plants to keep phytopathogenic bacteria in check. For example, rhein (**144**) is synergistic with licochalcone A (**262**), glabridin (**284**), or myricetin (**305**) against *P. gingivalis* (Azelmat et al. 2015).

### Efflux pumps

Gram-negative bacteria resist antibacterial phenolic compounds by means of efflux pumps (Kuete et al. 2010). Several phenolic compounds inhibit these pumps, such as gallic acid (**99**) and ellagic acid (**113**) with TetK in *S. aureus* (Macêdo et al. 2022), juglone (**124**) (Zmantar et al. 2016), and tannic acid (**158**) (Tintino et al. 2017). Ellagic acid (**113**) and tannic acid (**158**) inhibited efflux pumps in *A. baumannii* (Chusri et al. 2009). It appears that prenyl groups particularly farnesyl and lavandulyl groups, can bind to and inhibit voltage-gated Ca^2+^ channels (De Loof and Schoofs 2019). The resulting decrease in the concentration of Ca^2+^ ions in the cytoplasm leads to the inhibition of Ca^2+^-dependent efflux pumps (Nava et al. 2020). This mechanism likely explains why sophoraflavanone G (**279**) inhibited NorA in MRSA (Sun et al. 2020). Some molecules used therapeutically that act on human neuroreceptors, such as reserpine are *in vitro* capable to inhibit bacterial efflux pumps (Piddock et al. 2010; Sridevi et al. 2017; Saber and Kandala 2018). It raises the question of whether neuroactive phenolic products would be a potential group of pump inhibitors.

### β-Lactamases

Benzoic acid derivatives (Jiamboonsri et al. 2023), proanthocyanidin oligomers (Kusuda et al. 2006), and flavones tend to inhibit β-lactamases (Siriwong et al. 2015).

### Escaping the development resistance

Antibacterial phenolic compounds that target the cytoplasmic membrane of Gram-positive bacteria evade bacterial resistance. This the case for instance of brasilin (**231**) with MRSA (Xu and Lee 2004), sophoraflavanone G (**279**) with MRSA (Weng et al. 2023), blestriacin (**241**) with *S. aureus* (Chen et al. 2018), malabaricone B (**165**) with *S. aureus* (Sivadas et al. 2023), glabrol (**273**) with MRSA (Wu et al. 2019), as well as cannabidiol (**174**) with MRSA (Blaskovich et al. 2021).

## Toxicity

Unlike traditional antibiotics which come from bacteria or fungi, phenolic compounds from Angiosperms have the advantage of not having specific bacterial targets. It allows them to avoid the development of resistance but gives them a generally narrow therapeutic window. Most phenolic compounds have the disadvantage of often being toxic *in vitro* for mammalian cells at concentrations close to or similar to their MICs. This is the case for xanthones (Boonsri et al. 2006; Mahabusarakam et al. 2008; Yahayu et al. 2013; Pattamadilok 2016), lignans (Syu et al. 2004; Manna et al. 2015), naphthoquinones (Gu et al. 2004; Yang et al. 2019), coumarins (Phatchana and Yenjai 2014), stilbene oligomers (Sahidin et al. 2017), prenylated flavonoids (Sohn et al. 2004), phenolic glycosides (Zeng et al. 2015), and anthraquinones (Ali et al. 2000). Planar phenolic compounds, such as anthraquinones can intercalate into DNA, inhibit topoisomerase II, and induce chromosomal damage in mammalian cells (Mueller and Stopper 1999; Bhakta and Siva 2012; Chakarov et al. 2014). Examples of pan-assay interference compounds (PAINS) or invalid metabolic panacea (IMP) are curcumin (**75**) and tannins (Bisson et al. 2016; Nelson et al. 2017). It is therefore necessary to determine the selectivity indices of antibacterial phenolic compounds. An antibacterial phenolic compound with a selectivity index >10 merits further pharmacological examination (Tamargo et al. 2015). This is the case for 2-methoxy-7-methyljuglone (**128**) (Gu et al. 2004), maritinone (**132**), 3,3′-biplumbagin (**135**) (Uc-Cachón et al. 2014), malabaricone A (**164**) (Orabi et al. 1991), and malabaricone B (**165**) (Sivadas et al. 2023). Phenolic products with unfavorable selectivity indexes can be used to synthesize less toxic antibacterial derivatives (Cham et al. 2023).

## Clinical potential

Several phenolic compounds identified in Asian and Pacific Angiosperms can combat bacterial infections *in vivo*. For example, ethyl gallate (**100**) administered orally at a dose of 50 mg/kg/day increased the survival rate of mice infected with *S. typhimurium* by more than 70% (Choi et al. 2014). Anacardic acid (**170**) and glabrol (**273**) were active against MRSA and VRE in insects, respectively (Wu et al. 2019; Saedtler et al. 2020). Furthermore, a very interesting aspect of phenolic compounds pharmacology is that they can be both antibacterial and anti-inflammatory or even immunomodulatory. The inflammatory response during infections caused by Gram-negative bacteria is owed, at least in part, by lipopolysaccharides which induce the secretion of nitric oxide and cytokines. Antibacterial phenolic compounds like euryacoumarin A (**30**) inhibit lipopolysaccharide-induced nitric oxide production in RAW264.7 (IC_50_: 35.6 μM) (Song et al. 2017). Vitexin (**323**) given at the dose of 400 µg/kg inhibited lipopolysaccharides-induced lung inflammation in mice (De Melo et al. 2005). Vitexin (**323**) given parenterally at the dose of 60 mg/kg to mice infected with *S. aureus* attenuated the production of pro-inflammatory cytokines (Chen et al. 2022). Baicalin (**321**) was able to protect mice against *S. typhimurium* infection, modulate bacterial virulence, and quell the host inflammatory response (Wu et al. 2018). Another example is [6]-gingerol (**167**) which exerted both antibacterial and immunomodulatory activity against *M. tuberculosis* in mice (Bhaskar et al. 2020).

Concerning the synergistic effect of antibacterial phenolic compounds with antibiotics *in vivo*, examples are chlorogenic acid (**4**) with levofloxacin against *K. pneumoniae* (Tan, Gao, et al. 2020), plumbagin (**127**) with colistin against colistin-resistant *P. aeruginosa* (Wang, Kong, et al. 2022), and sophoraflavanone G (**279**) with norfloxacin against effluxing antibiotic-resistant *S. aureus* (Sun et al. 2020). Baicalein (**298**) was synergistic with ampicillin against *Streptococcus suis* (Lu et al. 2021), with linezolid against *S. aureus* (Liu et al. 2020), and with myricetin (**305**) with oxacillin against MRSA (Pinto et al. 2020).

## Concluding remarks

The knowledge accumulated over the last decades highlights that the phenolic compounds with very strong antibacterial activity identified in the Angiosperms of Asia and the Pacific mainly come from fabids, often carry isoprene or long-chain alkyl groups, are often planar, with a molecular mass ranging from ∼200 to 400 g/mol, and are often amphiphilic. These products are mainly active against Gram-positive bacteria, and primarily target the cytoplasmic membrane, thus avoiding the development of resistance. 2-Methoxy-7-methyljuglone (**128**), malabaricone A (**164**), and malabaricone B (**165**) (Sivadas et al. 2023) with MIC values <1 µg/mL and selectivity indices >10 could potentially be developed as antibacterial agents as well as anacardic acid (**170**). Unlike commonly used antibiotics, some of these phenolic compounds are not only antibacterial or antibiotic potentiators but also anti-inflammatory or immunomodulators, such as [6]-gingerol (**167**), baicalin (**321**), and vitexin (**323**). The clinical development of antibiotics or antibiotic potentiators to treat pan-resistant bacteria from phenolic compounds from Asia and Pacific Angiosperms should come to light.
